# Mapping enhancer–gene regulatory interactions from single-cell data

**DOI:** 10.1038/s41588-026-02695-8

**Published:** 2026-08-03

**Authors:** Maya U. Sheth, Wei-Lin Qiu, X. Rosa Ma, Andreas R. Gschwind, Evelyn Jagoda, Anthony S. Tan, James Galante, Judhajeet Ray, Dulguun Amgalan, Hjörleifur Einarsson, Bram L. Gorissen, Danilo Dubocanin, Christopher S. McGinnis, Jacob Huang, Glen Munson, Kayla Brand, Ansuman T. Satpathy, Thouis R. Jones, Lars M. Steinmetz, Anshul Kundaje, Berk Ustun, Jesse M. Engreitz, Robin Andersson

**Affiliations:** 1 The Novo Nordisk Foundation Center for Genomic Mechanisms of Disease, Broad Institute of MIT and Harvard, Cambridge, MA USA; 2 Department of Genetics, Stanford University School of Medicine, Stanford, CA USA; 3 Basic Sciences and Engineering Initiative, Betty Irene Moore Children’s Heart Center, Lucile Packard Children’s Hospital, Stanford, CA USA; 4 Department of Bioengineering, Stanford University, Stanford, CA USA; 5 Department of Biology, Section for Computational and RNA Biology, University of Copenhagen, Copenhagen, Denmark; 6 Analytic and Translational Genetics Unit, Massachusetts General Hospital, Boston, MA USA; 7 Department of Pathology, Stanford University, Stanford, CA USA; 8 Parker Institute for Cancer Immunotherapy, San Francisco, CA USA; 9 Genome Biology Unit, European Molecular Biology Laboratory (EMBL), Heidelberg, Germany; 10 Stanford Genome Technology Center, Stanford University School of Medicine, Palo Alto, CA USA; 11 Department of Computer Science, Stanford University, Stanford, CA USA; 12 Halıcıoğlu Data Science Institute and Department of Computer Science and Engineering, University of California San Diego, San Diego, CA USA; 13 Gene Regulation Observatory, Broad Institute of MIT and Harvard, Cambridge, MA USA; 14 Stanford Cardiovascular Institute, Stanford University School of Medicine, Stanford, CA USA

**Keywords:** Genomics, Gene regulation, Computational biology and bioinformatics, Genetic association study

## Abstract

Mapping enhancers and their target genes in specific cell types is crucial for understanding gene regulation and human disease genetics. However, accurately predicting enhancer–gene regulatory interactions from single-cell datasets has been challenging. Here we introduce a family of classification models, scE2G, to predict enhancer–gene regulation. These models use features from single-cell assay for transposase-accessible chromatin with sequencing (ATAC-seq) or multiomic RNA and ATAC-seq data, and are trained on a CRISPR perturbation dataset including >10,000 evaluated element–gene pairs. We benchmark scE2G models against CRISPR perturbations, fine-mapped expression quantitative trait loci and genome-wide association study variant–gene associations and demonstrate state-of-the-art performance at prediction tasks across several cell types and categories of perturbations. We apply scE2G to build maps of enhancer–gene regulatory interactions in heterogeneous tissues and interpret noncoding variants associated with complex traits, nominating regulatory interactions linking *INPP4B* and *IL15* to lymphocyte count. The scE2G models will enable accurate mapping of enhancer–gene regulatory interactions across thousands of human cell types.

## Abstract (web summary)

scE2G is a family of models that predict enhancer–gene regulatory interactions from single-cell datasets and enable mapping of these interactions across diverse cell types and tissues.

## Main

The human genome encodes millions of regulatory elements called enhancers that tune gene expression in specific cell types^1–6^. Identifying which enhancers regulate which genes in which cell types (‘enhancer–gene regulatory interactions’) is critical for understanding gene regulation and the functions of disease-related genetic variants^7–10^. Yet, such maps are lacking for most human cell types.

Previously, we and others have built computational models that predict enhancer–gene regulatory interactions from bulk measurements of chromatin state and three-dimensional (3D) contacts^11–20^, including Activity-by-Contact (ABC)^12^ and ENCODE-rE2G^19^. These models have enabled creating genome-wide enhancer–gene maps in hundreds of human cell types and tissues^12,19^. However, their resolution is limited in complex tissues due to challenges in purifying individual cell types for bulk epigenomic assays.

Advances in single-cell assay for transposase–ATAC-seq and RNA sequencing (RNA-seq) technologies now enable accessing virtually any cell type and state across heterogeneous tissues^21–26^. Models using single-cell data could characterize enhancer–gene regulation in many more contexts than bulk models. Previous efforts toward this goal have relied largely on unsupervised strategies such as correlating the element accessibility with gene expression^27–36^. However, assessing the accuracy and consistency of these methods has been difficult due to the lack of robust benchmarking datasets and pipelines. Recent work by the ENCODE Consortium to collect gold-standard datasets of enhancer perturbations provides an opportunity to evaluate these models and to develop models based on supervised learning^12,19,37,38^.

Here we introduce scE2G—a family of single-cell enhancer-to-gene models that predict genome-wide enhancer–gene regulatory interactions from scATAC or multiomic scATAC and scRNA-seq data. We benchmark scE2G and ten other published models, using and extending pipelines developed with the ENCODE Consortium^19^. scE2G achieves state-of-the-art performance across diverse single-cell datasets and prediction tasks, including CRISPR perturbations, fine-mapped expression quantitative trait loci (eQTLs), and GWAS (genome-wide association study) variant–gene associations. We apply scE2G to map enhancer–gene regulatory interactions in 45 cell types and demonstrate its utility for interpreting risk variants for common diseases. scE2G models are robust to sequencing depth, cell number and assay type. Alongside expanding efforts to collect single-cell data, scE2G will facilitate efforts to build genome-wide enhancer maps in thousands of human cell types.

## Results

### Predicting regulatory enhancer–gene interactions from single-cell data

**Fig. 1:**
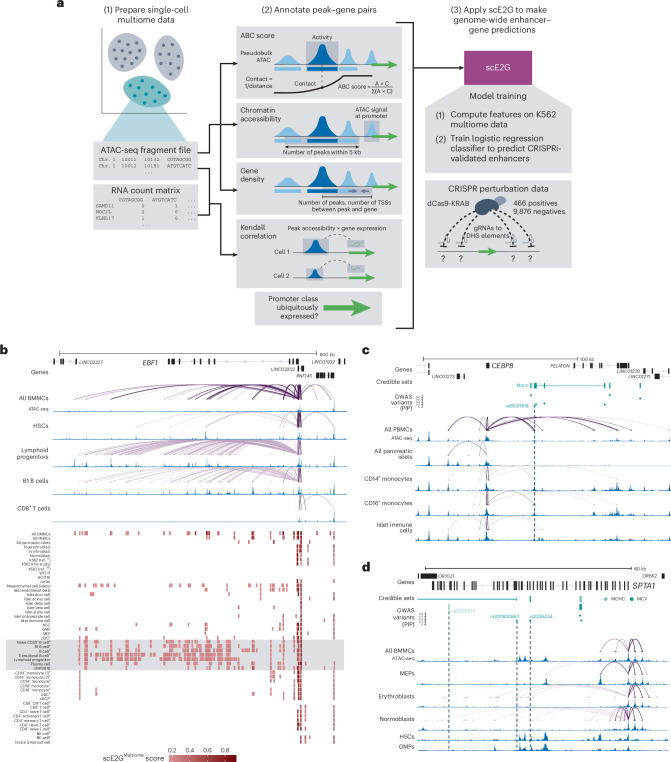
scE2G identifies enhancer–gene regulatory interactions from single-cell data across 45 cell types. **a**, Conceptual overview of using scE2G to predict enhancer–gene interactions from single-cell data for a selected cell type. **b**, scE2G^Multiome^ predictions for *EBF1* in combined BMMCs and four BMMC cell types: HSCs, lymphoid progenitors, B1 B cells and CD8^+^ T cells (chr. 1: 158330000–159250000, hg38). Locus plot: enhancer–gene arcs and normalized ATAC signals; heatmap: predicted *EBF1* enhancers across all cell types. Arc color/width and heatmap color indicate scE2G score magnitude. Shaded rows: B cell types with active regulatory landscapes, expected based on known dynamic *EBF1* expression during lymphopoiesis^55^ and B cell development^56^. ^a^ or ^b^ after the cell type denotes source from BMMC or PBMC datasets, respectively. ‘All BMMCs’ are predictions made on all combined BMMCs (see Methods, ‘Data visualization’ for locus plot details). **c**, scE2G^Multiome^ predictions for *CEBPB* in combined PBMCs, combined pancreatic islets, PBMC CD14^+^ monocytes, PBMC CD16^+^ monocytes and islet immune cells (chr. 20: 50145000–50310000, hg38). Noncoding fine-mapped GWAS variants with PIP > 10% and credible sets for monocyte count are shown. Vertical dashed line: variant rs80311618, which overlaps a myeloid-specific predicted enhancer. **d**, scE2G^Multiome^ predictions for *SPTA1* in combined BMMCs and three BMMC cell types: megakaryocytic-erythroid progenitors (MEPs), erythroblasts and normoblasts (chr. 1: 158605000–158702000, hg38). Normalized ATAC signals for HSCs and granulocyte-macrophage progenitors (GMPs), which have no predicted *SPTA1* enhancers, are shown for comparison. Noncoding fine-mapped GWAS variants with PIP > 10% and credible sets for mean corpuscular hemoglobin concentration (MCHC) and MCV, are also shown. Vertical dashed lines: variants overlapping erythroid-specific predicted enhancers: rs2022003 (MCHC), rs200830867 (MCV) and rs2246434 (MCV).

We developed two models to predict enhancer–gene regulatory interactions from single-cell data: scE2G^ATAC^, which uses scATAC-seq data, and scE2G^Multiome^, which uses paired scRNA and scATAC-seq data. Both models are logistic regression classifiers that take single-cell data as input and predict which chromatin-accessible elements act as enhancers to regulate which genes in a given cell population (Fig. 1a). We trained scE2G models using gold-standard CRISPR perturbation data in K562 cells, allowing application of the trained models to single-cell data from other cell types (Fig. 1a). In this way, scE2G makes cell-type-resolved predictions by analyzing data from one cell type or state at a time (for example, one cluster of cells from a single-cell dataset).

**Extended Data Fig. 1:**
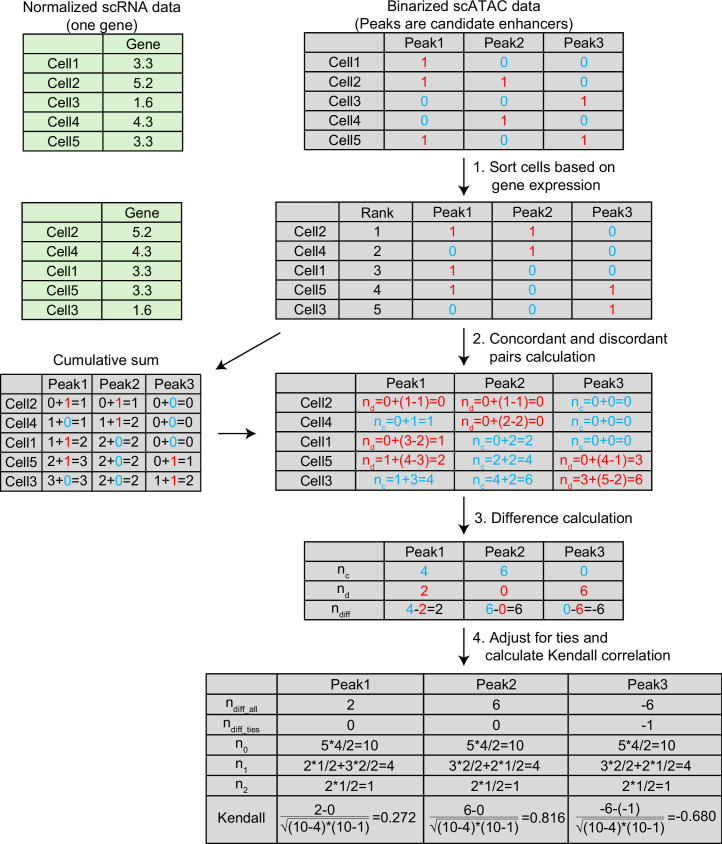
Step-by-step illustration of our algorithm for calculating the Kendall correlation between binarized element accessibility and continuous gene expression. Illustration of the step-by-step algorithm for calculating Kendall correlation between one gene and all candidate elements: 1. *Sort cells based on gene expression:* Organize the binarized scATAC-seq data in descending order based on gene expression levels. 2. *Concordant and discordant pairs calculation:* For each peak, calculate the number of concordant (n_c_) and discordant (n_d_) pairs for rank k: if accessibility[k] = 0, then n_c_[k] = n_c_[k-1] + CumulativeSum[k], n_d_[k] = n_d_[k-1]; if accessibility[k] = 1, n_d_[k] = n_d_[k-1] + (Rank[k] - CumulativeSum[k]), n_c_[k] = n_c_[k-1]. n_c_[0] = 0 and n_d_[0] = 0. 3. *Difference calculation:* Determine the difference between the number of concordant and discordant pairs for each peak: n_diff_ = n_c_ - n_d_. 4. *Adjust for ties and calculate Kendall correlation:* Perform steps 1–3 for all cells to compute n_diff_all_, and steps 2 and 3 for each group of cells with equal gene expression, taking the sum of n_diff_ over such groups, to compute n_diff_ties_. n_0_ = n(n - 1)/2, n_1_= ∑t_i_(t_i_-1)/2, n_2_ = ∑u_j_ (u_j_-1)/2; n is the number of cells, t_i_ and u_j_ represent the number of tied values in the i^th^ and j^th^ group of ties for the gene expression and element accessibility, respectively. Finally, Kendall correlation τ = (n_diff_all_ - n_diff_ties_)/√((n0-n1)⋅(n0-n2)). Each row represents a cell, and each column represents a gene or peak. Matrices with green backgrounds correspond to scRNA-seq data, while those with gray backgrounds represent intermediate matrices used in scATAC-seq data and Kendall correlation calculation. Red numbers indicate open ATAC accessibility, whereas blue numbers indicate closed ATAC accessibility.

**Extended Data Fig. 2:**
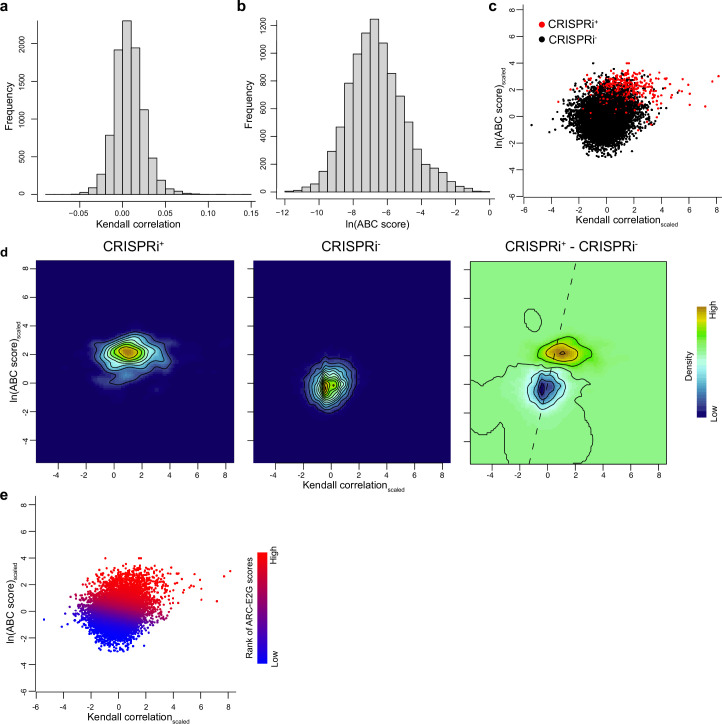
Integrating ABC score and Kendall correlation into ARC-E2G score. **a** and **b**. Histogram plots showing the distribution of the element-gene Kendall correlation (**a**) and ln(ABC score) (**b**) in CRISPR validated enhancer-gene pairs. **c**. Scatter plot showing the relationship between scaled ln(ABC score) and scaled Kendall correlation. We scaled values by subtracting the mean and then dividing by the standard deviation. Each point represents a CRISPR validated enhancer-gene pair. Red: Element-gene pairs with significant effects in the CRISPR experiment (CRISPR^+^). Black: Element-gene pairs without significant effects in the CRISPR experiment (CRISPR^−^). **d**. Density of CRISPR^+^ (left) and CRISPR^−^ (middle) element-gene pairs; and density difference between CRISPR^+^ and CRISPR^−^ element-gene pairs (right). The dashed line represents the linear regression line. **e**. We fit a linear regression between scaled ln(ABC score) and scaled Kendall correlations, and define the integrated Activity, Responsiveness and Contact (ARC)-E2G values along the linear regression line (Methods). Projected ARC-E2G score ranks to the same plot as in (**a**), with a color gradient from blue to red representing low to high ARC-E2G scores.

The scE2G^ATAC^ model uses six features: (1) an ABC score, with activity computed from pseudobulk scATAC-seq data and contact estimated as an inverse function of genomic distance; (2,3) quantitative measures of chromatin accessibility at or near the element (2) and the promoter (3); (4) genomic distance and (5) gene density between the element and the promoter; and (6) promoter class, stating whether the gene is expressed ubiquitously across cell types (Fig. 1a). Apart from the ABC score being adapted to single-cell data, this first set of features is similar to those used in the ENCODE-rE2G model we developed for bulk data^19^. scE2G^Multiome^ also includes the Kendall correlation between element accessibility and gene expression across single cells (Supplementary Note 1 and Extended Data Fig. 1), which we integrate with the ABC score as a feature termed the activity, responsiveness and contact (ARC)-E2G score to improve calibration across different sequencing depths (Extended Data Fig. 2 and Methods).

We trained scE2G models on a dataset of 10,342 genomic element–gene pairs tested with CRISPR in K562 erythroleukemia cells, which we aggregated previously from three studies^19^. This dataset includes 466 unique ‘positive’ element–gene pairs (CRISPR perturbation of the element led to significant decrease in gene expression) and 9,876 ‘negative’ pairs (no significant reduction in expression; at least 15–25% effect size). We computed features from multiomic single-cell RNA and ATAC-seq (10x Multiome) data in K562 cells^39^, and trained logistic regression classifiers on the CRISPR-labeled element–gene pairs. Using ‘hold-one-chromosome-out’ cross-validation evaluation, we used a ‘best-subsets’ strategy to identify a minimal set of features, described above, that achieve near-optimal classification performance (Supplementary Fig. 1a and Methods).

Once trained, scE2G models can be applied to predict interactions from data in new cell types to infer all genome-wide enhancer–gene regulatory interactions, with each element–gene pair receiving a score indicating the predicted probability of a regulatory effect (Supplementary Fig. 1b).

**Extended Data Fig. 3:**
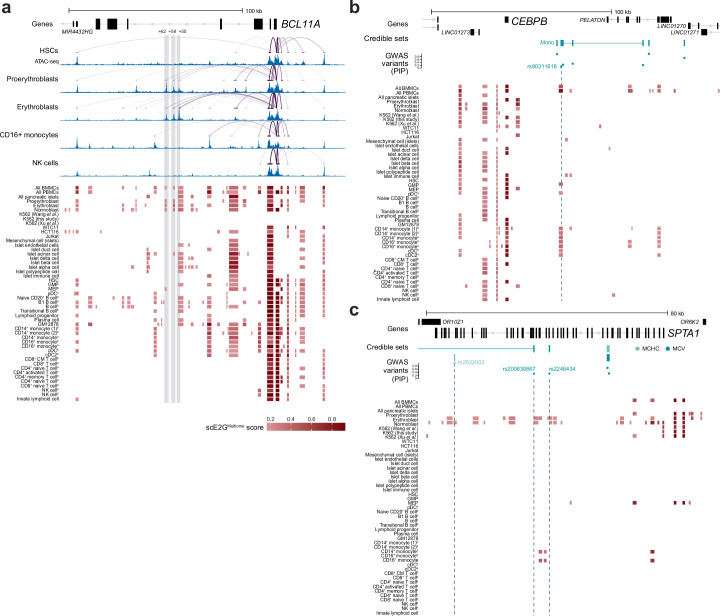
Cell-type-resolved enhancer-gene regulatory predictions by scE2G^Multiome^. **a**, scE2G^Multiome^ predictions for *BCL11A* (chr1:158,330,000–159,250,000, hg38) for five BMMC cell types: hematopoietic stem cells (HSCs), proerythroblasts, erythroblasts, CD16^+^ monocytes, and natural killer (NK) cells. Heatmap shows predicted *BCL11A* enhancers across all cell types. Arc color/width and heatmap color indicate scE2G score magnitude. Grey rectangles overlap three known erythroid-specific enhancers for *BCL11A* (labels indicate distance to TSS in kb) which are targets for gene editing therapies treating β-hemoglobinopathies^91^. scE2G^Multiome^ predictions for these three elements are specific to cells with erythroid lineage. See Methods, ‘Data visualization’ for locus plot details. **b**, Heatmap as in **a** showing scE2G^Multiome^ predictions for *CEBPB* across all cell types at chr20:50,145,000-50,310,000 (hg38), the locus shown in Fig. 1c. scE2G identifies many and varying enhancers across myeloid progenitors and monocytes for *CEBPB*, consistent with its known regulation in myeloid cells^57^. Noncoding fine-mapped GWAS variants with PIP > 10% and credible sets for monocyte count are shown. Vertical dashed line indicates variant rs80311618, which overlaps a myeloid-specific predicted enhancer. **c**, Heatmap as described in **a** showing scE2G^Multiome^ predictions for *SPTA1* across all cell types at chr1:158,605,000-158,702,000 (hg38), the locus shown in Fig. 1d. Noncoding fine-mapped GWAS variants with PIP > 10% and credible sets for mean corpuscular hemoglobin concentration (MCHC) and mean corpuscular volume (MCV), are shown. Vertical dashed lines indicate variants overlapping erythroid-specific predicted enhancers: rs2022003, (MCHC), rs200830867 (MCV), rs2246434 (MCV).

To demonstrate the utility of scE2G, we applied scE2G^Multiome^ to build maps of enhancer–gene regulatory interactions in 45 different cell types (defined here as a cell cluster annotated based on expression of marker genes) including those from human peripheral blood mononuclear cells (PBMCs), bone marrow mononuclear cells (BMMCs) and pancreatic islets (Fig. 1b and Supplementary Fig. 2). For each cell type, we (1) pseudobulk scATAC-seq data to call peaks and compute the ABC score and other chromatin accessibility-derived features; (2) compute element–gene correlations across using the single-cell RNA and ATAC-seq data and (3) apply the pretrained model to score each element–gene pair. The resulting predictions capture both differences and similarities in regulatory interactions across cell types—features that are obscured in predictions made using bulk datasets. The value of applying scE2G to single-cell data is demonstrated by the predictions of regulatory interactions for lineage-relevant genes such as *EBF1*, *CEBPB* and *SPTA1*, which align with known cell-type-specific regulatory patterns (Fig. 1b–d and Extended Data Fig. 3; see section ‘Cell-type resolution of scE2G predictions’ for quantitative analysis).

### Benchmarking of scE2G models

**Fig. 2:**
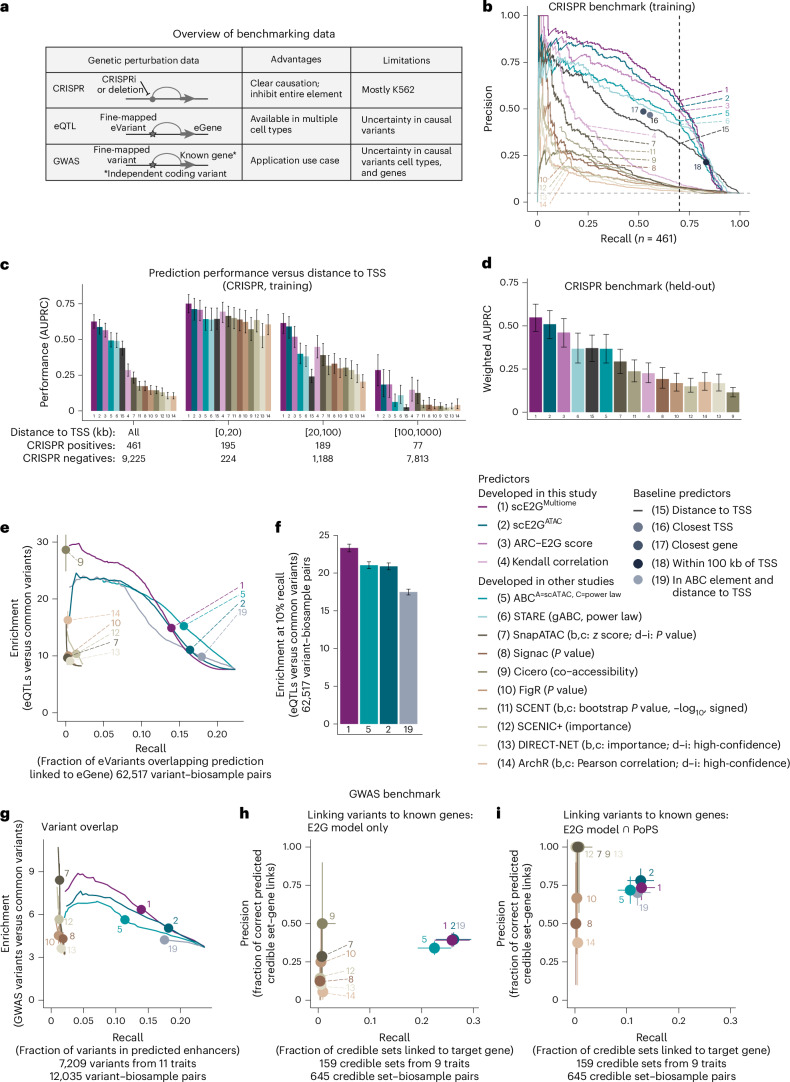
Comparison of scE2G in benchmark tasks. **a**, Overview of CRISPR, eQTL and GWAS benchmarking data (modified from Gschwind and colleagues^19^). **b**, Precision–recall curves for K562 CRISPR data (9,225 tested pairs; 461 positives; distance to TSS < 1 Mb). Curves: continuous predictors; dots: binary predictors. Dashed lines: 70% recall (vertical); baseline precision (horizontal). **c**, AUPRC of continuous predictors on the K562 CRISPR dataset versus TSS distance. Error bars: mean ± 95% confidence intervals inferred by bootstrap (10,000 iterations). **d**, AUPRC (pairs weighted by direct effect probability; Methods) of continuous predictors on held-out CRISPR dataset (pairs from K562, GM12878, HCT116, Jurkat and WTC11 cells; 4,175 tested pairs; 189 positives; distance to TSS < 1 Mb). Error bars: mean ± 95% confidence intervals inferred by bootstrap (10,000 iterations). **e**, Enrichment-recall curves for predicting fine-mapped eQTLs (matching cell types; Supplementary Table 4). Enrichment: ratio of fine-mapped distal noncoding eQTLs (PIP > 50%) to distal noncoding common variants overlapping predicted enhancers. Recall: fraction of eQTL variants overlapping enhancers linked to correct gene. Curves: performance at ~100 thresholds; points: performance at suggested model thresholds; error bars at points: 95% confidence intervals calculated using normal approximation on log-transformed enrichment ratios. **f**, Enrichment of variants at 10% recall for the same variant-cell type pairs as in **e**. Error bars: 95% confidence intervals calculated as in **e**. **g**, Enrichment-recall curves for predicting fine-mapped GWAS variants for 11 blood-related traits in predictions from matching cell types (Supplementary Table 5). Enrichment: ratio of GWAS variants (PIP > 10%) to distal noncoding common variants in predicted enhancers. Recall: fraction of GWAS variants overlapping enhancers. Curves, points and error bars as in **e**. **h**, Precision and recall for linking credible sets to known causal genes (inferred from independent signals with coding variants) with model predictions binarized at the suggested threshold. Top two genes per credible set were considered positive. Error bars: 95% confidence intervals from Wilson score intervals of precision (vertical) and recall (horizontal). **i**, As **h**, but intersecting predicted genes with top two PoPS-linked genes per credible set.

We benchmarked scE2G and existing single-cell models of enhancer–gene regulation using three orthogonal datasets: CRISPR enhancer perturbations, fine-mapped eQTLs and fine-mapped GWAS variants^19^. Together, these benchmarks evaluate enhancer maps in orthogonal and complementary ways (Fig. 2a).

We compared scE2G models with distance-derived baselines and ten models developed in previous work (Supplementary Table 1 and Supplementary Note 2): (1) ABC-based models ABC^12^ and STARE (gABC)^40^, which estimate element activity and 3D contact frequency using pseudobulk ATAC signal and power law genomic distance-decay, respectively; (2) correlation-based models Signac^33^, Cicero^28^, ArchR^27^ and FigR^30^, which compute Pearson or Spearman correlation between element accessibility and gene expression (or promoter accessibility); (3) regression-based models SnapATAC^34^, SCENT^32^, DIRECT-NET^29^ and SCENIC+^31^, which learn quantitative relationships between the accessibility of one or more elements and gene expression. An important distinction between these models is that some (scE2G, ABC, STARE, SCENT) analyze each cell type independently, whereas others predict interactions by comparing across all cell types in a dataset.

First, using the K562 CRISPR perturbation dataset and a deeply sequenced K562 10x Multiome dataset^39^ (Supplementary Fig. 2), we evaluated model accuracy at predicting causal enhancer–gene regulatory interactions in a given cell type. Because scE2G models were trained on this dataset, we used hold-one-chromosome-out cross-validation scores for this and all subsequent benchmarking against the dataset. scE2G^Multiome^ and scE2G^ATAC^ significantly outperformed all other models across all metrics (Supplementary Tables 2 and 3), with scE2G^Multiome^ outperforming scE2G^ATAC^ (Fig. 2b,c; *P*_bootstrap_ = 3 × 10^−4^ for delta area under the precision–recall curve (AUPRC); *P*_bootstrap_ = 1 × 10^−16^ for delta precision) and scE2G^ATAC^ outperforming ABC (Fig. 2b,c; *P*_bootstrap_ = 1 × 10^−16^ for delta AUPRC and precision). These trends were stronger for distal element–gene pairs (Fig. 2c). Notably, only scE2G and ABC-based models outperformed distance baselines. These results were consistent across two additional K562 10x Multiome datasets (Supplementary Fig. 3) and when using each model’s default settings (Supplementary Fig. 4). Furthermore, scE2G^Multiome^ achieved similar performance to the ENCODE-rE2G model based on bulk DNase-seq data (delta AUPRC = −0.022, *P*_bootstrap_ = 0.139; delta precision = −2.9%, *P*_bootstrap_ = 0.036; Supplementary Note 3).

**Extended Data Fig. 4:**
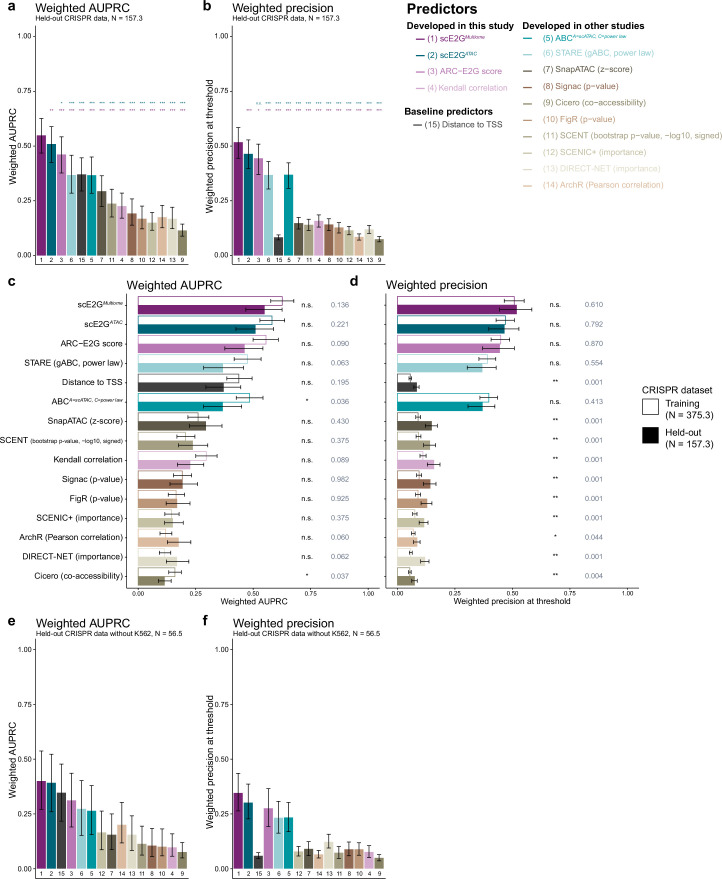
CRISPR benchmarking using held-out CRISPR datasets from five cell types. **a**, AUPRC (pairs weighted by direct effect probability, Methods) of continuous predictors on held-out CRISPR dataset (157.3 weighted positives), as in Fig. 2d. Error bars: mean ± 95% confidence intervals inferred via bootstrap (1,000 iterations). One, two, and three stars indicate two-sided *P*_bootstrap_ < 0.05, 0.01, and 0.001 inferred via paired bootstrap (1,000 iterations), respectively, for the significance of delta AUPRC between scE2G^Multiome^ (purple stars) or scE2G^ATAC^ (teal stars) and the predictor beneath the stars; n.s. indicates non-significant difference (*P* > 0.05); two-sided *P* values calculated from the paired bootstrap distribution without adjustment for multiple comparisons. **b**, As **a**, but showing weighted precision at the model threshold. **c**, Weighted AUPRC of predictors on training (unfilled bars) and held-out (filled bars) CRISPR datasets. For a fair comparison with the held-out dataset, the training dataset was modified by 1) removing positives with < 5% effect size and 2) removing positives with no/low H3K27ac (Methods). Error bars: mean ± 95% confidence intervals inferred via bootstrap (1,000 iterations). One, two and three stars represent two-sided *P*_bootstrap_ less than 0.05, 0.01, and 0.001, respectively, for significance of delta AUPRC between the training and held-out datasets, inferred via bootstrap (1,000 iterations); n.s. indicates non-significant difference (*P* > 0.05); two-sided *P* values calculated from bootstrap distribution without adjustment for multiple comparisons and shown after stars. **d**, As **c**, but showing weighted precision at model threshold. **e**, As **a**, but showing weighted AUPRC on the subset of the held-out CRISPR dataset from non-K562 cell types (56.5 weighted positives). **f**, As **b** but showing weighted precision at the model threshold on the subset of the held-out CRISPR dataset from non-K562 cell types.

To assess the generalizability of scE2G to other CRISPR datasets, we benchmarked the models on a held-out CRISPR dataset comprising 4,175 tested element–gene pairs (189 positives) from five cell types^41–47^ (Fig. 2d and Extended Data Fig. 4). Due to differences in element selection and screen design for the held-out dataset, we calculated performance metrics weighting each element–gene pair by the probability it represents a direct effect (Methods). Again, scE2G models achieved significantly better AUPRC and precision than all other models (Fig. 2d and Extended Data Fig. 4a,b). Furthermore, scE2G performance did not differ significantly between the training and held-out datasets (Extended Data Fig. 4c,d).

**Extended Data Fig. 5:**
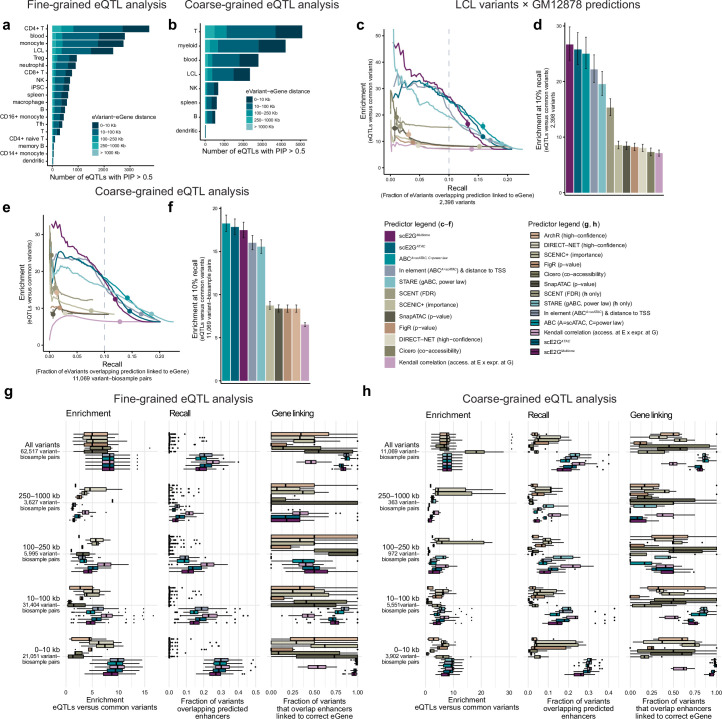
Benchmarking scE2G using fine-mapped eQTLs. **a**, Number of fine-mapped eQTL variants (eVariants), stratified by eVariant–eGene distance, for each eQTL biosample considered in the ‘fine-grained’ eQTL analysis shown in Fig. 2e, f (*for example*, eQTLs in CD4^+^ naive T cells matched to model predictions from T cell types, Supplementary Table 4). **b**, Number of eVariants, stratified by eVariant–eGene distance, for each eQTL biosample considered in a ‘coarse-grained’ eQTL analysis (*for example*, eQTLs from T cells matched to model predictions from T cell type ‘supergroup’, Supplementary Table 4). The ‘coarse-grained’ eQTL analysis is shown to compare all single-cell models on the same set of matched eQTL-model biosamples. **c**, Enrichment-recall curves of model predictions in GM12878 cells at predicting eQTLs from lymphoblastoid-like cell lines (LCLs). Dashed vertical line: 10% recall. Error bars at points: 95% confidence intervals from log enrichment. **d**, Enrichment of LCL eVariants at 10% recall in GM12878 model predictions. Error bars: 95% confidence intervals calculated using normal approximation on log-transformed enrichment ratios. **e**, Enrichment-recall curves for the ‘coarse-grained’ eQTL analysis as described in **b**. Curves: performance at ~100 thresholds; points: performance at suggested model thresholds. Dashed vertical line: 10% recall. Error bars at points: 95% confidence intervals calculated as described in **d**. **f**, Enrichment of variants at 10% recall for the ‘coarse-grained’ eQTL analysis in **e**. **g**, Box plots showing enrichment, recall (not accounting for correct eGene-linking), and fraction of variants overlapping an enhancer linked to the correct eGene given that they overlap an enhancer, for the ‘fine-grained’ eQTL analysis; stratified by eVariant–eGene distance. Box plot elements: center line: median; lower hinge: first quartile; upper hinge: third quartile; whiskers extend from corresponding hinge to most distant value no further than 1.5× interquartile range from the hinge; data beyond the end of the whiskers plotted individually. **h**, As **g**, but for the ‘coarse-grained’ eQTL analysis.

Next, we evaluated how well the models identified fine-mapped eQTL variant–gene links (posterior inclusion probability (PIP) > 50%) from 19 biosamples^48^ using predictions from 32 matching cell types (Fig. 2e, Extended Data Fig. 5 and Supplementary Table 4). Fine-mapped eQTL variant–gene links are complementary to CRISPR perturbation data because they reflect naturally occurring genetic variation and are available in many more cell types and tissues. When binarizing predictions by their suggested thresholds, scE2G achieved the highest recall and strong enrichment (13.9% recall and 14.9-fold enrichment for scE2G^Multiome^), far outperforming the next-best non-ABC model, SCENIC+ (1.4% recall and 10.3-fold enrichment) and comparable to previous benchmarks of models using bulk data^19^ (10–20%, Supplementary Note 3 and Supplementary Fig. N2c). When evaluating models across all score thresholds, only ABC-based models achieved a recall greater than 2%. At a fixed recall of 10%, scE2G^Multiome^ enhancers were significantly more enriched in eQTLs than all other models (Fig. 2f; *P*_adjusted_ = 6.72 × 10^−10^ between scE2G^Multiome^ and the next-best model, two-sided Bonferroni-adjusted *z*-test on delta log enrichment).

**Extended Data Fig. 6:**
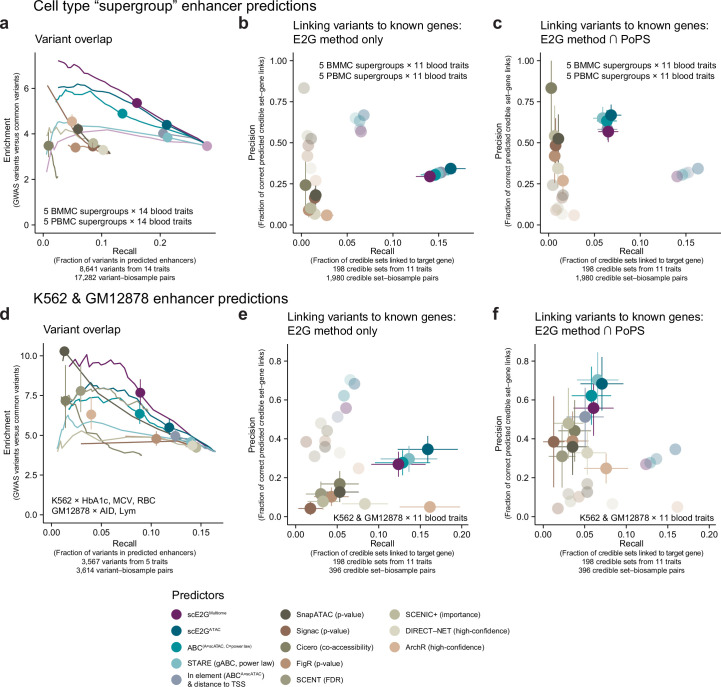
Benchmarking scE2G using fine-mapped GWAS variants. **a**, Enrichment-recall curves at predicting fine-mapped GWAS variants from 14 blood-related traits for enhancer predictions from 10 BMMC and PBMC cell type ‘supergroups’ (Supplementary Table 5), shown to compare all single-cell models on the same set of biosamples. Curves: performance at ~100 score thresholds; points: performance at suggested model thresholds; error bars: 95% confidence intervals calculated using normal approximation on log-transformed enrichment ratios. **b**, Precision and recall at linking credible sets (11 blood-related traits) to causal genes (inferred from independent signals with coding variants) for binarized enhancer predictions from 10 cell type supergroups (Supplementary Table 5). Top two genes per credible set were considered positive. Error bars: 95% confidence intervals from two-sided Wilson score intervals of precision (vertical) and recall (horizontal). Faded points: performance after intersecting predictions with top two PoPS-linked genes. **c**, As **b**; full color points indicate performance with PoPS intersection; faded points indicate performance without PoPS. **d**, Enrichment-recall curves as in **a** showing overlap of fine-mapped GWAS variants for hemoglobin A1c (HbA1c), mean corpuscular volume (MCV), and red blood cell count (RBC) in K562 predictions and variants for autoimmune disorders (AIDs) and lymphocyte count (Lym) in GM12878 predictions. **e**, Precision and recall at linking credible sets to known causal genes for binarized enhancer predictions, as in **b**. Enhancer predictions for K562 and GM12878 were combined and evaluated against credible sets from 11 blood-related traits (Supplementary Table 5). Faded points indicate performance of the models when predictions are intersected with top two PoPS-linked genes. Evaluation of predictions in K562 and GM12878 cells is shown as a direct comparison to our previous evaluation of bulk predictors^19^. **f**, As **e**, but full color points indicate performance with PoPS intersection and faded points indicate performance without PoPS.

Finally, we assessed each model’s ability to capture fine-mapped GWAS variants and link credible sets to target genes, using a dataset of 15,648 fine-mapped variants for 11 traits (PIP > 10%)^49^ and predictions from 28 cell types from PBMC and BMMC 10x Multiome datasets (Fig. 2g–i, Extended Data Fig. 6 and Supplementary Table 5). Because GWAS variants impact more highly constrained genes and tend to be further from their target gene transcription start site (TSS) than eQTLs^50^, this benchmark evaluates models on a complementary set of genetic ‘perturbations.’ In previous bulk-model benchmarks, recall and enrichment ranged from 5% to 15% and from 5-fold to 15-fold, respectively^19,51^ (Supplementary Note 3 and Supplementary Fig. N2e). When evaluating binarized predictions, only scE2G and other ABC-based models achieved recall and enrichment within comparable ranges (Fig. 2g). When evaluating models for linking variants to likely causal genes (nearby genes with independent coding variants associated with the same traits^52^; Methods), scE2G again achieved performance in a range comparable to bulk models (25–50% recall, 25–75% precision), with higher recall and similar precision to non-ABC-based models (Fig. 2h). The same trend was observed when intersecting model-predicted genes with those prioritized by the polygenic priority score (PoPS), which is based on gene function^52^: scE2G^Multiome^ achieved a recall of 24.2% and 12.9% before and after intersecting with PoPS, respectively, whereas non-ABC models did not surpass a recall of 0.8% in either case (Fig. 2i).

Together, these results demonstrate that scE2G consistently outperforms existing single-cell models across several benchmarking tasks and achieves similar performance to the ENCODE-rE2G model based on bulk DNase-seq data (Supplementary Note 3). Each benchmarking dataset has strengths and limitations: (1) CRISPR perturbations most directly identify links between enhancers and genes by characterizing the effects of inhibiting an entire element on gene expression in a specific cell type. However, the vast majority of CRISPR datasets were collected in K562 cells, and they may contain element–gene links attributable to indirect effects or mechanisms other than enhancers^12^. (2) Fine-mapped eQTL data are available in many primary cell types, but there is uncertainty in identifying the causal variant, and the absolute recall is low because eQTL variants can act through other mechanisms beyond affecting enhancers^53^. (3) Interpreting GWAS signals is an important use case, but there is uncertainty in causal variants, causal cell types, and the ‘known’ genes as annotated by independent coding variants^52^ (Fig. 2a). The strong and consistent performance of scE2G across these three complementary benchmarks covering many cell types and input datasets suggests that the model has indeed learned predictive and generalizable rules of enhancer–gene regulation.

### Model interpretation

**Fig. 3:**
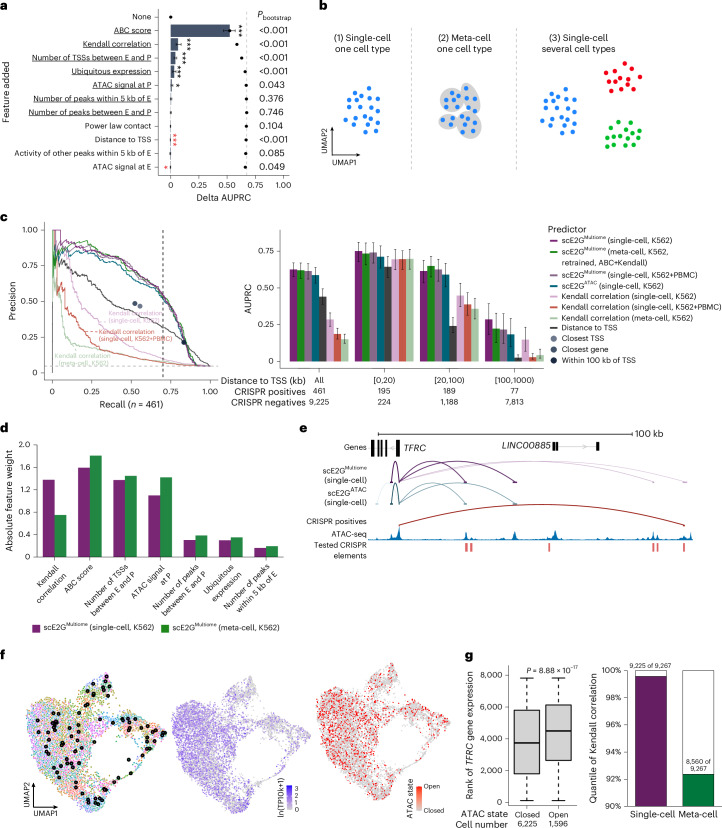
Interpretation of scE2G and the Kendall correlation. **a**, Forward sequential feature selection for scE2G^Multiome^ on the CRISPR benchmark (all element–gene pairs). Bars: delta AUPRC from adding each feature; dots: accumulated delta AUPRC; dashed line: total accumulated AUPRC. Error bars: mean ± 95% confidence intervals inferred by paired bootstrap (1,000 iterations). Two-sided *P* values were derived from bootstrap distribution with no adjustment for multiple comparisons: *P < 0.05 and ***P < 0.001; black/red asterisks: positive/negative delta, respectively; exact *P* value indicated to the right of each bar. Underlines: features used in the final model. E, element; P, promoter. **b**, Three strategies for computing Kendall correlation between gene expression and ATAC accessibility: (1) single cells within one cell type (single-cell, K562), (2) meta-cells within one cell type (meta-cell, K562) and (3) single cells across several cell types (single-cell, K562+PBMC). UMAP, uniform manifold approximation and projection. **c**, CRISPR benchmarking (distance to TSS < 1 Mb) for Kendall correlation calculated using each strategy and corresponding scE2G models. Left: precision–recall curves; plot elements as in Fig. 2b. Right: AUPRC of predictors versus distance to TSS. Error bars: mean ± 95% confidence intervals inferred by bootstrap (10,000 iterations). scE2G^Multiome^ (meta-cell, K562, retrained, ABC+Kendall) indicates that ABC score and Kendall correlation (meta-cell) were separate features during training. **d**, Absolute feature weights in scE2G^Multiome^ models trained on single-cell versus meta-cell data. **e**, scE2G^Multiome^ (single-cell) and scE2G^ATAC^ predictions for *TFRC* in K562 (chr. 3: 196070000–196197500, hg38). Arc color/width: scE2G score magnitude; red arc: CRISPR-validated enhancer (chr. 3: 196193510–196195038) regulating *TFRC* predicted only by scE2G^Multiome^ (single-cell). Also shown: normalized ATAC for K562 (ref. ^39^) and all CRISPR-tested elements. **f**, *TFRC* expression and accessibility of enhancer chr. 3: 196193510–196195038 indicate stochastic transcriptional regulation and associated enhancer accessibility across single cells. Left: assignment of single cells to SEACells^70^ meta-cells; meta-cells and corresponding single cells shown in same color. Middle: single-cell *TFRC* expression. Right: binarized single-cell ATAC accessibility. **g**, Comparison of Kendall correlation of *TFRC* expression with chr. 3: 196193510–196195038 accessibility for single-cell versus meta-cell data. Left: box plot showing gene expression rank for single cells with open versus closed ATAC state; *P* value from two-sided Wilcoxon test. Box plot elements: middle line: median; lower hinge: first quartile; upper hinge: third quartile; whiskers extend from minimum to maximum value, which are both closer than 1.5× the IQR from the hinge. Right: barplot showing quantile of Kendall correlation across 9,267 CRISPR-tested pairs using single-cell versus meta-cell data; higher rank indicates larger value.

In developing the final scE2G^Multiome^ model presented above, we analyzed the importance of model features to identify a minimal set of features needed for good performance and thereby increase their applicability in practice. We performed three feature importance analyses using the CRISPR benchmark: evaluating all subsets of the 11 potential input features, sequential feature selection and ablation-based feature importance (Fig. 3a and Supplementary Fig. 5). In each analysis, the ABC score, Kendall correlation and promoter class (whether the gene is expressed ubiquitously) had large significant effects on AUPRC and precision at 70% recall. We selected these features along with four others related to genomic distance and ATAC signal that yielded optimal performance for the final scE2G^Multiome^ model presented above (Fig. 3a).

Compared to the bulk ENCODE-rE2G model^19^, the main feature added to the scE2G^Multiome^ model is the Kendall correlation between element accessibility (binarized values) and gene expression (continuous values) in single cells (Supplementary Note 1). Incorporating the Kendall correlation improved the model’s ability to predict long-range enhancer–gene regulatory interactions (for example, >100 kb; Fig. 2c and Supplementary Fig. 5c), significantly improving over the ABC score alone (delta AUPRC = 0.223; *P*_bootstrap_ = 1 × 10^−16^) or the scE2G^ATAC^ model (delta AUPRC = 0.102; *P*_bootstrap_ = 6.0 × 10^−4^).

For the scE2G^Multiome^ model, the Kendall correlation is computed across single cells within a cell type (Fig. 1a). The Kendall correlation was chosen from six tested metrics due to its high predictive performance and computational tractability (Supplementary Note 1). In contrast to measuring element–gene associations across meta-cells or cells spanning several cell types^27–31,33,34^, which are the approaches taken by previous methods such as Signac^33^ and SnapATAC^34^, we found that computing correlations within a single cell type yielded the best performance (Fig. 3b–d). This suggests that stochastic variation in enhancer–gene co-activities across single cells within a cell type is informative for linking enhancers to their target genes. Consistent with this interpretation, enhancer–gene pairs predicted correctly by scE2G^Multiome^ but not by scE2G^ATAC^, which does not use Kendall correlation, did not form apparent K562 subtype clusters (Fig. 3e,f) and displayed reduced correlations across meta-cells (Fig. 3g). These correlations may reflect a temporal relationship between enhancer accessibility and gene transcription^54^. Therefore, the improved performance of scE2G^Multiome^ over scE2G^ATAC^ appears to stem from capturing regulatory heterogeneity across single cells, which models correlating across cell types or meta-cells may miss.

Finally, we identified categories of CRISPR pairs that scE2G predicts with varying accuracy. Pairs that are more distal, have weaker CRISPR effect size, lack canonical enhancer signatures or target ubiquitously expressed genes are more difficult to predict (Supplementary Note 4 and Supplementary Fig. N3). These types of pair tend to have lower positive rates, are underrepresented in the training data, may involve distinct regulatory mechanisms not modeled by scE2G, are more likely to reflect indirect effects and/or are experimentally challenging to detect due to limited statistical power (Supplementary Note 4).

### Performance across sequencing depths

Models predicting interactions should be robust to variation in sequencing depth, which can vary dramatically across both datasets and cell types within a single dataset. For example, the datasets used for benchmarking (Fig. 2) included cell types with 3,960 to 37,442 ATAC fragments per cell and 108 to 22,525 cells, spanning over two orders of magnitude in the total fragments per cell type.

**Fig. 4:**
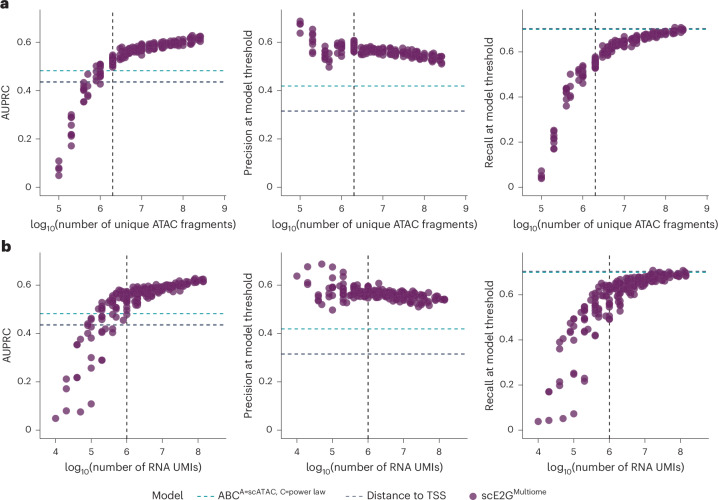
Performance of scE2G across sequencing depths. **a**, AUPRC (left), precision at the model threshold (center) and recall at the model threshold (right) of scE2G^Multiome^ on the CRISPR benchmark (element–gene pairs with all distance to TSS) across ATAC fragment counts with varying UMI and cell counts. Performance of distance to TSS (gray line) and ABC on the full-depth dataset (teal line, the next-best model after scE2G^Multiome^ and scE2G^ATAC^) are shown for reference. Vertical dashed lines: 2 million fragments. **b**, Same as **a**, but showing performance of scE2G^Multiome^ across RNA UMI counts with varying fragment and cell counts. Vertical dashed lines: 1 million UMIs.

**Extended Data Fig. 7:**
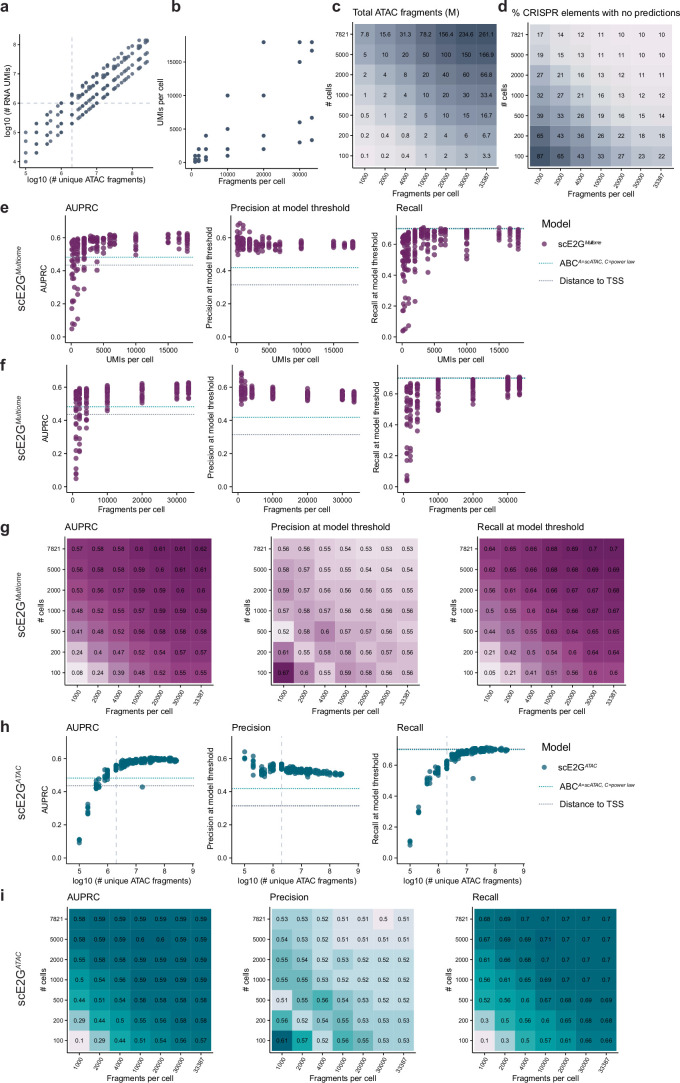
Robustness of scE2G to sequencing depth. **a**, Scatterplot showing number of ATAC fragments and RNA UMIs in 196 downsamples of Xu et al.^39^ K562 10x Multiome dataset (seven values of cells per downsample). **b**, Scatterplot showing 28 combinations of fragments per cell and UMIs per cell covered in downsamples (ATAC fragment to RNA UMI ratios of 1, 2, 5, and 10; representative of heterogeneous multiome datasets). **c**, Heatmap showing number of ATAC fragments in the tested downsamples. **d**, Heatmap showing percent of elements tested in the CRISPR benchmark (most of which are accessible) that do not overlap scE2G candidate elements (overlap defined as sharing at least 50% of base pairs). At low fragment counts, recall of CRISPR-tested elements markedly decreases due to degrading quality of ATAC peak calls; RNA sequencing depth does not affect this metric. **e**, AUPRC, precision at model threshold, and recall at model threshold of scE2G^Multiome^ on the CRISPR benchmark as a function of UMIs per cell. Grey line: performance of distance to TSS; teal line: performance of ABC on full-depth dataset (next best model after scE2G^Multiome^ and scE2G^ATAC^). **f**, As **e**, but showing performance of scE2G^Multiome^ as a function of ATAC fragments per cell. **g**, Heatmaps showing CRISPR benchmark performance for scE2G^Multiome^ across downsamples with different coverage (performance averaged across four downsamples with the same cell count and fragments per cell and different ATAC-to-RNA ratios). **h**, As **e**, but showing performance of scE2G^ATAC^. Vertical dashed lines: 2 million fragments. **i**, As **g**, but showing performance of scE2G^ATAC^ (performance averaged across four downsamples with the same cell count and fragments per cell).

To evaluate the robustness of scE2G to this variation, we downsampled K562 10x Multiome data^39^ while varying cell counts, ATAC fragments per cell and RNA unique molecular identifiers (UMIs) per cell (Extended Data Fig. 7a–c) and evaluated scE2G predictions on each downsampled dataset using the CRISPR benchmark (Fig. 4, Supplementary Fig. 6 and Extended Data Fig. 7).

**Extended Data Fig. 8:**
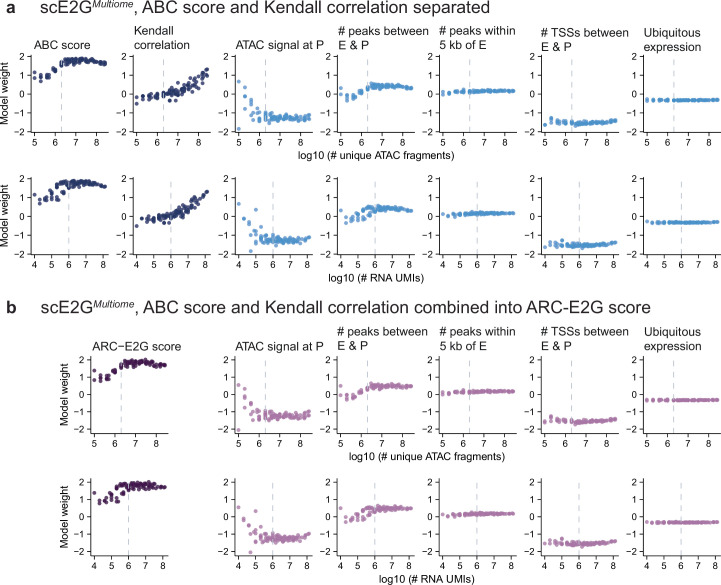
Robustness of scE2G model weights to sequencing depth. **a**, Feature weights for scE2G^Multiome^ models with ABC and the Kendall correlation as independent features trained on each of 98 downsamples. The downsamples correspond to the 2:1 and 10:1 ATAC fragments to RNA UMIs ratio for the 49 combinations of cell and fragment counts shown in Extended Data Fig. 7. Model weights are shown as a function of ATAC fragments (top row) and RNA UMIs (bottom row) per downsample. Dashed vertical lines: 2 million ATAC fragments (top row), 1 million RNA UMIs (bottom row). Note that the model weight for the Kendall correlation increases beyond the 2 million fragment or 1 million UMI mark. **b**, As **a**, but for scE2G^Multiome^ models with ARC-E2G integrating the ABC score and the Kendall correlation features. Note that the feature weight for ARC-E2G score is stable beyond 2 million fragments and 1 million RNA UMIs.

Above a minimum of 2 million ATAC fragments and 1 million RNA UMIs (corresponding to ~100 cells), scE2G^Multiome^ demonstrates robust performance, with AUPRC and recall improving modestly at higher coverage due to the increased contribution of the element–gene Kendall correlation signal. This robustness is achieved by combining the ABC score and Kendall correlation into an integrated feature that is stable across sequencing depths (Extended Data Fig. 8 and Methods).

### Cell-type resolution of scE2G predictions

We next explored properties of scE2G predictions across cell types from three single-cell multiome datasets: PBMCs, BMMCs and pancreatic islets (Supplementary Fig. 7 and Supplementary Tables 6 and 7). In each of the 39 cell types passing sequencing depth thresholds (range, 4.9–822 million ATAC fragments; Supplementary Fig. 7a), scE2G^Multiome^ predicted an average of 48,758 enhancer–gene links (range, 41,947–54,180; Supplementary Fig. 7b), involving 36,259 unique enhancers (Supplementary Fig. 7c) and 8,658 genes with at least one distal enhancer (Supplementary Fig. 7f). The average enhancer was located 86.3 kb from its target gene promoter (Supplementary Fig. 7h). Across all cell types, scE2G^Multiome^ predicted, on average, 5.6 enhancers regulating each expressed gene, and each enhancer regulating 1.3 genes (Supplementary Fig. 7i,j).

**Extended Data Fig. 9:**
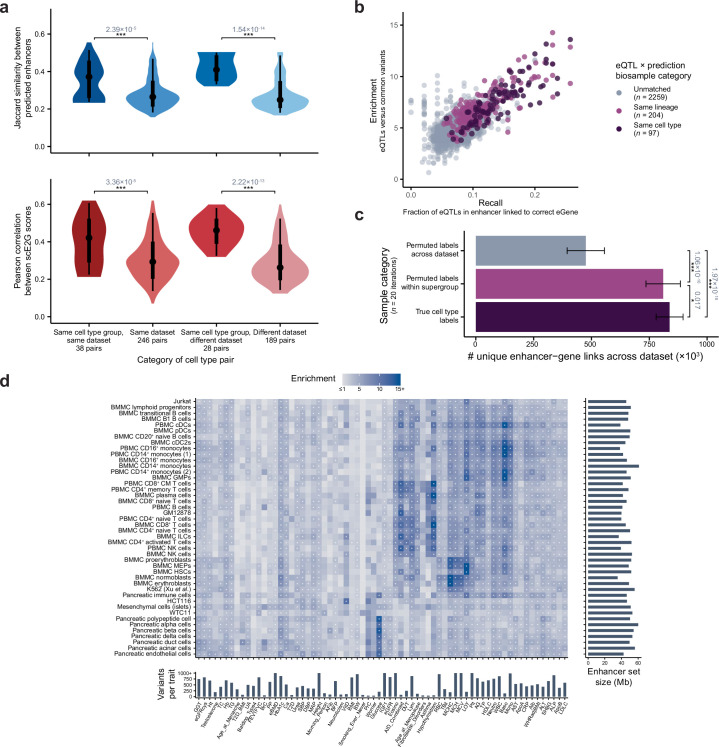
Cell-type resolution of scE2G^Multiome^ predictions. **a**, Jaccard similarity (top, blue) and Pearson correlation (bottom, red) between scE2G^Multiome^ predictions for different categories of cell type pairs for 30 PBMC and BMMC cell types: same cell type group, same dataset (*for example*, PBMC CD14^+^ monocytes and PBMC CD16^+^ monocytes); same dataset (all pairs of PBMC cell types); same cell type group, different dataset (*for example*, PBMC CD14^+^ monocytes and BMMC CD16^+^ monocytes); different dataset (all pairs of one PBMC and one BMMC cell type). Three stars: *P*_adjusted_ < 0.001 (two-sided *t*-test with Bonferroni correction, exact values in grey). See Methods, ‘Data visualization’ for definition of point interval plot elements. **b**, Enrichment and recall of eQTLs in scE2G^Multiome^ predictions of the same cell type (dark purple), same cell lineage (light purple), or unmatched biosamples (grey). Each point represents one eQTL/prediction biosample pair. **c**, Number of unique predicted enhancer-gene links by scE2G^Multiome^ across BMMCs, where size-matched cell samples were selected from true cell type labels (dark purple); cell type supergroups: B, dendritic, erythroid, myeloid, T (light purple); or the whole dataset (grey). Error bars: mean ± 95% confidence intervals across 20 iterations. Three stars: *P* < 0.001; one star: *P* < 0.05 (paired-difference one-sided *t*-test; exact values in grey). Number of unique enhancer-gene links increases by an average of 76% when samples from true cell types versus all BMMCs (*P* = 1.97 × 10^−18^) **d**, Enrichment of fine-mapped GWAS variants from 68 traits in scE2G^Multiome^ predictions in all cell types (color scale limited from 1 to 15). Horizontal margin plot: variants per trait (y-axis is capped at 1000). Vertical margin plot: base pairs predicted to be in enhancers. Stars: *P*_adjusted_ < 0.05 from a one-sided hypergeometric test for enrichment with the Bonferroni correction for multiple comparisons. Both axes are hierarchically-clustered with Ward2 method using Euclidean distance of enrichment correlation.

Several observations demonstrated that scE2G predictions captured cell-type-resolved regulatory variation:

1. Closely related cell types showed the greatest prediction similarity both within and across datasets (Fig. 5a and Extended Data Fig. 9a).

**Fig. 5:**
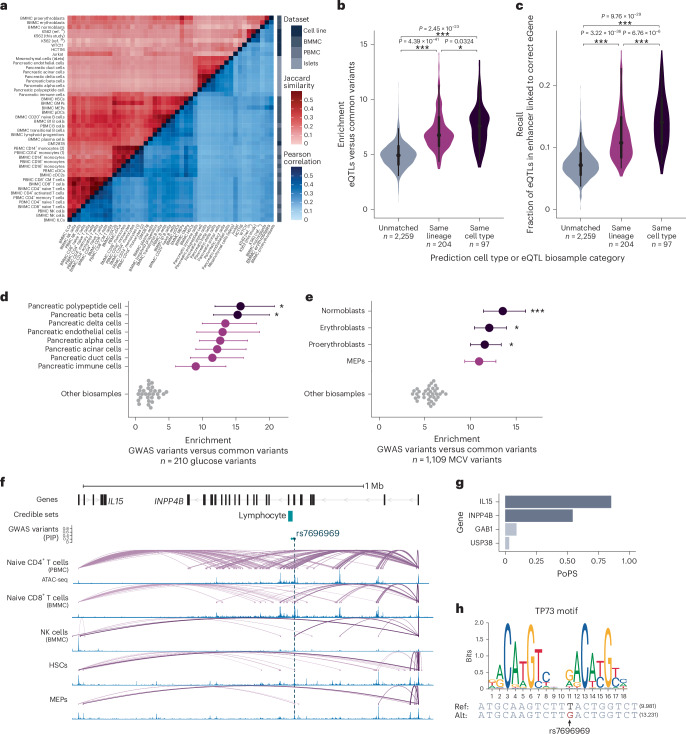
Building and interpreting maps across tissues. **a**, Heatmap showing Jaccard similarity (fraction shared links, upper left in red) and Pearson correlation (score consistency scores, lower right in blue) between scE2G^Multiome^ enhancer–gene predictions with scores above the threshold for 46 cell types. **b**, Distribution of enrichment of eQTLs in scE2G^Multiome^ predictions from the same cell type, same cell lineage or unmatched cell types (Methods). *P* values calculated from two-sided *t*-test with Bonferroni correction; ***P_adjusted_ < 0.001; *P_adjusted_ < 0.05; exact values in gray (see Methods, ‘Data visualization’ for point interval description). **c**, As **b**, but comparing distribution of recall of eQTLs in scE2G^Multiome^ predictions. **d**, Enrichment of fine-mapped GWAS variants for random blood glucose in scE2G^Multiome^ predictions across cell types. Enrichment in pancreatic polypeptide and beta cells (dark purple) is significantly higher than all non-islet cell types (gray); *all *P*_adjusted_ < 0.05 (one-sided *z*-test on delta log enrichment, Bonferroni-corrected). Error bars: 95% confidence intervals calculated using normal approximation on log-transformed enrichment ratios. **e**, As **d**, for fine-mapped GWAS variants linked to MCV. Enrichment in indicated cell types (dark purple) is significantly greater than in all other cell types (gray) except for MEPs. *all *P*_adjusted_ < 0.05; ***all *P*_adjusted_ < 0.001. **f**, scE2G^Multiome^ predictions for *IL15* and *INPP4B* and normalized ATAC in naive CD4^+^ and CD8^+^ T cells, NK cells, HSCs and MEPs at chr. 4: 141613000–142900000 (hg38). Noncoding fine-mapped GWAS variants (PIP > 10%) and credible sets for lymphocyte count are shown. Vertical dashed line: variant rs7696969 (see Methods, ‘Data visualization’ for locus plot details). **g**, PoPS ranking genes within 1 Mb of rs7696969 for lymphocyte count. **h**, Position weight matrix logo for TP73 motif (MA0861.1) aligned with sequences for reference and alternate alleles of rs7696969. Number after sequence: FIMO motif match score (https://meme-suite.org/meme/tools/fimo); *P* values calculated from log-odds score under zero-order background model with no multiple testing corrections; *P* = 3.8 × 10^−5^ and 9.23 × 10^−6^ for matches to reference allele and alternate alleles, respectively.
2. Fine-mapped eQTL variants were enriched more strongly in predictions from matched or lineage-related cell types than from unmatched cell types (Fig. 5b,c and Extended Data Fig. 9b).
3. Cell-type-resolved predictions identified 76% more unique enhancer–gene links in BMMCs than aggregate predictions (Extended Data Fig. 9c).
4. At individual loci such as *EBF1* (refs. ^55,56^) and *CEBPB* (ref. ^57^), scE2G predictions revealed the expected cell-type-relevant regulation with varying enhancer usage across related cell types (Fig. 1b,c), whereas predictions made on pseudobulk data from all cells obscured this cell-type-resolved variation (Fig. 1 and Extended Data Fig. 3).

Overall, we demonstrate that scE2G can identify many enhancer–gene links specific to individual cell types that are missed when analyzing aggregated cell populations.

### Linking GWAS variants to target genes

Having established confidence in the cell-type resolution of scE2G predictions, we applied scE2G^Multiome^ maps in 40 cell types to interpret fine-mapped GWAS variants from 17 relevant traits.

Fine-mapped GWAS variants were enriched most strongly in predicted enhancers from biologically relevant cell types (Extended Data Fig. 9d). For example, variants associated with random blood glucose were more enriched in pancreatic beta and polypeptide cell enhancers (14.6-fold average) than in non-islet enhancers (Fig. 5d; both *P*_adjusted_ < 0.05), whereas variants associated with mean corpuscular volume (MCV) were most enriched in erythroid lineage enhancers (12.0-fold average; all *P*_adjusted_ < 0.05; Fig. 5e). Stratified linkage disequilibrium score regression (S-LDSC; https://github.com/bulik/ldsc)^58^ confirmed similar trends for enrichment of genome-wide heritability (Supplementary Note 5 and Supplementary Table 8).

We used scE2G^Multiome^ to nominate causal genes and cell types for 1,892 noncoding credible sets comprising 3,686 variants (PIP > 10%; Methods). scE2G nominated 1,450 variants linked to 1,351 genes across 2,840 unique predictions linking credible sets, genes and cell types (Supplementary Table 9). Notably, 458 credible sets linked most strongly to genes beyond the nearest TSS.

**Extended Data Fig. 10:**
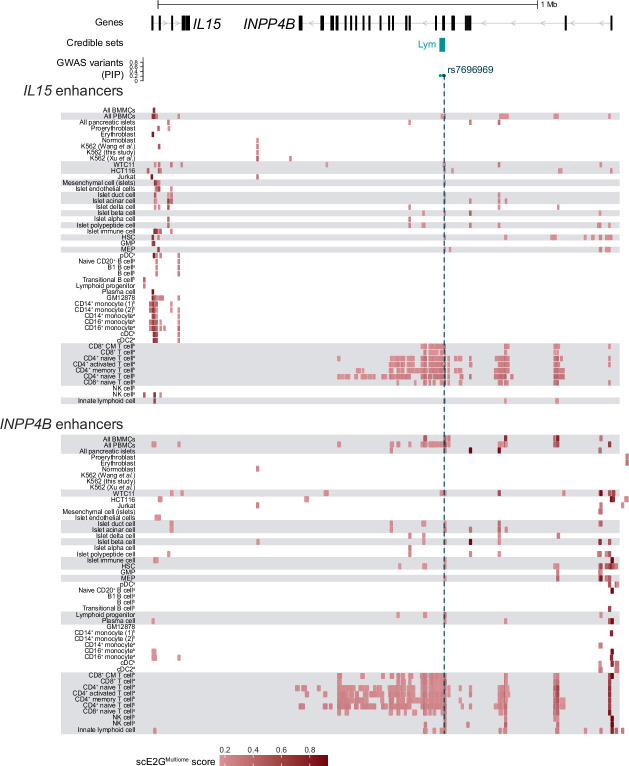
GWAS variant interpretation: enhancer predictions across cell types at selected locus. scE2G^Multiome^ predictions for *IL15* (top) and *INPP4B* (bottom) across cell types at chr4:141,613,000-142,900,000 (hg38), the locus shown in Fig. 5f. Heatmaps show enhancer predictions with a score above the threshold for each gene; element color indicates the scE2G score magnitude. An asterisk or dagger after the cell type denotes source from BMMC or PBMC datasets, respectively. Noncoding fine-mapped GWAS variants with PIP > 10% and credible sets for lymphocyte count are shown; vertical dashed line indicates variant rs7696969. All cell types with predictions overlapping the variant are highlighted in grey. See Methods, ‘Data visualization’ locus plot details.

At the 4q31.21 locus, scE2G generated variant-to-function hypotheses that illustrated its ability to make long-range predictions. The variant rs7696969, associated with lymphocyte count (fine-mapping PIP = 22%) and platelet distribution width (lead variant; *P* = 5.4 × 10^−48^) (http://www.nealelab.is/uk-biobank/), lies in an intron of *INPP4B* and overlaps an enhancer predicted to regulate both *INPP4B* and the nearby gene *IL15*. These enhancers were predicted to be active in many lymphoid cell types, most strongly in natural killer (NK) and T cells, and more weakly in hematopoietic stem cells (HSCs) and megakaryocyte-erythroid progenitors (Fig. 5f and Extended Data Fig. 10). Both promoters are located far from the variant (441 kb for *INPP4B*, 769 kb for *IL15*), and both genes are plausible regulators of lymphocyte count. *INPP4B* encodes a phosphatase that modulates the AKT pathway, and its deletion in hematopoietic cells affects B cell populations in mice^59^. *IL15* encodes a cytokine required for NK and memory CD8^+^ T cell development^60^. An independent GWAS gene prioritization method, PoPS^52^, also prioritizes *IL15* and *INPP4B* as the top two genes in the locus (Fig. 5g). In addition, both IL15 and INPP4B have been shown to mediate erythropoietin-induced erythropoiesis in murine hematopoietic stem cells^61,62^. Supporting its regulatory role, rs7696969 is a fine-mapped eQTL for *INPP4B* (PIP = 66.6%) and a chromatin accessibility QTL in lymphoblastoid cell lines^48,63^. It also overlaps transcription factor footprints for TP73 in several T cell types and alters a TP73 motif (Fig. 5h). Together these data indicate that rs7696969 may affect several blood traits through cell-type-specific, long-range regulation of *INPP4B* and *IL15*. This example illustrates the utility of scE2G in nominating causal genes and cell types affected by GWAS variants.

## Discussion

Here we present scE2G, a family of models to predict enhancer–gene regulatory interactions from single-cell data. By enabling accurate inference across diverse cell types and states, scE2G facilitates building regulatory maps to understand gene regulation, developmental biology and disease-associated genetic variation.

scE2G models use a distinct architecture from previous single-cell models that improves both accuracy and stability. Although most existing models rely on either element–gene correlation, regression or combinations of accessibility and distance, scE2G is a supervised classifier trained directly on CRISPR data. This approach allows the integration of features, including the ABC score, element–gene correlation and promoter class, that outperforms any individual feature or model. Mechanistically, the ABC score captures contributions of activity and 3D contacts (Supplementary Fig. 8), as in previous work^12,19^, the element–gene correlation may capture additional variance in these properties and promoter class may reflect how promoters of housekeeping genes are less responsive to distal enhancers^64^. The scE2G models extend the ability to predict enhancer–gene regulatory interactions to the broad range of cell types accessible through single-cell assays, providing an advantage versus bulk-based models in cell-type resolution and outperforming existing single-cell methods in predictive accuracy.

A key strategy of scE2G is the use of within-cell-type element–gene correlations, which appear to reflect stochastic temporal relationships between element accessibility and gene expression across single cells. In particular, we show that correlations calculated across single cells within a given cell type outperform those computed across cell types or within a cell type across meta-cells. This finding highlights the advantages of single-cell data over meta-cell data, and of a paired multiomic assay over separate ATAC and RNA assays, providing guidance for future experimental design and method development.

scE2G is robust to variation in sequencing depth and cell count, in part by integrating the element–gene correlation feature, which is sensitive to sequencing depth, with the ABC score, which is less sensitive. These design choices and accompanying guidelines will enable enhancer mapping across datasets at scale.

Our study applies and extends a benchmarking framework we previously developed with the ENCODE consortium^19^. This approach combines three orthogonal genetic perturbation datasets, each with its own set of strengths and limitations, to test the generalizability of models across cell types and prediction tasks. Here we extended this framework by curating additional eQTL datasets and GWAS traits with matched single-cell datasets, generating predictions using ten existing single-cell enhancer prediction models, and providing comprehensive benchmarking of single-cell element-to-gene linking models. We provide these benchmarking pipelines and curated datasets to facilitate the development and consistent evaluation of future models (‘Code availability’).

scE2G maps will improve interpretation of noncoding variants associated with complex traits by enabling increased coverage of cell types and states within tissues, as reflected by increased enrichment and recall of variant–gene associations at higher cell type resolution (Fig. 5b,c). Here we illustrate this by interpreting variants associated with metabolic and blood cell traits, identifying variant–gene links to *IL15* and *INPP4B*. When linking variants to target genes, we note the importance of combining enhancer–gene predictions with orthogonal information about gene function, as in previous work^19,52,65,66^. For example, combining scE2G with PoPS^52^ increased precision at identifying likely causal genes (Fig. 2h,i).

Taken together, our results indicate that scE2G is best suited for analyzing the diverse cell types and states accessible through single-cell assays, particularly single-cell multiomics. For cell types represented by at least 100 cells, with a minimum of 2 million ATAC fragments and 1 million RNA UMIs, scE2G enables accurate prediction of enhancer–gene regulatory interactions while calibrating appropriately for data quality and depth. For the subset of cell types in which bulk ATAC-seq or DNase-seq is available, and where single-cell data is not available or does not meet these requirements, the ENCODE-rE2G model achieves similar performance, and can be improved further by incorporating additional bulk datasets such as H3K27ac or high-resolution Hi-C.

We note several limitations and opportunities for further development. First, scE2G is designed to predict the effects of distal elements that act as enhancers, and not the effects of elements that act through other mechanisms, such as topological elements that bind CTCF. Second, the CRISPR perturbation data upon which scE2G is trained contains only 466 positive element–gene links and is limited to K562 cells and, despite outperforming other models on unseen cell types (Fig. 2e–i and Extended Data Fig. 4e,f), the performance on other cell types is modest. Expanding the CRISPR training dataset by characterizing true regulatory interactions with large-scale perturbation studies across cell types should enable further improvements to scE2G. Future studies may also explore jointly training models on CRISPR data and eQTL data. For example, a recent study^67^ developed pgBoost—a model trained to predict eQTL variant–gene links based on genomic distance and scores from other correlation-based element–gene models. Third, whereas scE2G models achieve performance similar to that of models based on bulk DNase-seq data (ENCODE-rE2G^19^), we have observed that even more accurate models can be achieved by incorporating additional bulk epigenomic assays such as cell-type-specific Hi-C data or H3K27ac chromatin immunoprecipitation sequencing data^19^. Thus, we have not yet reached the maximum performance achievable in this CRISPR dataset. Fourth, although we leverage eQTL and GWAS data for orthogonal benchmarking of scE2G, optimal performance at these prediction tasks will require additional models that consider information beyond element–gene links, for example, by considering the effects of single-nucleotide variants on elements and the effects of genes on phenotypes.

In summary, this study presents a state-of-the-art model to predict enhancer–gene regulatory interactions from single-cell data and demonstrates its utility in interpreting the function of genetic variation. The scE2G models can be applied to datasets of varying sizes and sequencing depths, and can build comprehensive and accurate maps of enhancer–gene regulatory interactions across thousands of cell types and states in the human body. For example, in parallel work we have applied scE2G to single-cell atlases of the human coronary arteries^68^ and human fetal heart^69^, identifying and validating enhancers containing variants linked to cardiovascular diseases. By creating these maps of enhancer landscapes, we expect scE2G to provide the foundation for a widely useful approach to study many common diseases and complex traits.

## Methods

This study involved computational analysis of publicly available datasets and did not involve human participants, animal subjects or human biological samples. Therefore, ethical approval was not required for this research. Refer to the Supplementary Methods for more detailed descriptions of each Methods section.

### Genome build

All coordinates in the human genome are reported in GRCh38.

### Gene reference files for scE2G and benchmarking analyses

A consistent set of gene and promoter coordinates were used across all analyses, defined as follows: GENCODE v29 for gene type annotation, retaining protein-coding, processed transcript and lincRNA genes with one promoter per gene (500 bp around RefSeq TSS with most coding isoforms). Filtering for compatibility with GENCODE v43 mapped to 20,531 unique genes.

### Collecting and preprocessing of 10x Multiome data

We analyzed publicly available 10x Multiome (ATAC + GEX) datasets in K562 (refs. ^39,71^), PBMC, BMMC^72^, GM12878 (ref. ^73^) and pancreatic islets^74^, and newly generated 10x Multiome datasets in K562 (acquired from authors of a previous study^19^), Jurkat (acquired from ATCC, accession TIB-152), HCT116 (acquired from ATCC, accession CCL-247) and WTC11 (acquired as a gift from I. Karakikes) (Supplementary Table 10). Reference genomes (GRCh38 for human samples, mm10 for mouse samples) and annotations were processed with cellranger-arc mkref (v2.0.2). Raw FASTQ, BAM and SRA files were downloaded and converted as needed, following 10x Genomics guidelines. Standard cellranger-arc pipelines were run to obtain count matrices and fragments. For the WTC11 dataset, inducible pluripotent stem cells with doxycycline-inducible KRAB–dCas9 were differentiated toward hepatocyte progenitors and profiled at several stages using the 10x Multiome platform.

### Quality control for 10x Multiome data

We selected high-quality cells using the following metrics, with thresholds set per dataset (Supplementary Table 11): RNA UMIs, ATAC fragments, nucleosome signal, TSS enrichment and percent mitochondrial transcripts. To distinguish human K562 cells from mouse cells in dataset of Wang and colleagues^71^, we selected cells with >95% reads mapped to the human genome for both scRNA-seq and scATAC-seq. For the K562 (this study), Jurkat and HCT116 datasets, we processed high quality cells using standard Signac and Seurat workflows and subsetted to relevant cell types after demultiplexing with the deMULTIplex2 (ref. ^75^) R package (v1.0.1).

### Cell-type annotation

We annotated cell types using reference datasets and marker gene expression. For BMMCs, we used cell type information from the published ref. ^72^. For PBMCs, we integrated cell type information from the Healthy Hematopoiesis scRNA-seq dataset using Harmony^76^ (v1.2.0) followed by clustering with Seurat (v5.0.3)^77^. For pancreatic islets, we conducted scRNA-seq analysis and clustering with Seurat (v5.0.3)^77^ and annotated cell types based on marker gene expression. For the WTC11 dataset, we identified the iPSC state using marker gene expression.

### Preparing input files for scE2G

For each cell type, we exported (1) an RNA count matrix (rows corresponding to genes, columns corresponding to cells; first row and column contain cell names and gene names, respectively) and (2) an ATAC fragment file with corresponding *.tbi index. Example files and specifications are available in the scE2G repository.

### Kendall correlation

We applied a nonparametric measurement—the Kendall correlation (also known as Kendall rank correlation coefficient and Kendall’s τ coefficient)^78^—to quantify the correlation between element accessibility (binarized values) and gene expression (continuous values). We extracted MACS2 (v.2.2.9)^79^ peaks that overlap with candidate elements defined in the ABC pipeline and generated the ATAC count matrix using the Signac::FeatureMatrix function. We binarized the ATAC fragment count matrix using R Signac::BinarizeCounts function, and normalized the RNA UMI count matrix using R Seurat::NormalizeData function. We computed the Kendall correlation τ_b_ between binarized accessibility and normalized expression for each candidate element–gene pair using a custom fast algorithm developed for this study.

### ARC-E2G score

According to our CRISPR benchmarking results, the ABC model and the Kendall correlation each performed best as single features dependent and independent of 3D contact or distance, respectively (Fig. 2b,c). However, the performance of the Kendall correlation, and therefore its weight in the scE2G logistic regression model, was dependent on sequencing and cell depth. To improve robustness across sequencing depths, we integrated the ABC score and Kendall correlation into a single feature: the ARC-E2G score, defined as:

$$\mathrm{ln}({\mathrm{ARC}}\mathrm{-}{\rm{E}}2{\rm{G}}\,\mathrm{score})=\mathrm{ln}(\mathrm{ABC}\,\mathrm{score})+(1/\beta )\times ({\sigma }_{\mathrm{ln}(\mathrm{ABC}\mathrm{score})}/{\sigma }_{\tau })\times \tau ,$$

where *β* is the slope from linear regression of the scaled ABC score on scaled Kendall *τ*:

$${\mathrm{ln}}{({\mathrm{ABC}}\mathrm{score})}_{\mathrm{scaled}}={\beta}\times {\tau }_{\mathrm{scaled}}+\alpha$$

with ln(ABCscore)_scaled_ and *τ*_scaled_ corresponding to the standardized ABC score and Kendall correlation, and σ_ln(ABCscore)_ and σ_*τ*_ to their respective s.d. values.

### Training and applying scE2G

We implemented a training and application pipeline for scE2G that applies the ABC^12^ codebase to define all candidate element–gene pairs, quantify activity at each element and compute the ABC score for each element–gene pair, using a pseudobulk scATAC-seq fragment file corresponding to a given cell type and estimating 3D contact using a power law relationship with genomic distance. We then annotate all element–gene pairs with additional activity-based features, annotate promoters as ubiquitously expressed using a classification obtained from previous work^64^, compute the Kendall correlation between element accessibility and gene expression and calculate the ARC-E2G score that integrates the Kendall correlation and ABC score.

We trained the scE2G models as logistic regression classifiers on the K562 CRISPR perturbation dataset, where positive element–gene pairs were defined as cases where perturbing the element had a significant and negative effect size on expression of the target gene. To evaluate the performance of the logistic regression models on the CRISPR dataset, we used chromosome-wise cross validation by holding out one chromosome, training a model on the rest and using this model to score each element–gene pair on the held-out chromosome. All performance measures for scE2G on the CRISPR training dataset report this cross-validation result (for example, Figs. 2–4, Supplementary Figs. 1 and 3–6 and Extended Data Figs. 4, 7 and 8). For scE2G^Multiome^, we then implement a gene expression filter (specified by a minimum TPM) by setting the score of all element–gene pairs where the gene is expressed below the TPM threshold to 0. We also train a model on all chromosomes that is used to apply scE2G to new cell types. Using this framework, we selected final sets of features for two models: scE2G^ATAC^, which uses just scATAC-seq data, and scE2G^Multiome^, which uses multiomic data. We define a score threshold for binarized enhancer predictions as the cross-validated score yielding 70% recall on the CRISPR benchmark (0.177 and 0.174 for scE2G^Multiome^ and scE2G^ATAC^, respectively).

### Additional versions of scE2G

To assess dependence of scE2G on sequencing depth and coverage, we randomly downsampled the K562 dataset of Xu and colleagues^39^ while varying cell number mean RNA UMI count, mean ATAC fragment count and ATAC:RNA ratio (Extended Data Fig. 7a–c). We generated and benchmarked scE2G models on each downsampled dataset (Fig. 4 and Extended Data Fig. 7). We also trained scE2G models on each downsample to examine the stability of optimal model weights across cell number and sequencing depth.

To assess scE2G models constructed using meta-cells, we constructed 90 meta-cells based on the K562 scRNA-seq data following SEACells (v0.3.3)^70^ tutorials, then generated aggregated RNA and ATAC count matrices. We then computed the Kendall correlation (meta-cell) for candidate element–gene pairs using these meta-cell definitions and retrained scE2G models with the Kendall correlation (meta-cell) and ABC score as separate features.

### Published predictors

We ran the published predictors on each 10x Multiome dataset using the same input RNA UMI count matrix and ATAC fragment count matrix, unless otherwise specified. We obtained the RNA UMI count matrix as described above for scE2G. For datasets containing only one cell type (K562, GM12878, HCT116, Jurkat and WTC11), we obtained the ATAC fragment count matrix as described above for scE2G. For datasets containing several cell types (PBMC and BMMC), we extracted MACS2 peaks that overlap with candidate enhancers defined in the ABC pipeline for each cell type, then merged peaks from all cell types. For fair benchmarking, we made predictions uniformly for peak-gene pairs with a distance to TSS less than 1 Mb. We also conducted a version of the CRISPR benchmark using the default settings of each method on the K562 dataset from Xu and colleagues^39^ (Supplementary Fig. 4). See Supplementary Methods for details on implementing each predictor.

For benchmarks of published predictors requiring predictions for individual cell types within a dataset, we subset candidate enhancer–gene pairs from the full dataset (PBMC and BMMC) to those with candidate enhancers overlapping accessible peaks and with detected genes (UMI count >0 in more than 1% of cells) in the individual cell type. For Cicero, we subset predictions based only on peak accessibility.

### CRISPR benchmark on training dataset

We used the CRISPR benchmarking pipeline developed in previous work^19^ to compare predictive models to perturbation data from previous studies in which CRISPR was used to perturb candidate enhancers and effects on gene expression. To ensure a fair comparison of AUPRC values for predictors that might produce identical scores for many element–gene pairs, we calculated the AUPRC using the trapezoidal method and included the area of the rectangle formed by the left endpoint of the curve and the origin (0,0).

### Creating held-out CRISPR dataset

We collected and reprocessed data uniformly from eight CRISPR perturbation screens covering five cell types to create a held-out dataset on which to benchmark our models. These datasets included: three Perturb-seq experiments in K562 (refs. ^44–46^); two enhancer screens performed in K562 and WTC11 using direct-capture targeted Perturb-seq^47^ and element–gene pairs tested with CRISPRi-FlowFISH in K562 (ref. ^43^), HCT116 (ref. ^42^), GM12878 (ref. ^41^) and Jurkat cells^41^. See Supplementary Methods for data processing details.

### Annotating elements with chromatin categories

We annotated the tested elements in training and held-out CRISPR datasets using H3K27ac, H3K27me3 and CTCF epigenetic datasets from ENCODE to characterize the chromatin states of the tested and positive element–gene pairs. See the Supplementary Methods for details of the classifications.

### Calculating direct and indirect effect rates

To distinguish direct *cis*-acting from indirect *trans*-acting effects in CRISPR perturbation data, we estimated indirect effect rates empirically by testing distal enhancer perturbations for interchromosomal *trans*-acting effects. We calculated direct effect rates for each dataset by subtracting the indirect effect rate from the observed positive hit rate for pairs stratified into 50-kb bins based on distance to TSS; then fit a model to the average direct effect rate as a function of distance to predict the expected direct effects rate for all CRISPR element–gene pairs.

We then estimated the probability that an observed effect was direct rather than indirect as a function of distance to TSS, *d*, as:

$${P}_{\mathrm{direct}}\left(d\right)=\frac{{r}_{\mathrm{direct}}\left(d\right)}{{r}_{\mathrm{direct}}\left(d\right)+{r}_{\mathrm{indirect}}},$$

where *r*_direct_ and *r*_indirect_ are the direct and indirect effect hit rates, respectively.

### CRISPR benchmark on held-out dataset

To create the combined benchmarking dataset from held-out datasets described in above, we combined all of the processed element–gene pairs, resolved any duplicated element–gene pairs tested in the same cell type and removed pairs that were present in the training dataset. To obtain a clean set of negatives, we retained non-significant element–gene pairs that were tested with sufficient power to detect a change in gene expression. To obtain a clean set of positives likely to represent real enhancer interactions, we applied filters based on effect size and chromatin state at the tested element. To take into account the uncertainty of indirect CRISPR perturbation effects when benchmarking enhancer–gene prediction models, we computed precision–recall curves where each CRISPR element–gene pair was weighted by its probability to be a direct effect.

### eQTL benchmark

We adapted our previously developed eQTL benchmarking pipeline to assess predictive models for their ability to (1) identify enhancers enriched for fine-mapped eQTL variants versus common variants and (2) correctly link enhancers overlapping variants to eQTL target genes. We analyzed variants from the eQTL catalog V7 fine-mapped with the Sum of Single Effects (SuSiE) model^48^, retaining those with posterior inclusion probability over 50% and located in distal noncoding regions. We grouped variants based on their biosample of origin at ‘fine-grained’ and ‘coarse-grained’ resolutions. We then assigned each cell type with predicted enhancer–gene links to matched eQTL biosamples for benchmarking (Supplementary Table 4). We computed aggregate metrics by summing variant counts across matched cell types.

### GWAS benchmark

Using an approach taken in previous work^19,41^, we developed a GWAS benchmarking pipeline to evaluate predictive models for their ability to (1) enrich for fine-mapped GWAS variants and (2) link GWAS signals to causal genes. We used GWAS data for 94 traits from the UK Biobank fine-mapped with SuSiE by the Finucane Laboratory, and retained variants located in distal noncoding regions with a fine-mapping posterior inclusion probability greater than 10%. To evaluate models at linking GWAS signals to causal genes, we used an evaluation set developed in previous work^52^. Briefly, probable causal genes for each trait were defined as those containing a protein-coding, fine-mapped variant with a posterior inclusion probability greater than 50%, and each causal gene was linked to all independent noncoding credible sets for the same trait located within 500 kb.

For the benchmarking evaluation, we assigned sets of prediction cell types to sets of GWAS traits expected to be relevant to those biosamples (Supplementary Table 5). To evaluate models at identifying causal genes in combination with the PoPS, we defined credible set-gene predictions for a model ∩ PoPS as each credible set linked to any gene that ranked in both the top two based on model score and the top two based on PoPS score.

### Analyzing cell-type resolution of scE2G predictions

In all analyses of cell-type resolution, we defined a ‘shared prediction’ across cell types as enhancer–gene links with the same gene and at least 50% overlap of each enhancer width.

To determine whether scE2G makes more enhancer–gene links for more fine-grained cell type while controlling for the number and size of the input cell clusters, we considered 15 main cell types from BMMCs that comprise five cell type ‘supergroups.’ We applied scE2G^Multiome^ to 15 random samples of cells, size-proportional to the true cell type clusters, selected from: (1) all BMMCs, (2) the respective cell type supergroup or (3) the exact cell type. We then calculated the total number of unique enhancers identified using each sampling method. We repeated the sampling and prediction process for 20 iterations.

### Applying scE2G to prioritize causal genes and cell types for GWAS loci

We estimated the enrichment of trait heritability in predicted enhancers using S-LDSC (v1.0.1 (refs. ^58,80^)). We considered all nonpromoter enhancers predicted by scE2G^Multiome^ above the threshold. We prepared GWAS summary statistics for 104 traits from the UK Biobank^49^ obtained from the Finucane Laboratory. We performed S-LDSC using the scE2G^Multiome^ enhancers along with the baseline model (v2.2).

To apply scE2G systematically to prioritize causal genes and cell types for GWAS signals, we considered cell type and trait pairs for which variants were enriched significantly by at least fivefold in predicted enhancers for that cell type. We identified all credible set, cell type and gene combinations for which the credible set has noncoding variants located in a predicted scE2G^Multiome^ enhancer linked to that gene in that cell type, and retained the two predicted genes per credible set supported by the highest scE2G scores. The full gene prioritization results can be found in Supplementary Table 9.

We quantified the effect of a variant on a given transcription factor motif using motifs from JASPAR^81,82^ and match scores computed with FIMO (v5.5.9; https://meme-suite.org/meme/doc/fimo.html).

### Data visualization

#### Box plots

Box plots are defined as follows: middle line/point, lower hinge and upper hinge indicate the median, first and third quartiles, respectively. The interquartile range (IQR) is defined as the distance between the first and third quartiles. The whiskers extend from the corresponding hinge to the most distant value no further than 1.5× IQR from the hinge. Data beyond the end of the whiskers are outlying points and are plotted individually, unless box plots are shown on top of all datapoints or data distribution.

#### Point intervals

Histograms and violin plots containing point interval plots were made with ggdist (v3.3.3; https://mjskay.github.io/ggdist/index.html). The middle point corresponds to the median; the thick line to the quantile interval of the center 66% of data and the thin line to the quantile interval of the center 95% of data.

#### Locus plots

Locus plots were plotted with the GViz (v1.46.1)^83^ and GenomicInteractions (v1.36.0)^84^ packages. Arcs and heatmaps show predictions above the model threshold. Arc widths are scaled based on log_10_-transformed scE2G scores. Arc and heatmap element color are scaled to the scE2G score magnitude. Heatmap rows are ordered by similarity of genome-wide predictions across cell types (Supplementary Methods 19), with predictions for the combined PBMC, BMMC and pancreatic islet datasets appended at the top. The pseudobulk ATAC-seq signal tracks are plotted from the normalized bigWig file generated in the scE2G pipeline.

### Statistics and reproducibility

No statistical method was used to predetermine sample size for computational analyses. Sample sizes for CRISPR benchmark datasets were determined by the availability of published perturbation experiments. No data were excluded from the analyses except where explicitly stated (for example, quality control filtering of single cells based on standard metrics in Supplementary Table 11, retention of high-quality element–gene pairs in CRISPR datasets as described above). Randomization was used to downsample multiome datasets as described above. Blinding is not applicable to this computational study. Model performance was evaluated using cross-validation (chromosome-wise hold-out for CRISPR training data) and independent held-out datasets (CRISPR, eQTL and GWAS benchmarks in distinct cell types). All computational analyses are reproducible using the code and parameters described in Methods and Supplementary Methods, with the data and software described in the ‘Data availability’ and ‘Code availability’ sections.

### Reporting summary

Further information on research design is available in the Nature Portfolio Reporting Summary linked to this article.

## Online content

Any methods, additional references, Nature Portfolio reporting summaries, source data, extended data, supplementary information, acknowledgements, peer review information; details of author contributions and competing interests; and statements of data and code availability are available at 10.1038/s41588-026-02695-8.

## Supplementary information

Supplementary Information Supplementary Figs. 1–8, Notes 1–5 and 4 figures associated with Notes, descriptions of Tables 1–11, Methods and references.

Reporting Summary

Supplementary Tables 1–11 Supplementary Tables 1–11.

## Data Availability

The locations of the scE2G^Multiome^ prediction files are listed in Supplementary Table 6. The data sources and quality control thresholds used for each 10x Multiome sample are listed in Supplementary Tables 10 and 11, respectively. The K562 CRISPR perturbation data used for model training and benchmarking are derived from data published in refs. ^12,37,38^, and are available via GitHub at https://github.com/EngreitzLab/CRISPR_comparison/blob/main/resources/crispr_data/EPCrisprBenchmark_combined_data.training_K562.GRCh38.tsv.gz. The held-out CRISPR perturbation data are derived from data published in refs. ^41–47^ and are available via Github at https://github.com/EngreitzLab/CRISPR_comparison/blob/dev/resources/crispr_data/EPCrisprBenchmark_combined_data.heldout_5_cell_types.GRCh38.tsv.gz. The fine-mapped eQTL benchmarking set was obtained from the eQTL catalog, v7 (https://www.ebi.ac.uk/eqtl/) as described in Methods. The UK Biobank fine-mapped GWAS variant benchmarking set was obtained from https://www.finucanelab.org/data. The K562 nuclei SLIC-CAGE data are derived from data published in ref. ^85^ and are available at https://www.ncbi.nlm.nih.gov/geo/query/acc.cgi?acc=GSE306952.

## Code availability

10x Multiome data were preprocessed using cellranger-arc (v2.0.2) or cellranger (v6.0.0) and cellranger-atac (v2.0.0). Quality control and cell-type annotation were conducted using the deMULTIplex2 R package (v1.0.1), Harmony (v1.2.0) and/or the Seurat (v5.0.3) and Signac (v1.13.0) pipelines as detailed in Methods. We ran the following published enhancer–gene linking methods: ArchR (v1.0.2), Cicero (v1.3.9), DIRECT-NET (v0.0.1), FigR (v0.1.0), SCENT (v1.0.0), SCENIC+ (v1.0a1), Signac (v1.13.0), SnapATAC (v1.0.0) and STARE (v1.0.4). The following software packages were used for additional analyses and visualization: S-LDSC (v1.0.1); FIMO (v5.5.9); R packages ggdist (v3.3.3), GViz (v1.46.1) and GenomicInteractions (v1.36.0). The scE2G pipeline is available via GitHub at https://github.com/EngreitzLab/scE2G/tree/v1.3 and via Zenodo at 10.5281/zenodo.18320555 (ref. ^86^). The CRISPR benchmarking pipeline is available via GitHub at https://github.com/EngreitzLab/CRISPR_comparison/tree/AUPRC_trapezoid_add_left_rectangle and via Zenodo at 10.5281/zenodo.18320613 (ref. ^87^). The eQTL benchmarking pipeline is available via GitHub at https://github.com/EngreitzLab/eQTLEnrichment/tree/integrated and via Zenodo at 10.5281/zenodo.18320671 (ref. ^88^). The GWAS benchmarking pipeline is available via GitHub at https://github.com/EngreitzLab/GWAS_E2G_benchmarking and via Zenodo at 10.5281/zenodo.18320659 (ref. ^89^). Data analysis code for this study are available via GitHub at https://github.com/anderssonlab/scE2G_analysis/releases/tag/v1.2 and via Zenodo at 10.5281/zenodo.18320688 (ref. ^90^).
