# Supplementary material for: Mapping enhancer–gene regulatory interactions from single-cell data
Source: Nat Genet. 2026 Aug 3;58(8):1941–52. doi: 10.1038/s41588-026-02695-8 (PMC13447104; doi:10.1038/s41588-026-02695-8)
Supplement: Supplementary file 1 [file 41588_2026_2695_MOESM1_ESM.pdf]

nature genetics

Supplementary information https://doi.org/10.1038/s41588-026-02695-8

Mapping enhancer–gene regulatory interactions from single-cell data

In the format provided by the authors and unedited

Supplementary Information

Supplementary Figures **3**

Supplementary Figure 1: Evaluation of scE2G scores calculated with hold-one-chromosome-out cross-validation 3

Supplementary Figure 2: Data quality of single-cell multiome datasets 4

Supplementary Figure 3: CRISPR benchmarking on three different K562 multiome datasets 5

Supplementary Figure 4: CRISPR benchmarking of existing models using default settings 7

Supplementary Figure 5: Feature interpretation of scE2G^{Multiome} and scE2G^{ATAC} 8

Supplementary Figure 6: Robustness of scE2G and existing models to sequencing depth 10

Supplementary Figure 7: Properties of genome-wide scE2G predictions 12

Supplementary Figure 8: CRISPR benchmarking of scE2G compared to an activity-only baseline 13

Supplementary Notes **14**

Supplementary Note 1. Comparisons among candidate metrics for quantifying the relationship between gene expression and enhancer accessibility 14

Supplementary Figure N1: CRISPR benchmarking of candidate enhancer-gene link metrics 14

Supplementary Note 2. Summary of published predictors 16

Supplementary Note 3. Benchmarks against bulk DNase-seq ENCODE-rE2G models 18

Supplementary Figure N2: Benchmarking scE2G versus bulk models 19

Supplementary Note 4. Characteristics of CRISPR pairs affecting model performance 21

Supplementary Figure N3: scE2G performance on different types of element-gene pairs 22

Supplementary Note 5. Enrichment of heritability of GWAS traits in scE2G enhancers by S-LDSC 23

Supplementary Figure N4: S-LDSC heritability enrichment in scE2G enhancers 24

Supplementary Table Descriptions **25**

Supplementary Table 1. Published models 25

Supplementary Table 2. CRISPR benchmark pairwise comparisons 25

Supplementary Table 3. CRISPR benchmark performance intervals 25

Supplementary Table 4. eQTL benchmarking cell type pairings 25

Supplementary Table 5. GWAS benchmarking trait–cell type pairings 25

Supplementary Table 6. scE2G predictions 25

Supplementary Table 7. scE2GMultiome and scE2GATAC prediction properties 25

Supplementary Table 8. GWAS trait heritability enrichment in scE2GMultiome enhancers 25

Supplementary Table 9. Gene prioritization for noncoding GWAS variants 25

Supplementary Table 10. 10x Multiome data sources 25

Supplementary Table 11. 10x Multiome data quality control thresholds 25

Extended Data Figure Legends **26**

Supplementary Methods **26**

1. Genome build 26

2. Gene reference files for scE2G and benchmarking analyses 26

3. Collecting and preprocessing of 10x Multiome data 26

4. Quality control for 10x Multiome data 28

5. Cell-type annotation 28

6. Preparing input files for scE2G 29

7. Kendall correlation 30

8. ARC-E2G score 31
9. Training and applying scE2G 33
10. Additional versions of scE2G 36
11. Published predictors 37
12. CRISPR benchmark on training dataset 41
13. Creating held-out CRISPR dataset 41
14. Annotating elements with chromatin categories 46
15. Calculating direct and indirect effect rates 46
16. CRISPR benchmark on held-out dataset 48
17. eQTL benchmark 49
18. GWAS benchmark 52
19. Analyzing cell-type specificity of scE2G predictions 53
20. Applying scE2G to prioritize causal genes and cell types for GWAS loci 54
21. Data visualization 55
Supplementary References **57**

Supplementary Figures

Supplementary Figure 1: Evaluation of scE2G scores calculated with hold-one-chromosome-out cross-validation

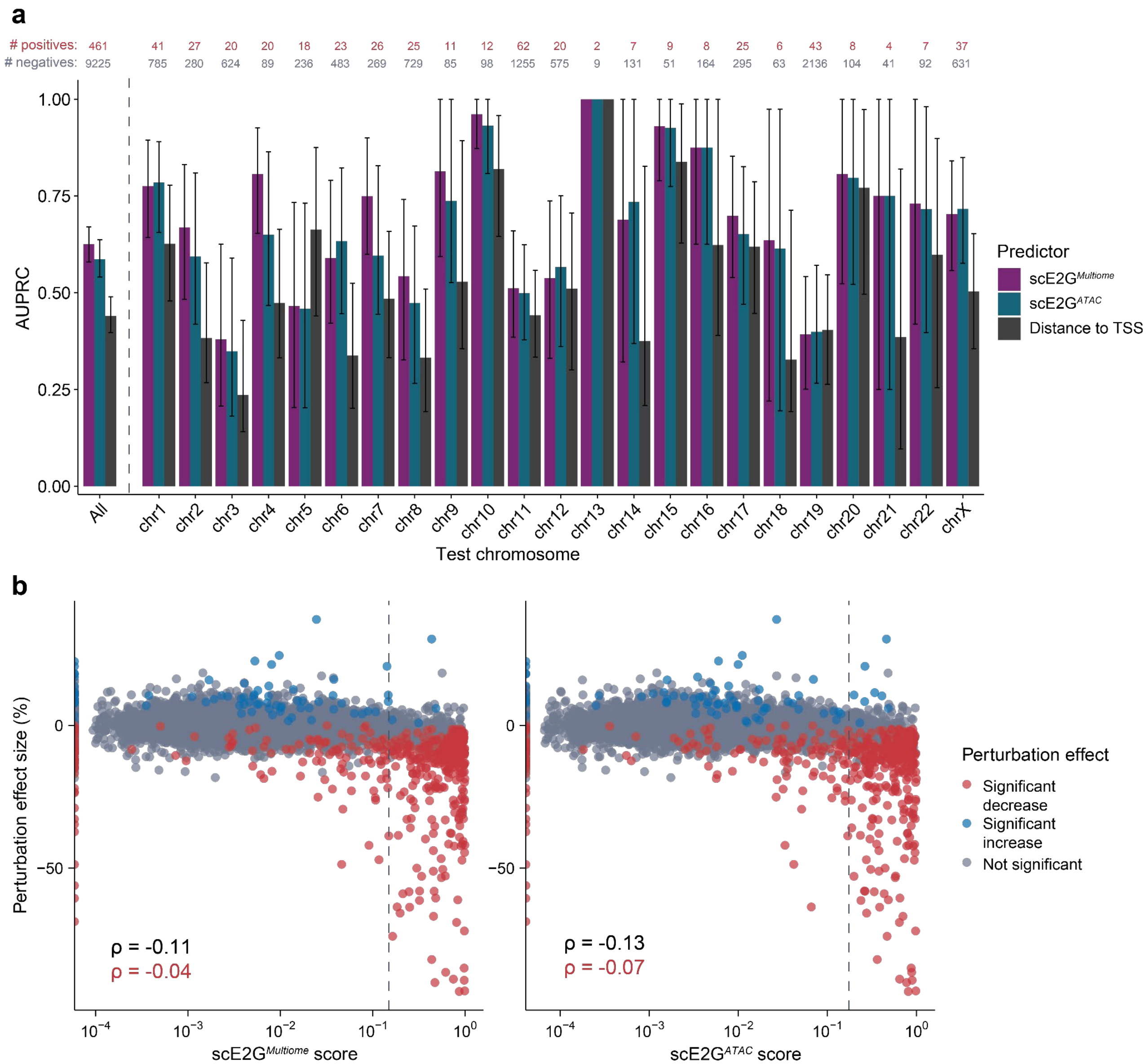

a. AUPRC of scE2G models on the held-out chromosome of the CRISPR training dataset from each fold (all distance to TSS ranges), and the AUPRC of cross-validation scores across all chromosomes. The performance of distance to TSS on each chromosome is shown as a reference. Error bars show the 95% confidence interval of AUPRC values inferred via bootstrap (1,000 iterations, stratified sampling from each class). The numbers above the plot indicate the number of positive and negative element-gene pairs on each chromosome.
b. CRISPR effect sizes on gene expression versus scE2G^{Multiome} (**left**) and scE2G^{ATAC} (**right**) scores. Each point represents one tested element-gene pair. CRISPR effect sizes represent the percent change in gene expression relative to unperturbed cells. Model threshold is indicated with a vertical dashed line. scE2G scores were computed from the K562 10x Multiome data from Xu *et al.* using models trained on hold-one-chromosome-out data. The Spearman correlation in black was computed on all CRISPR-tested pairs, and the correlation in red was computed just on pairs with a significant negative effect size. To annotate CRISPR-tested element-gene pairs with scE2G scores, we used the same approach as in the CRISPR benchmarking pipeline: each tested pair was assigned the maximum score of overlapping scE2G predictions.

Supplementary Figure 2: Data quality of single-cell multiome datasets

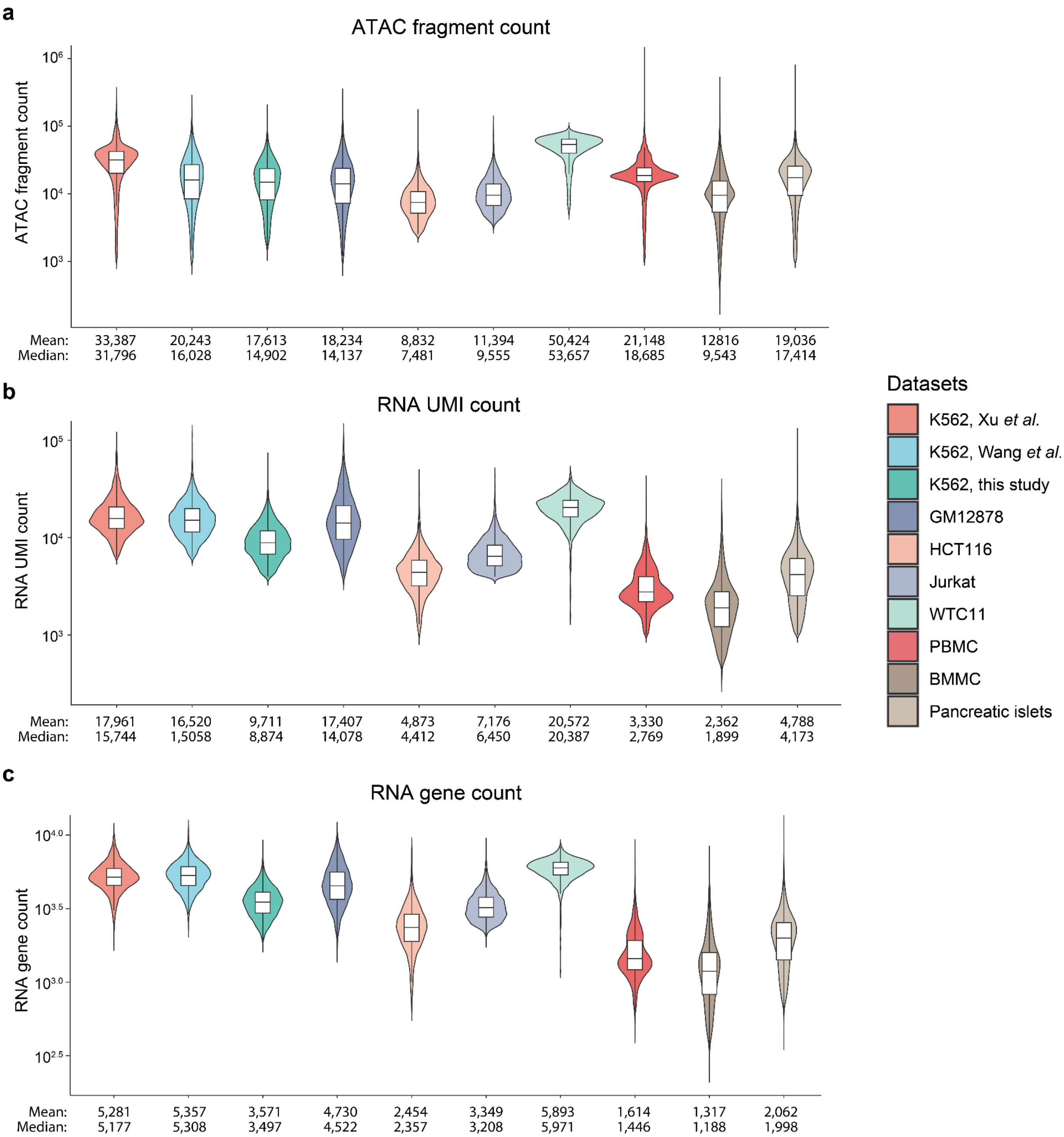

a-c. Violin plots showing the distribution of ATAC fragment counts per cell (**a**), RNA UMI counts per cell (**b**), and RNA gene counts per cell (**c**) across seven single-cell 10x Multiome datasets used in this study. Each violin density contains a box plot; see **Methods, 'Data visualization'** section for definition of box plot elements. Both mean and median values are labeled below the corresponding violin density.

Supplementary Figure 3: CRISPR benchmarking on three different K562 multiome datasets

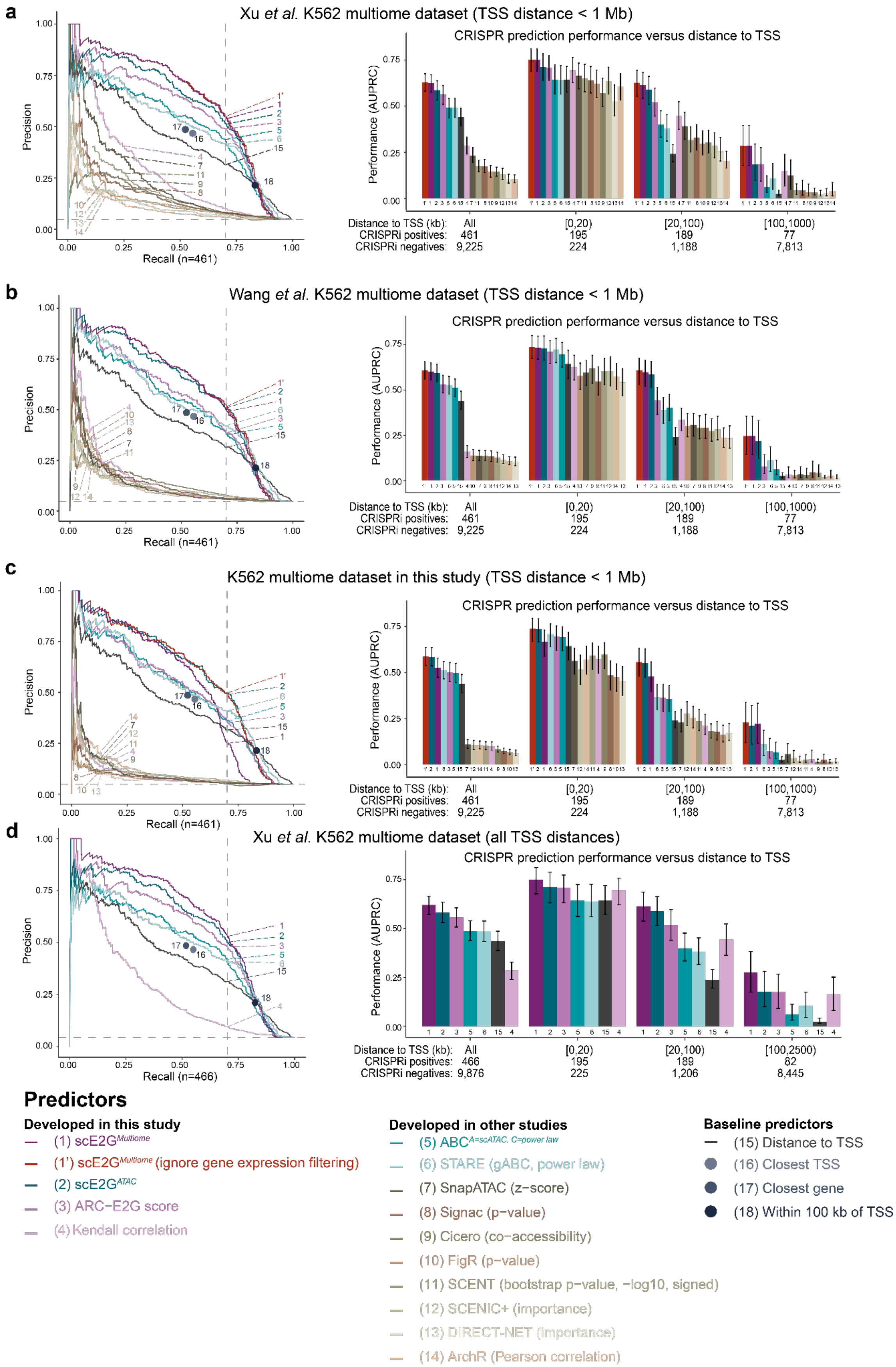

a–c. CRISPR benchmarking (TSS distance < 1Mb) of scE2G models on K562 single-cell datasets from Xu *et al*.[1] (**a**), Wang *et al*.[2] (**b**) and this study (**c**).

Left: Precision–recall curves displaying the performance of models on K562 CRISPR data, as in **Figure 2b**. The benchmarking retained only enhancer-gene pairs with a TSS distance of less than 1Mb (9,225 tested element-gene pairs, 461 positives). Curves: continuous predictors; dots: binary predictors. Dashed vertical line: 70% recall; dashed horizontal line: precision at 100% recall.

Right: CRISPR benchmarking performance of continuous predictors varies with distance to TSS. Error bars: mean +/- 95% confidence intervals inferred via bootstrap (10,000 iterations).

In the default scE2G^{Multiome} results (1), we assign predictions only to genes that are sufficiently highly expressed in a given cell type (pseudo-bulk TPM > 1, **Methods**). Here, we also compared scE2G^{Multiome} results without gene expression filtering (1’). We note that gene expression filtering primarily impacts the scE2G^{Multiome} performance on the K562 dataset in panel (**c**), indicating worse gene expression quantification than in the K562 single-cell datasets from Xu *et al.*[1] (**a**) and Wang *et al.*[2] (**b**) (See also **Supplementary Figure 2b,c**).

d. Similar to **a**, but comparing to all CRISPR-tested element-gene pairs in the dataset (9,876 tested element-gene pairs, 466 positives), including those located at distances > 1 Mb.

Supplementary Figure 4: CRISPR benchmarking of existing models using default settings

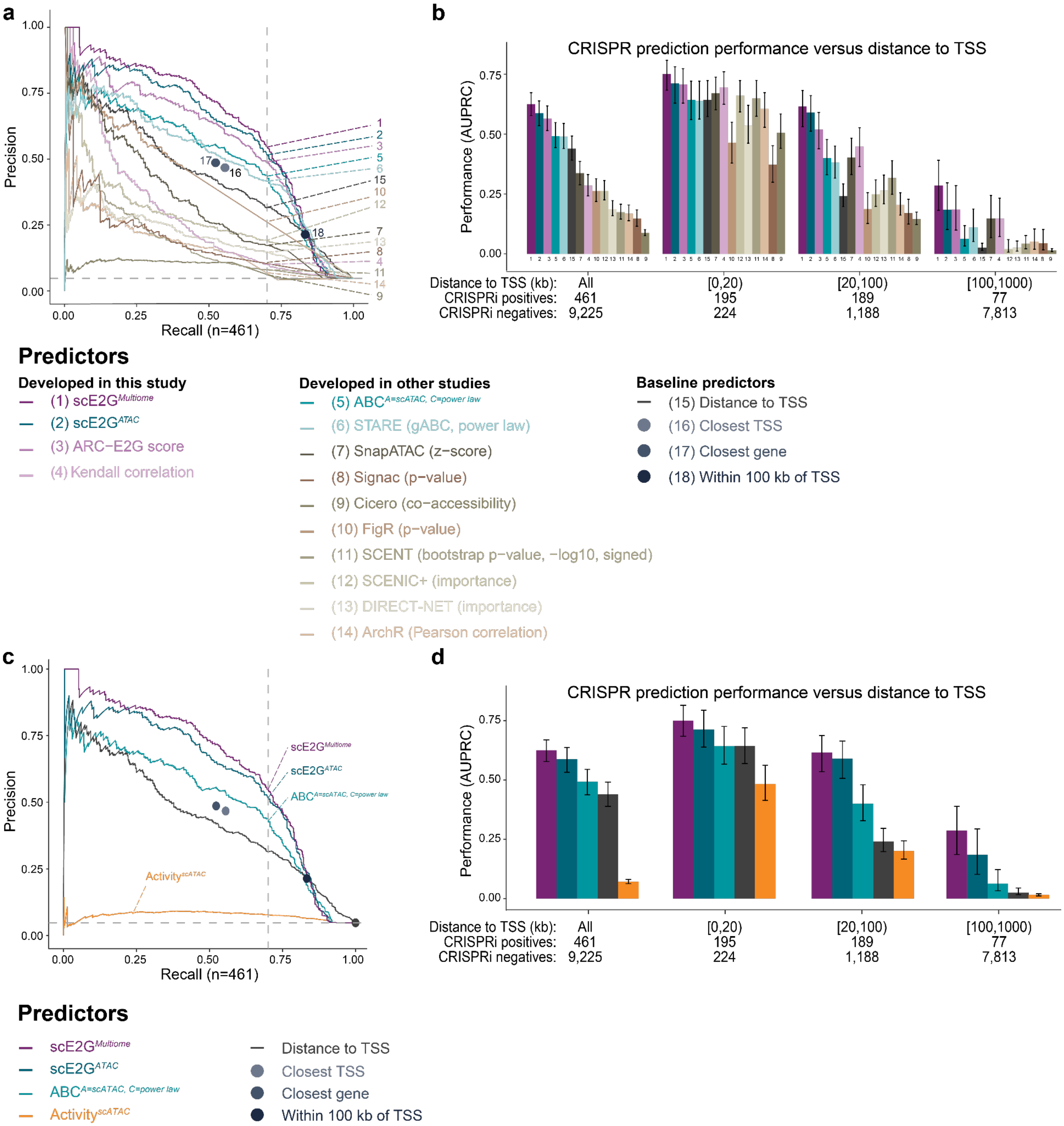

a. Precision-recall curves for the K562 CRISPR data (9,225 tested element-gene pairs, 461 positives; distance to TSS < 1 Mb). Curves: continuous predictors; dots: binary predictors. Dashed vertical line: 70% recall; dashed horizontal line: precision at 100% recall. This analysis uses the published recommended thresholds (*e.g.*, distance thresholds) for each method. In contrast, **Figure 2b** uses a consistent set of thresholds across methods.
b. AUPRC on the CRISPR benchmark versus distance to TSS. Error bars: 95% confidence intervals inferred via bootstrap (10,000 iterations).

Supplementary Figure 5: Feature interpretation of scE2G^{Multiome} and scE2G^{ATAC}

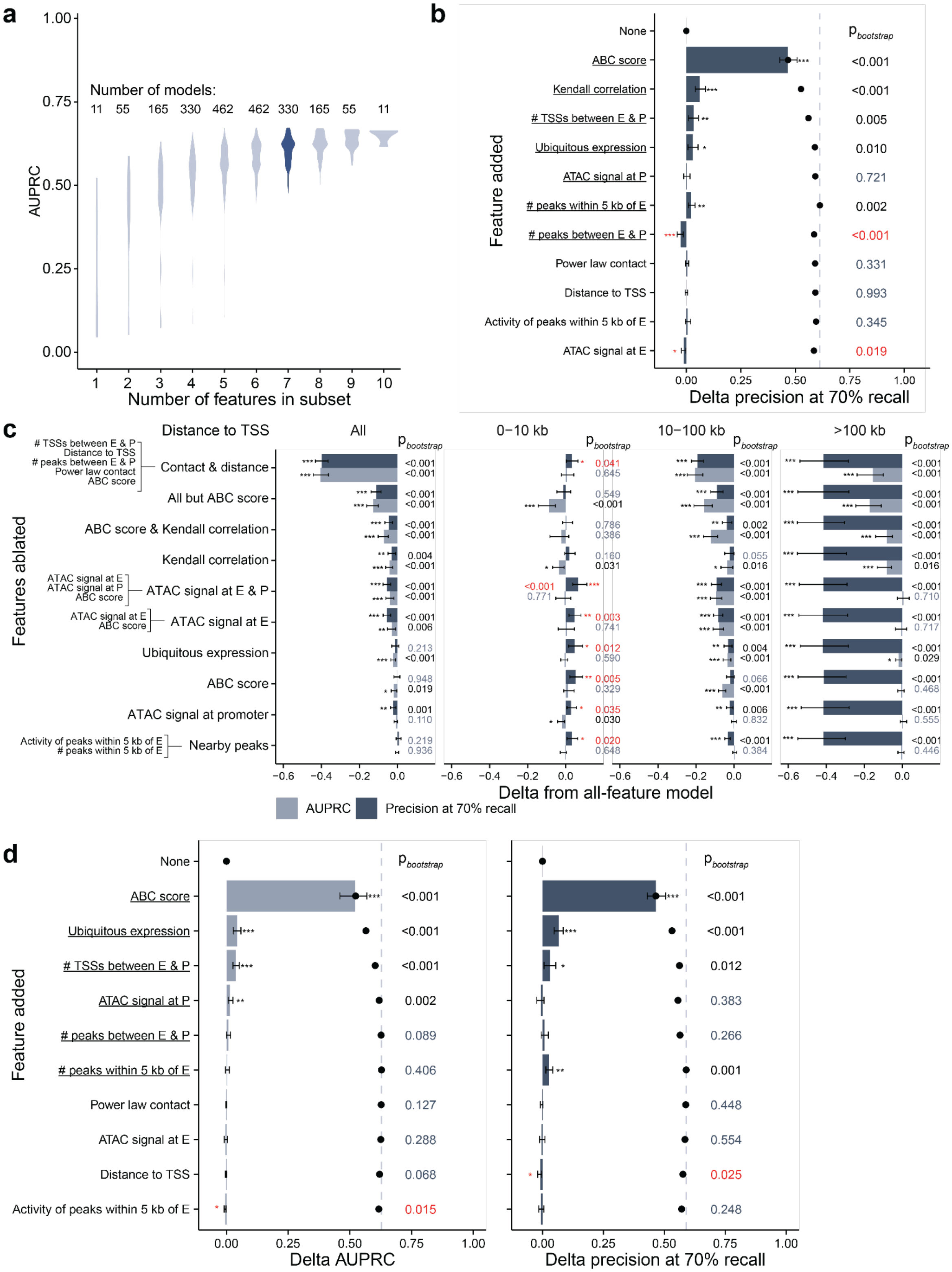

a. Violin plots showing AUPRC on the CRISPR benchmark for all possible subsets of the 11 input features (ABC score and Kendall correlation as separate features) for each subset size. Values above violin: number of models evaluated. Dark grey violin: distribution of AUPRC for 7-feature subsets, number of features used in the final scE2G^{Multiome} model with maximum AUPRC.

b. Forward sequential feature selection for scE2G^{Multiome} (ABC score and Kendall correlation as separate features), as shown in **Figure 3a**, on the CRISPR benchmark. Bars: delta precision at 70% recall from adding each feature

(order of added features determined by maximizing delta AUPRC). Dots: cumulative delta precision. Dashed line: total accumulated precision. Error bars: mean +/- 95% confidence intervals inferred via paired bootstrap (1,000 iterations). One, two, and three stars: two-sided p-value < 0.05, 0.01, and 0.001, respectively, derived from bootstrap distribution with no adjustment for multiple comparisons; black/red stars: positive/negative delta, respectively; exact p-value indicated to the right of each bar. Underlines: features used in the final model.

c. Category feature ablation for scE2G^{Multiome} (ABC score and Kendall correlation as separate features) showing delta AUPRC and precision at 70% recall on the CRISPR benchmark, with results stratified by element-gene distance to TSS. Error bars, significance indicators, and p-values as in (**b**).

d. Forward sequential feature selection for scE2G^{ATAC}, with bars representing delta AUPRC (left) and precision at 70% recall (right). Order of added features was determined by maximizing delta AUPRC. Error bars, significance indicators, p-values, and underlined features as in (**b**).

Supplementary Figure 6: Robustness of scE2G and existing models to sequencing depth

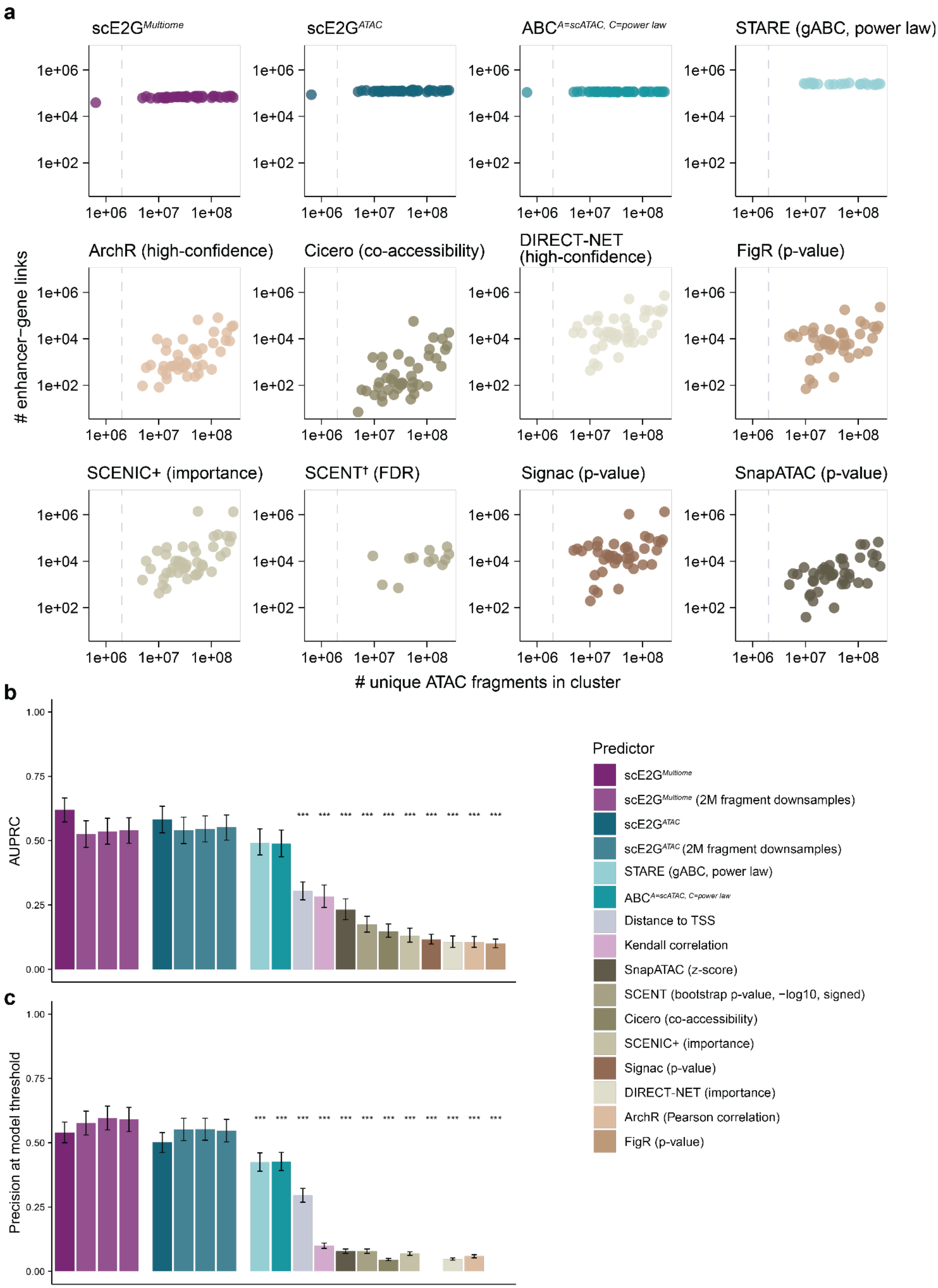

a. scE2G^{Multiome} predicts a stable number of enhancer–gene interactions (60,033–76,679) across cell types analyzed with ≥2M ATAC fragments, while non-ABC methods vary by 3–4 orders of magnitude. Plots show numbers of predicted interactions (binarized at suggested thresholds) per model versus ATAC fragments per cell type. Dots: number of predictions in one cell type. Predictions for published methods were filtered from full datasets to match detected peaks/genes (**Methods**). For SCENT, only predictions for K562 and GM12878 were generated with the recommended 50,000 bootstrap iterations; cell type supergroups predictions were generated with 25,000 iterations due to the required computational resources.

b. AUPRC on the CRISPR benchmark in K562 cells (distance to TSS < 1 Mb) comparing scE2G predictions made on the full-depth Xu *et al.* dataset, scE2G predictions made on three downsampled datasets, and other models made on the full-depth dataset. Downsample parameters: each 2M ATAC fragments, 1M RNA UMIs, with: (1) 100 cells × 20,000 ATAC, 10,000 RNA per cell; (2) 200 × 10,000/5,000; (3) 500 × 2,000/1,000. Error bars: mean +/- 95% confidence intervals inferred via bootstrapping (10,000 replicates). Stars: maximum two-sided p-value for delta performance (inferred via bootstrapping, 10,000 replicates, no correction for multiple comparisons) between the non-scE2G model applied to the full-depth dataset and the scE2G^{Multiome} and scE2G^{ATAC} on each downsample. Three stars: all p-values < 0.001; two stars: all p-values < 0.01, one star: all p-values < 0.05.

c. As (**b**), but for precision at the model threshold.

Supplementary Figure 7: Properties of genome-wide scE2G predictions

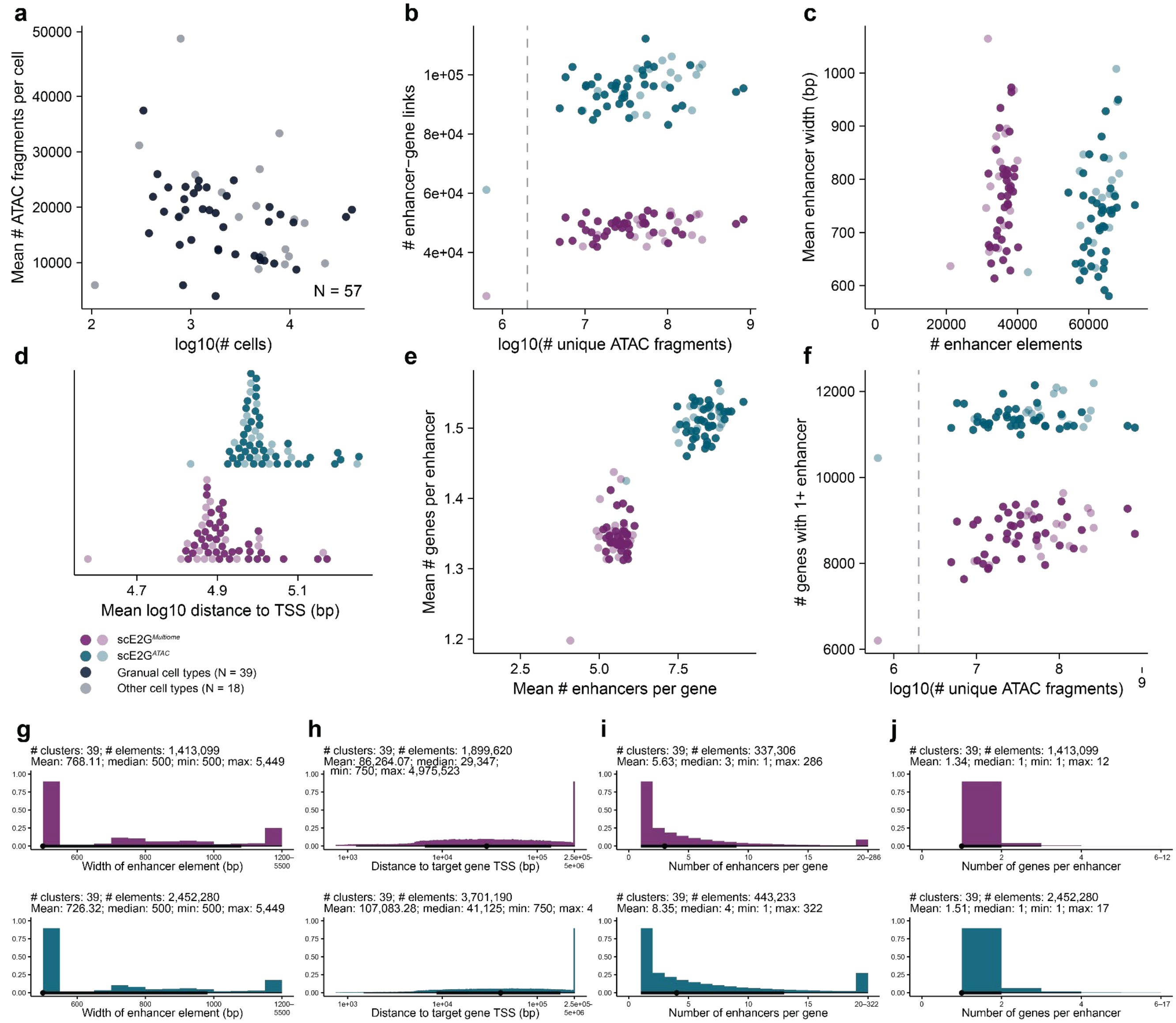

a–f: Each point represents the average value of a property for enhancer-gene predictions in one cell type or cell type supergroup, or cell line. Purple points correspond to scE2G^{Multiome} predictions (N = 57), and teal points correspond to scE2G^{ATAC} predictions (N = 57). Darker points represent the 39 cell types from the PBMC, BMMC, and pancreatic islet datasets with more than 2 million ATAC fragments. All properties were computed on binarized predictions omitting promoter elements.

a. Average number of unique ATAC fragments per cell by number of cells per cell type.

b. Number of enhancer-gene links by number of unique ATAC fragments. The dashed vertical line indicates 2 million ATAC fragments.

c. Mean enhancer width by number of unique enhancers.

d. Mean distance between enhancer and target gene transcription start site.

e. Mean number of enhancers per gene by mean number of genes per enhancer.

f. Number of genes with at least one non-promoter enhancer by number of unique ATAC fragments. The dashed vertical line indicates 2 million ATAC fragments.

g–j: Histograms showing the distribution of properties across all predictions in the 39 cell types from the PBMC, BMMC, and pancreatic islet datasets with more than 2 million ATAC fragments for scE2G^{Multiome} (purple) and scE2G^{ATAC} (teal). As in **g–j**, all properties were computed on binarized predictions omitting promoter elements. See **Methods, 'Data visualization'** section for definition of point interval elements below each histogram.

Supplementary Figure 8: CRISPR benchmarking of scE2G compared to an activity-only baseline

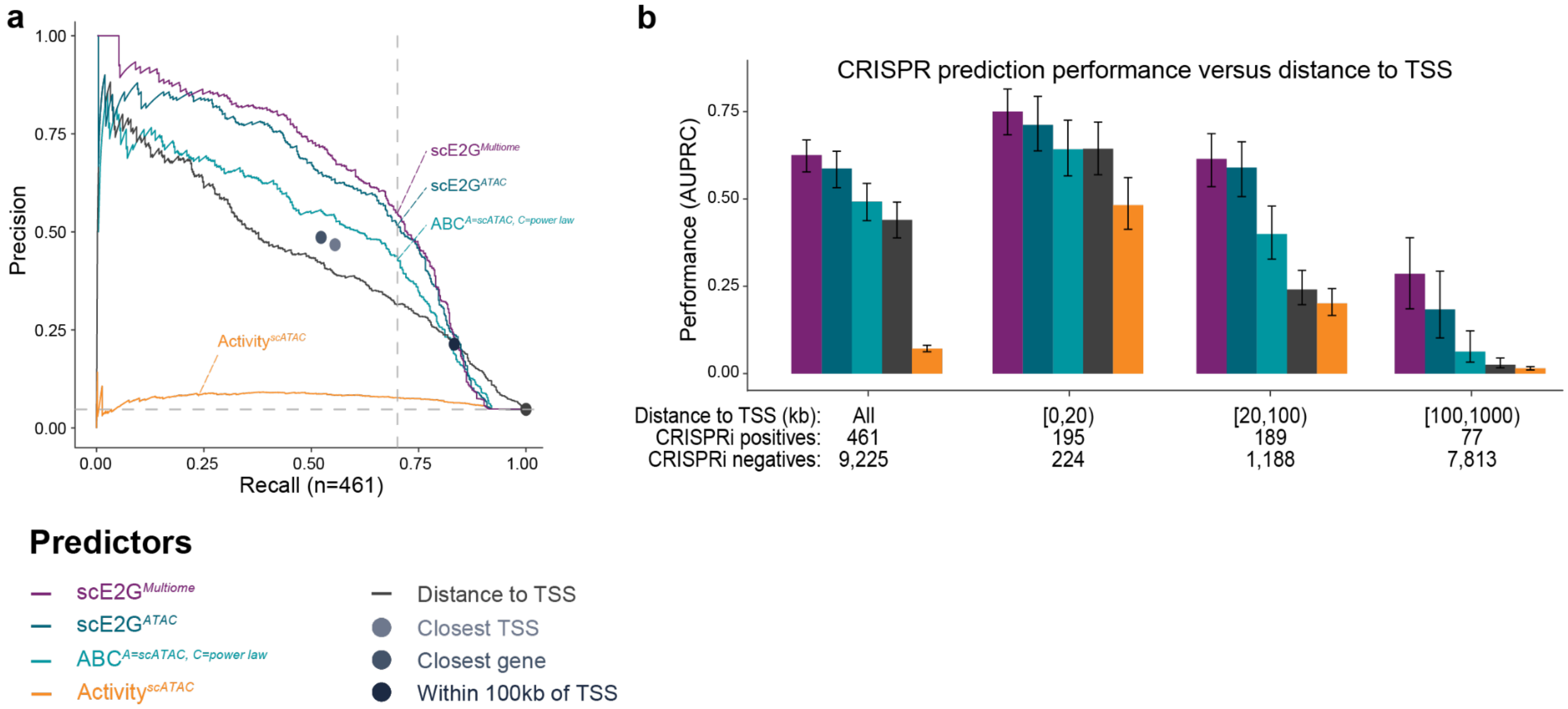

a. Precision-recall curves on K562 CRISPR data (9,225 tested element-gene pairs, 461 positives; distance to TSS < 1 Mb) for scE2G, ABC, and activity^{scATAC} (reads per million estimated from pseudo-bulk scATAC-seq data). Curves: continuous predictors; dots: binary predictors. Dashed vertical line: 70% recall; dashed horizontal line: baseline precision.

b. AUPRC on the CRISPR benchmark versus distance to TSS for methods in (**a**). Error bars: mean +/- 95% confidence intervals inferred via bootstrap (10,000 iterations).

Supplementary Notes

Supplementary Note 1. Comparisons among candidate metrics for quantifying the relationship between gene expression and enhancer accessibility

To better quantify the relationship between gene expression and enhancer accessibility, we systematically evaluated six candidate metrics in addition to the published methods (**Figure 2** and **Supplementary Table 1**). These candidate metrics include: Kendall rank correlation coefficient, Spearman's rank correlation coefficient, Pearson correlation coefficient, rank-biserial correlation, the coefficient from logistic regression, and the p-value from the Wilcoxon rank-sum test. Here, each correlation was computed across single cells within a cell type (K562). Based on CRISPR benchmarking, Kendall correlation and Spearman correlation outperformed the other four metrics, and Kendall correlation slightly outperformed Spearman correlation (**Supplementary Figure N1**).

We further assessed the effect of incorporating each metric into the scE2G model. Specifically, we treated the ABC score and the candidate metric as separate features and combined them with five additional features to retrain the model. CRISPR benchmarking demonstrated that the model incorporating Kendall correlation slightly outperformed those using the other metrics (**Supplementary Figure N1**). Additionally, Kendall correlation is computationally faster than Spearman correlation in our implementation for continuous gene expression and binarized ATAC accessibility data (Kendall: 3 hours, 1 CPU vs Spearman: 24 hours, 64 CPUs). Therefore, Kendall correlation was selected for quantifying the relationship between gene expression and enhancer accessibility.

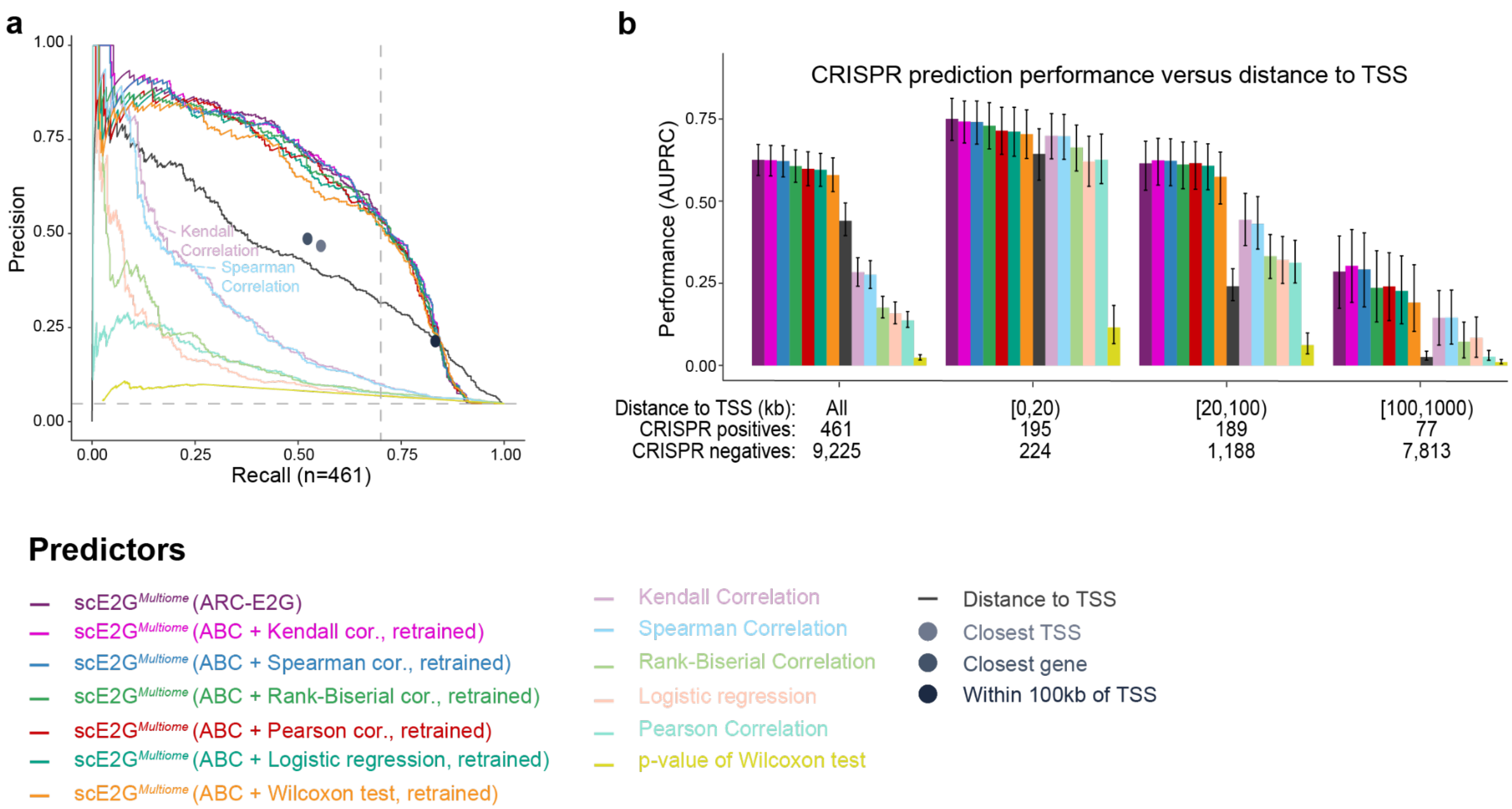

Supplementary Figure N1: CRISPR benchmarking of candidate enhancer-gene link metrics

a. Precision–recall curves displaying the performance of models on K562 CRISPR data, which were re-analyzed and assembled in Gschwind *et al.*, 2023[3] using datasets from Nasser *et al.*, 2021[4], Gasperini *et al.*, 2019[5] and Schraivogel *et al.*, 2020[6]. The benchmarking retained only element-gene (E-G) pairs with a TSS distance of less than 1Mb (9,225 tested E-G pairs, 461 positives). Each curve represents a continuous predictor. Each single dot indicates the performance of a binary predictor. Dashed vertical line marks performance at a threshold corresponding to 70% recall; dashed horizontal line indicates the proportion of CRISPR-positive E-G pairs among all CRISPR tested E-G pairs (precision value for 100% recall). scE2G^{Multiome} (ARC-E2G) refers to the integration of the Kendall score and ABC score into ARC-E2G, which was then used alongside five additional features to train

the scE2G model. scE2G^{Multiome} (ABC + feature X, retrained) indicates that the ABC score and feature X were treated as two separate features and combined with the other five features to train the scE2G model.

b. CRISPR benchmarking performance (AUPRC) of continuous predictors varies with distance to TSS. Error bars: mean +/- 95% confidence interval of AUPRC values inferred via bootstrap (10,000 iterations).

Supplementary Note 2. Summary of published predictors

1. *Signac*[7]: Signac computes the Pearson correlation between gene expression and enhancer accessibility for each enhancer-gene pair. For each enhancer, Signac selects 200 peaks with similar GC content, total accessibility level, and sequence length from different chromosomes as the background set. Then, Signac calculates the Pearson correlations between the target gene and these background peaks. A z-score is computed for each enhancer-gene pair using the formula $z = (r-\mu)/\delta$, where μ is the mean background Pearson correlation and δ is the standard deviation of the background Pearson correlations for the peak. Signac then computes a p-value for each peak using a one-sided z-test.

2. *FigR*[8]: FigR computes the Spearman correlation between gene expression and enhancer accessibility for each enhancer-gene pair. For each enhancer, FigR selects 100 peaks with similar GC content and total accessibility level as the background set. FigR then calculates the Spearman correlations between the target gene and these background peaks. A z-score is computed for each peak using the formula $z = (r-\mu)/\delta$, where μ is the mean background Spearman correlation and δ is the standard deviation of the background Spearman correlations for the peak. FigR computes a p-value for each peak using a one-sided z-test.

3. *SnapATAC*[9]: SnapATAC fits a logistic regression model for each pair of binarized enhancer accessibility and gene expression.

4. *SCENT*[10]: SCENT fits a Poisson regression between gene expression count and variables including enhancer accessibility, total UMI count, batch, and %mitochondrial UMI to identify the association between gene expression and enhancer accessibility, while controlling for interference from other factors. To estimate the error and significance, SCENT applies a bootstrapping approach to calculate the False Discovery Rate (FDR).

5. *SCENIC+*[11]: SCENIC+ uses GRNBoost2[12], which performs gradient-boosting machine regression, to estimate the importance of candidate enhancers for target gene expression. Then it categorizes the results into positive (Spearman correlation > 0.03) and negative (Spearman correlation < -0.03) interactions.

6. *Cicero*[13]: Cicero aggregates single cells into meta-cells, each composed of 50 cells similarly positioned in trajectory. It computes the correlation between promoters and enhancers across meta-cells within 500kb, and penalizes long-distance correlations using Graphical LASSO[14].

7. *DIRECT-NET*[15]: DIRECT-NET also aggregates single cells into meta-cells, then employs the XGBoost gradient boosting model to regress gene expression against all candidate enhancers across meta-cells. DIRECT-NET identifies functional enhancers based on the importance scores learned from the XGBoost model.

8. *ArchR*[16]: ArchR adopts the same meta-cell aggregation approach as Cicero, then calculates the Pearson correlation between log2-normalized gene expression and peak accessibility across meta-cells.

9. *ABC*: The ABC (activity-by-contact) model estimates the effect of a specific enhancer by multiplying the enhancer activity by the 3D contact frequency, and then dividing this product by the sum of the products of all candidate enhancer activities and their respective contact frequencies to calculate a relative contribution. In this study, we used pseudo-bulk ATAC intensity to represent enhancer activity, and inferred the 3D contact based on the power-law distribution.

10. *STARE (gABC)*[17]: STARE adopts the ABC model and extends it to the generalized ABC (gABC) model, which accounts for not only all candidate enhancers of the target gene but also all target

genes of the target enhancer. In the gABC model, enhancer activity is multiplied by the contact frequency, similar to the ABC model. However, it differs in that the result is divided by the sum of the products of enhancer activity and contact frequency for all candidate enhancers and candidate genes.

Supplementary Note 3. Benchmarks against bulk DNase-seq ENCODE-rE2G models

We compared scE2G and other ABC-based models to their counterparts based on bulk DNase-seq, including ENCODE-rE2G, which uses DNase-seq and average Hi-C, and ENCODE-rE2G^{Extended}, which takes as input DNase-seq, H3K27ac ChIP-seq, cell-type specific Hi-C, and many other epigenetic marks.

First, we compared the performance of K562 predictions on the CRISPR benchmark (**Supplementary Figure N2a,b**). Considering all tested element-gene pairs, the difference in AUPRC between scE2G^{Multiome} and ENCODE-rE2G is not significant (AUPRC = 0.626 for scE2G^{Multiome} and 0.648 for ENCODE-rE2G; $p_{bootstrap}$ for delta AUPRC = 0.139), although ENCODE-rE2G achieves higher precision at 70% recall (delta precision = 2.9%, $p_{bootstrap}$ = 0.0358). ENCODE-rE2G achieves a significantly higher AUPRC and precision than scE2G^{ATAC} (delta AUPRC = 0.059, $p_{bootstrap}$ = 0.0006; delta precision = 6.7%, $p_{bootstrap}$ = 0.0001). Suggesting that the activity assay is responsible for at least some of the discrepancy, ABC$^{A=DNase}$ achieves a significantly higher AUPRC than ABC$^{A=scATAC}$ with an even larger delta of 0.072 ($p_{bootstrap}$ = 0.0001). We see that scE2G^{Multiome}, but not scE2G^{ATAC}, has significantly higher AUPRC than ABC$^{A=DNase}$ (delta AUPRC = 0.060, $p_{bootstrap}$ = 0.0016); while both scE2G models have higher precision than ABC$^{A=DNase}$ (delta precision for scE2G^{Multiome} = 9.8%, $p_{bootstrap}$ = 0.0001; delta precision for scE2G^{ATAC} = 5.9%, $p_{bootstrap}$ = 0.0004). Considering element-gene pairs with a distance to TSS greater than 100 kb, scE2G models improve their performance relative to ENCODE-rE2G: both scE2G models achieve statistically similar AUPRC and precision to ENCODE-rE2G, and scE2G^{Multiome} achieves statistically similar AUPRC and precision to ENCODE-rE2G^{Extended} (**Supplementary Table 2**, **Supplementary Figure N2b**).

We next evaluated predictions on GM12878 cells against eQTL variants from lymphoblastoid cell lines (LCLs). When predictions are binarized at the designated thresholds, scE2G^{Multiome} has slightly lower recall than ENCODE-rE2G, but they achieve similar enrichment. scE2G^{ATAC} performs worse than ENCODE-rE2G on both metrics (**Supplementary Figure N2c**). However, evaluating enrichment of variants at a constant recall of 10% did not yield a significant difference between any of scE2G^{Multiome}, scE2G^{ATAC}, ABC$^{A=scATAC}$, ENCODE-rE2G^{Extended}, ENCODE-rE2G, or ABC$^{A=DNase}$ (**Supplementary Figure N2d**).

Finally, we evaluated model predictions from K562 cells and GM12878 lymphoblastoid cells against relevant traits (red blood cell count), mean corpuscular volume, and hemoglobin A1c for K562; lymphocyte count and combined autoimmune disorders for GM12878), which allows for a comparison with bulk models. We observe that scE2G^{Multiome} performs better than all other models, including the best-performing bulk models, at enrichment and recall of variants (**Supplementary Figure N2e**), and comparably to bulk models at linking credible sets to their causal gene (**Supplementary Figure N2f,g**).

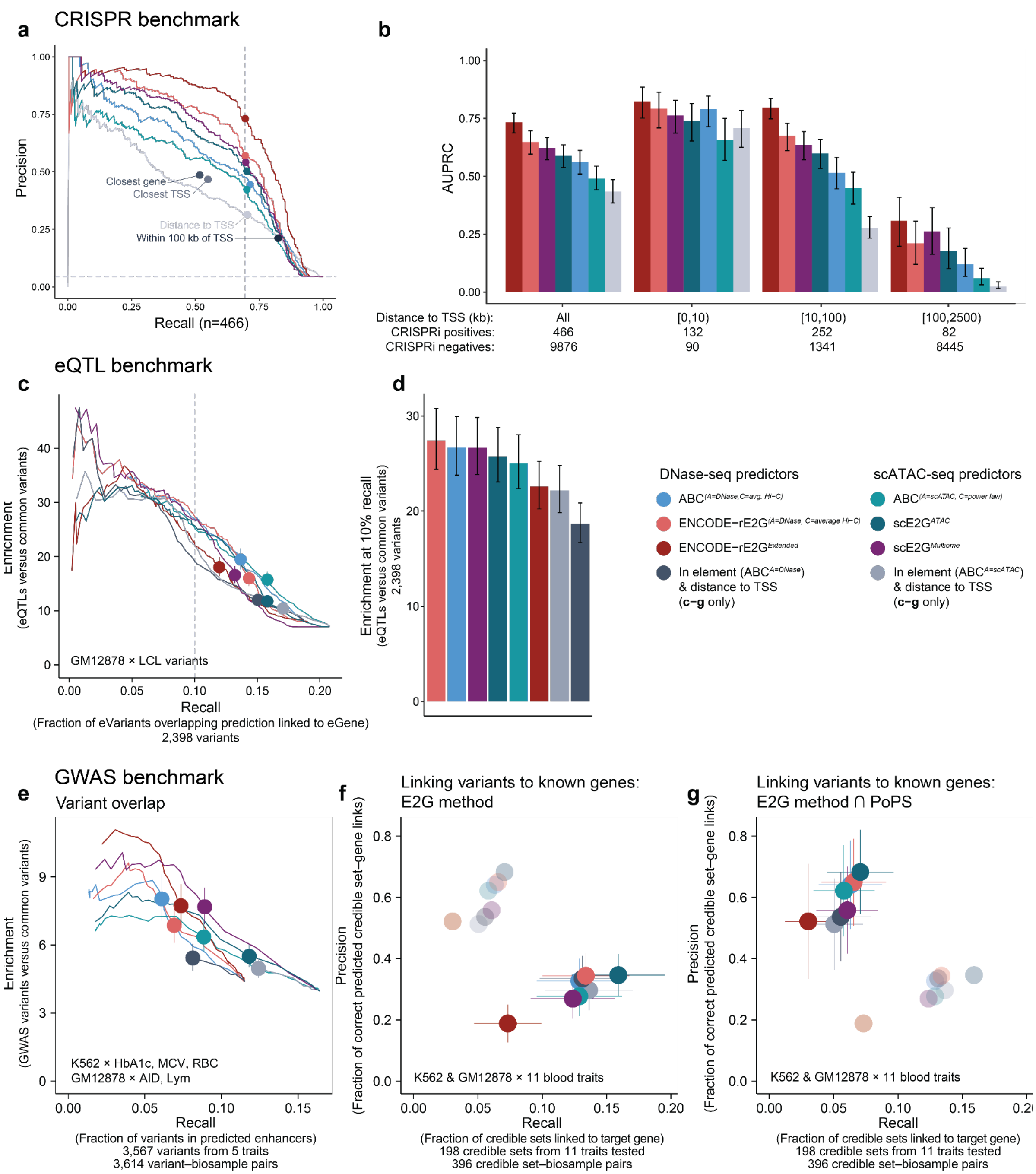

Supplementary Figure N2: Benchmarking scE2G versus bulk models

a. Precision-recall curves for K562 CRISPR data (all distance to TSS). Curves: continuous predictors; dots: performance at the designated model threshold or binary predictor. Dashed vertical line: 0% recall; dashed horizontal line: baseline precision.

b. CRISPR benchmarking performance (AUPRC) of continuous predictors for element-gene pairs stratified by distance to TSS. Error bars: mean +/- 95% confidence intervals inferred via bootstrap (10,000 iterations).

c. Enrichment-recall curves of enhancer predictions in GM12878 cells at predicting eQTLs from lymphoblastoid cell lines (LCLs). Curves: performance at ~100 thresholds; points: performance at suggested model thresholds; error bars at points: 95% confidence intervals calculated using normal approximation on log-transformed enrichment ratios.Dashed vertical line: 10% recall.

d. Enrichment of LCL eQTLs in GM12878 predictions at 10% recall. Error bars: 95% confidence intervals calculated as in (**c**).

e. Enrichment-recall curves showing overlap of fine-mapped GWAS variants for hemoglobin A1c (HbA1c), mean corpuscular volume (MCV), and red blood cell count (RBC) in K562 predictions; and variants for autoimmune disorders (AIDs) and lymphocyte count (Lym) in GM12878 predictions. Curves, points, and error bars as in (**c**).

f. Precision and recall at linking credible sets to known causal genes (inferred from independent signals with coding variants) for enhancers binarized at suggested thresholds. Predictions for K562 and GM12878 were combined and evaluated against credible sets from 11 blood-related traits (**Supplementary Table 5**). Top two genes per credible set were considered positive. Error bars: 95% confidence intervals from Wilson score interval of precision (vertical) and recall (horizontal). Faded points: performance after intersecting predictions with top two PoPS-linked genes.

g. As (**f**), but full color points indicate performance with PoPS intersection and faded points indicate performance without PoPS intersection.

Supplementary Note 4. Characteristics of CRISPR pairs affecting model performance

To better understand why scE2G performs less well on certain CRISPR pairs, we identified several contributing factors:

- **Distal versus proximal pairs**: scE2G shows reduced performance for element–gene pairs that are located more than 100 kb apart (**Figure 2c**, **Supplementary Figure N3a**). This reduction is expected. First, we estimate that ~23% of these distal pairs reflect indirect regulatory effects (where an element regulates one nearby gene A, which in turn regulates gene B) rather than direct *cis*-regulatory effects (**Methods**). As such, the "gold-standard" CRISPR data includes a mixture of true direct and indirect effects, with indirect effects being more frequent at longer distances. Second, distal pairs have a substantially lower positive rate than proximal pairs (1.2% for >100 kb versus 21.1% for <100 kb), making the prediction problem harder. These two factors—low prevalence of positives and a higher likelihood of indirect effects—together explain the reduced performance for long-distance element-gene pairs.
- **Effect size**: scE2G performs better at identifying element-gene pairs with larger effect sizes (>5%). This may be because weaker interactions are underrepresented in the training dataset (only 15% of positives, **Supplementary Figure N3b**).
- **Canonical enhancer signatures**: scE2G achieves better performance on elements with canonical enhancer signatures such as H3K27ac (**Supplementary Figure N3c**) and transcription initiation (**Supplementary Figure N3d**). For H3K27ac, this may partly reflect a selection bias in the CRISPR dataset: 83.7% of tested pairs and 98.5% of positive pairs are marked by H3K27ac. Therefore, scE2G is likely to miss enhancers lacking these signals (*e.g.*, H3K122ac-marked elements[18,19]). Furthermore, scE2G is specifically trained to predict elements that act as enhancers, and not elements that might act through other mechanisms (*e.g.,* CTCF sites that tune 3D contacts).
- **Ubiquitously-expressed genes:** scE2G underperforms for regulatory elements targeting ubiquitously expressed genes (**Supplementary Figure N3e**), which are known to be less responsive to enhancers[20–22]. Although pairs involving these genes are tested at similar rates, they show a lower positive rate (1.5% versus 7.6% for pairs with other genes in the training set), likely because most CRISPR screens are not powered to detect small effects on expression.

Across all four categories, these interactions remain difficult to predict not only for scE2G but also for baseline methods such as genomic distance (**Supplementary Figure N3**). As noted, these pairs tend to have low positive rates, are underrepresented in training data, or are difficult to detect experimentally due to limited statistical power. Together, these observations suggest that accurate prediction of such regulatory interactions remains a distinct and challenging problem which will likely require purpose-built datasets and modeling strategies.

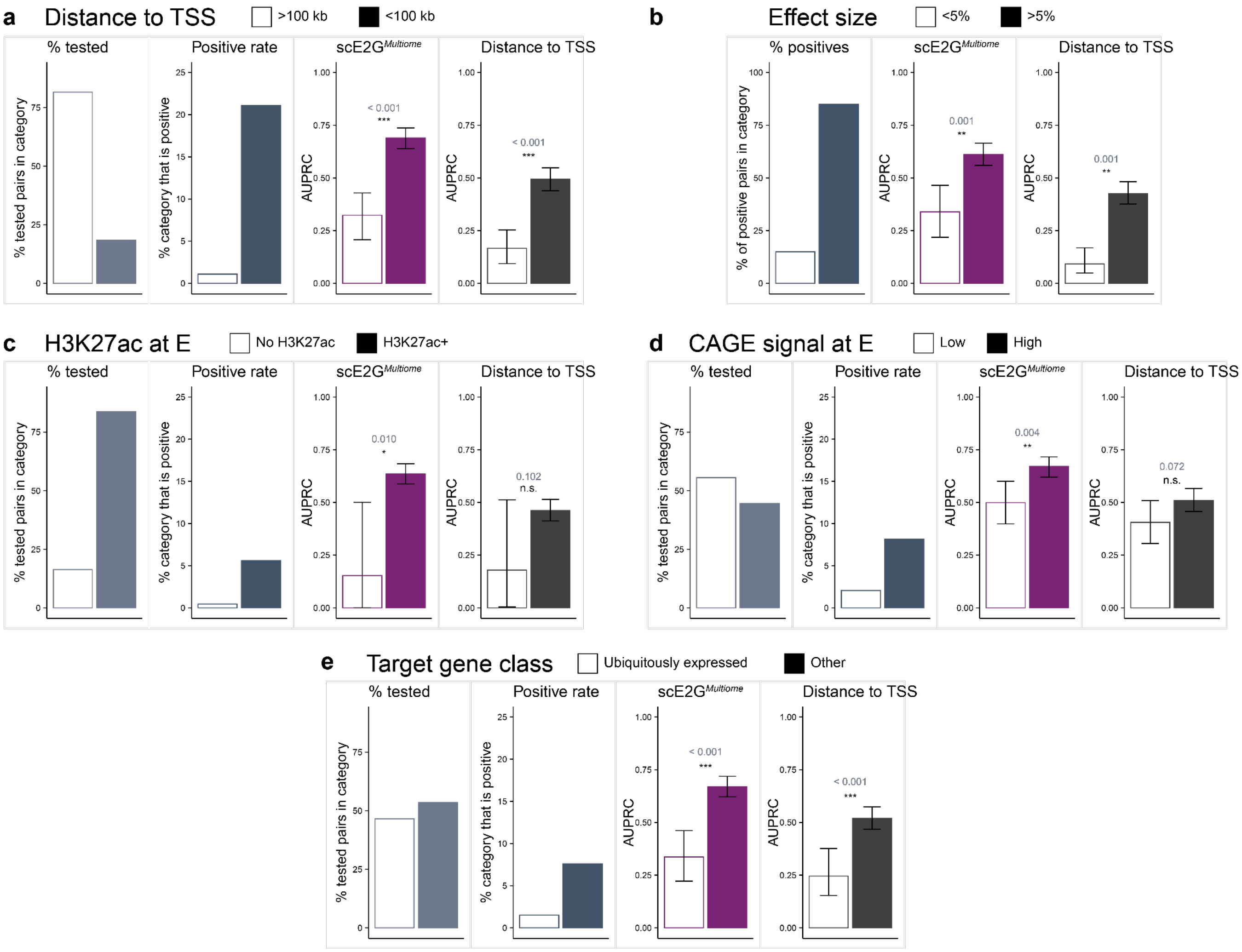

Supplementary Figure N3: scE2G performance on different types of element-gene pairs

a. Bar plots showing the percent of tested element-gene pairs in the CRISPR training dataset (all distance to TSS) with distance to TSS greater or less than 100 kb; bar plots showing the positive rate (positives / tested pairs) for distance category; AUPRC of scE2G^{Multiome} and distance to TSS on the CRISPR benchmark for pairs in each distance category. Error bars: mean +/- 95% confidence intervals inferred via bootstrap (1,000 iterations). One, two and three stars: 2-sided $p_{bootstrap}$ less than 0.05, 0.01, and 0.001, respectively, for the significance of the delta AUPRC between the categories, inferred via paired bootstrap (1,000 iterations); exact p-values in grey.

b. Bar plots showing the percent of positive element-gene pairs in the CRISPR training dataset (all distance to TSS) with effect sizes greater than or less than 5%; AUPRC of scE2G^{Multiome} and distance to TSS on the CRISPR benchmark for positives greater or less than 5%. Error bars and significance values as described in (**a**).

c. Same as (**a**), but stratifying the CRISPR dataset based on H3K27ac signal at tested element (H3K27ac+ elements overlap H3K27ac peak and/or have H3K27ac RPM in the top 50% for all non-promoter K562 elements).

d. Same as (**a**), but stratifying the CRISPR data based on nascent transcription observed at the tested element (highly transcribed "high CAGE" elements defined based on overlap with regulatory elements predicted from K562 nuclei SLIC-CAGE data (Einarsson and Navamajiti *et al.*, in preparation, https://github.com/anderssonlab/PRIME).

e. Same as (**a**), but stratifying the CRISPR data based on target gene class.

Supplementary Note 5. Enrichment of heritability of GWAS traits in scE2G enhancers by S-LDSC

We used stratified linkage disequilibrium score regression (S-LDSC) to identify cell types that had predicted enhancers enriched for genome-wide heritability of GWAS traits[23,24]. We computed enrichment of heritability for 104 traits[25] in scE2G^{Multiome} enhancers (excluding promoters) for 46 cell types and identified 339 significant trait-cell type pairs (**Supplementary Table 8**, **Supplementary Figure N4**, $q < 0.05$ after multiple hypothesis correction for each trait adjusting for the number of cell types compared) (7% of pairs). Of the 104 traits, 42 had significant heritability enrichment in at least one of the cell types. Subsetting to 25 traits relevant to the cell types analyzed (combined autoimmune disorders, basophil count, body fat percentage, body mass index, body weight, eosinophil count, random glucose, hemoglobin, hemoglobin A1c, hematocrit, hypothyroidism, inflammatory bowel disease, IGF1, lymphocyte count, mean corpuscular hemoglobin, mean corpuscular hemoglobin concentration, mean corpuscular volume, monocyte count, neutrophil count, platelet count, red blood cell count, type 2 diabetes, type 2 diabetes (BMI-adjusted), white blood cell count, waist-height-ratio-adjusted body mass index), 214 trait-cell type pairs had significant enrichment (18.6% of pairs).

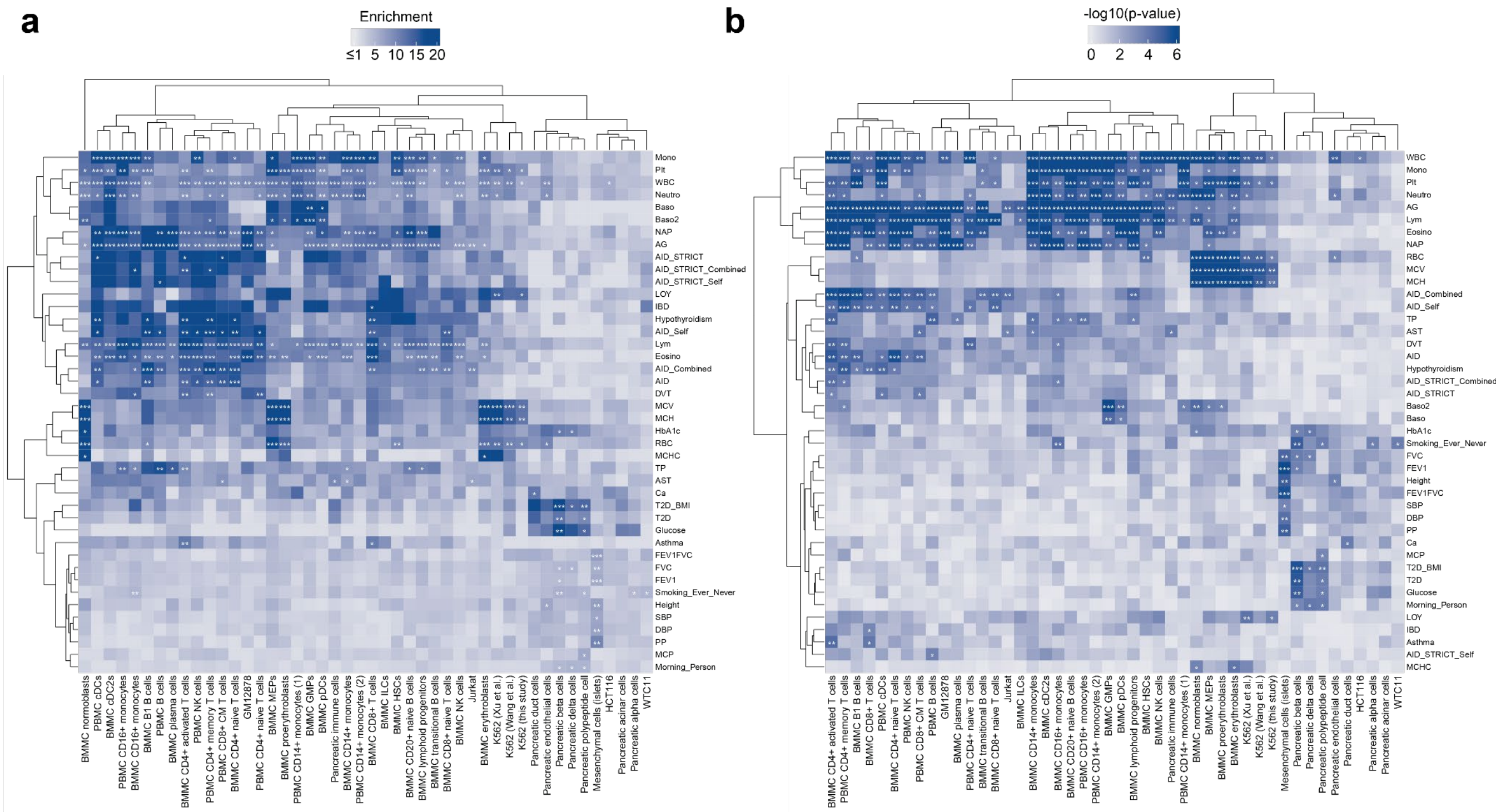

Supplementary Figure N4: S-LDSC heritability enrichment in scE2G enhancers

a. Heatmap showing genome-wide enrichment computed with S-LDSC for 42 GWAS traits in scE2G^{Multiome} predictions for 46 cell types. Only traits with significant enrichment (q < 0.05) in at least one cell type are shown. Stars indicate significance based on enrichment q-values (FDR-adjusted p-values): three stars indicate q < 0.001, two stars indicate q < 0.01, and one star indicates q < 0.05. The color scale is limited at the low end to an enrichment of 1. Rows and columns are hierarchically clustered with the "complete" method based on Euclidean distance.

b. Heatmap showing -log10(p-value) for genome-wide enrichment computed with S-LDSC for 42 GWAS traits in scE2G^{Multiome} predictions for 46 cell types. As in **a**, only traits with significant enrichment (q < 0.05) in at least one cell type are shown. Stars indicate significance based on enrichment q-values as in **a**. Rows and columns are hierarchically clustered with the "complete" method based on Euclidean distance.

Supplementary Table Descriptions

Supplementary Table 1. Published models
Summary of previously published models benchmarked here, including the score metrics and thresholding criteria used for each analysis.

Supplementary Table 2. CRISPR benchmark pairwise comparisons
Results from bootstrapping (1,000 iterations) of difference in performance between each pair of models on the CRISPR benchmark.

Supplementary Table 3. CRISPR benchmark performance intervals
Table with results from bootstrapping (10,000 iterations) of confidence intervals for performance metrics for each model on the CRISPR benchmark.

Supplementary Table 4. eQTL benchmarking cell type pairings
Description of prediction and eQTL cell type pairings used for eQTL benchmarking.

Supplementary Table 5. GWAS benchmarking trait–cell type pairings
Description of traits and prediction cell type pairings used for GWAS benchmarking.

Supplementary Table 6. scE2G predictions
Descriptions of each cell type to which scE2G was applied and links to scE2G^{Multiome} and scE2G^{ATAC} predictions for each cell type. See https://github.com/anderssonlab/scE2G_analysis/blob/v1.1/5.Prediction_Properties/scE2G_prediction_columns.tsv for descriptions of each column in the scE2G prediction files.

Supplementary Table 7. scE2G^{Multiome} and scE2G^{ATAC} prediction properties
Table describing genome-wide properties of scE2G^{Multiome} and scE2G^{ATAC} predictions in each cell type. See https://github.com/anderssonlab/scE2G_analysis/blob/v1.1/5.Prediction_Properties/prediction_properties.tsv for definitions of each metric.

Supplementary Table 8. GWAS trait heritability enrichment in scE2G^{Multiome} enhancers
Results from stratified linkage-disequilibrium score regression (S-LDSC) analysis of heritability enrichment of GWAS traits in scE2G^{Multiome} enhancers. Results are sorted by increasing enrichment p-value.

Supplementary Table 9. Gene prioritization for noncoding GWAS variants
Gene prioritization tables for BMMCs, PBMCs, and pancreatic islets detailing each credible set, causal gene, and cell types are predicted by scE2G^{Multiome}. For BMMC and PBMC cell types, the following traits were considered: RBC, WBC, Mono, Lym, Eosino, Baso, Neutro, Plt, MCH, MCHC, MCV, Hb, HbA1c, Ht, Glucose. For pancreatic islet cell types, the following traits were considered: Glucose, HbA1c, WHRadjBMI, Ht, T2D_BMI. Only trait-cell type pairs in which variants for the trait were significantly enriched in enhancers by at least 5-fold were prioritized.

Supplementary Table 10. 10x Multiome data sources
Sources for 10x Multiome datasets used in this study.

Supplementary Table 11. 10x Multiome data quality control thresholds
Thresholds used to select high-quality cells from each 10x Multiome dataset.

Supplementary Methods

1. Genome build

All coordinates in the human genome are reported using build GRCh38 unless otherwise specified.

2. Gene reference files for scE2G and benchmarking analyses

We used a consistent set of gene and promoter coordinates when building scE2G and conducting all benchmarking analyses. These files (available at https://github.com/EngreitzLab/scE2G/tree/v1.3/resources/genome_annotations) contain one promoter per gene symbol, which we defined as a 500 base pair region centered around the RefSeq[26] TSS with the largest number of coding isoforms. To curate the set of genes, we matched the gene symbols with Ensembl IDs using the HUGO database[27] and manual annotation, then used the GENCODE v29 database[28] to annotate each gene with its "gene type". We retained genes that 1) were included in the GENCODE v29 database and 2) had a gene type of "protein_coding," "processed_transcript," or "lincRNA" for a total of 20,666 genes. To facilitate accurate mapping of read counts from scRNA-seq count matrices processed with GENCODE v43[29], we then filtered this list to the 20,531 genes with exactly one corresponding gene entry from GENCODE v43.

3. Collecting and preprocessing of 10x Multiome data

3.1. *Build reference genome:* The GRCh38 genome FASTA file was downloaded from the ENCODE Project (https://www.encodeproject.org/files/GRCh38_no_alt_analysis_set_GCA_000001405.15/@@download/GRCh38_no_alt_analysis_set_GCA_000001405.15.fasta.gz), and the gene annotation GTF file was obtained from GENCODE (https://ftp.ebi.ac.uk/pub/databases/gencode/Gencode_human/release_43/gencode.v43.chr_patch_hapl_scaff.annotation.gtf.gz). We created a reference using the `cellranger-arc` (v2.0.2) "`mkref`" tool, following the guidelines provided by 10x Genomics (https://www.10xgenomics.com/support/software/cell-ranger-arc/latest/analysis/inputs/mkref). The Wang *et al.* K562 dataset mixes human K562 and mouse A20 cells. To distinguish human K562 cells from mouse A20 cells, we downloaded the mouse reference (mm10) from https://cf.10xgenomics.com/supp/cell-arc/refdata-cellranger-arc-mm10-2020-A-2.0.0.tar.gz

3.2. *Prepare raw FASTQ files:* For the Xu *et al.* K562[1] and PBMC datasets, we directly downloaded the raw FASTQ files (**Supplementary Table 10**). For the GM12878[30], BMMC[31] datasets, we downloaded BAM format files (**Supplementary Table 10**), and converted the BAM files to FASTQ files using the cellranger-arc (v2.0.2) "bamtofastq" tool (https://www.10xgenomics.com/support/software/cell-ranger/latest/miscellaneous/cr-bamtofastq). For the Wang *et al.* K562[2] and pancreatic islets[32] datasets, we downloaded SRA format files using the sratoolkit (v3.0.0) with the commands "`prefetch -X 100G -pv -O sra SRR_accession_number`", and converted SRA files to FASTQ files using "`fastq-dump --split-files --gzip --outdir output_directory_path sra_file_path`". We renamed the FASTQ files in accordance with the 10x Genomics guidelines (https://www.10xgenomics.com/support/software/cell-ranger-

arc/latest/analysis/inputs/specifying-input-fastq-count) if the file names did not meet the requirements of cellranger-arc count.

3.3. *Run cellranger-arc pipeline:* We created a libraries CSV file for each sample to delineate the input file paths, and ran the `cellranger-arc count` tool following the 10x Genomics guidelines (https://www.10xgenomics.com/support/software/cell-ranger-arc/latest/analysis/running-pipelines/single-library-analysis).

3.4. *Obtaining K562 (this study), HCT116, and Jurkat datasets:* K562 cells were obtained from the authors of a previous study[42]; HCT116 (accession CCL-247) and Jurkat (accession TIB-152) cells were obtained from ATCC. We downloaded Cell Ranger outputs from the IGVF Data Portal (**Supplementary Table 10**). The count matrices were generated using `cellranger count` (v6.0.0) with the GRCh38 reference transcriptome. Fragment files were generated using `cellranger-atac count` (v2.0.0) with the GRCh38 reference genome.

3.5. *Obtaining WTC11 dataset:* We collected a 10x Multiome dataset in WTC11 cells, mixed with other cells differentiated toward a hepatic progenitor lineage. To do so, we seeded human inducible pluripotent stem cells (WTC11) harboring doxycycline-inducible KRAB-dCas9-P2A-mCherry[33] (gift from Ioannis Karakikes, Stanford University) in 6-well plates coated with 1:100 LDEV-Free Reduced Growth Factor Basement Membrane Matrix (Thermo Fisher Scientific). The culture medium used was StemFlex Medium supplemented with 10% StemFlex supplement (SF complete media, Thermo Fisher Scientific), 2 µl of Normocin per ml of media (1:500 final, Invivogen), and 0.4 µl of 10 mM Rock Inhibitor (Y-27632 Dihydrochloride, STEMCELL Technologies). After 24 hours of seeding, we continued to maintain the cells with SF complete media without Rock Inhibitor and passaged them with 1 ml of accutase (STEMCELL Technologies) as necessary, aiming to maintain an 80-90% confluence to prevent stacked cell growth. We differentiated cells into hepatocyte progenitors following a published protocol[34] (detailed in the GEO submission methods) and collected samples on days 0, 2, 4, and 7 of differentiation. We prepared single-cell multiomic libraries using the 10x Genomics Chromium Single Cell Multiome ATAC + Gene Expression kit (PN-1000284) combining nuclei from each timepoint at a 1:1:2:4 ratio. We sequenced ATAC libraries on an Illumina NovaSeq SP and gene expression (GEX) libraries on an Illumina NextSeq 2000 with a P2 flow cell.

We generated count matrices and fragment files using `cellranger-arc count` (v2.0.2) with the GRCh38 reference. The Cell Ranger outputs are deposited on GEO (GSE302246).

4. Quality control for 10x Multiome data

4.1. *Quality control metrics:* We implemented following metrics to select of high-quality cells from the 10x Multiome data:

- `gex_umis_count`: UMI count in scRNA-seq
- `atac_fragments`: fragment count in scATAC-seq

- `nucleosome_signal`: ratio of mononucleosome fragments to nucleosome-free fragments
- `TSS.enrichment`: scATAC-seq TSS enrichment score defined by ENCODE (https://www.encodeproject.org/data-standards/terms/)
- `percent.mt`: percentage of mitochondrial transcripts

The `gex_umis_count` and `atac_fragments` metrics were extracted from the "per_barcode_metrics.csv" file, which is generated by the cellranger-arc count pipeline. The `atac_fragments`, `TSS.enrichment`, and `percent.mt` metrics were calculated using the Signac[7] (v1.13.0) and Seurat[35,36] (v5.0.3) R functions `Signac::NucleosomeSignal`, `Signac::TSSEnrichment`, and `Seurat::PercentageFeatureSet(pattern = "^MT-")`, respectively. We defined thresholds for these metrics based on their distributions within each dataset. These thresholds are detailed in **Supplementary Table 11**.

4.2. *Identifying human K562 cells in Wang et al.*[2] *dataset:* In the Wang *et al.*[2] dataset, human K562 cells were mixed with mouse A20 cells. We calculated the percentage of mapped reads (% mapped = # mapped_reads / # raw_reads) to the human genome, and selected cells with % mapped > 95% in the human genome for both scRNA-seq and scATAC-seq as human K562 cells.

4.3. *K562 (this study), HCT116, and Jurkat quality control*: Cells were thresholded to exclude cells with fewer than 10^3 total fragments before a joint peak matrix was computed across 10x Genomics microfluidic lanes. Peak matrices were then thresholded to exclude cells with fewer than 100 RNA UMIs, merged, and pre-processed using standard Seurat and Signac workflows. Sample of origin demultiplexing was then performed using the deMULTIplex2[37] R package (v1.0.1) and doublet-enriched clusters and unclassified cells were removed. Cleaned data was then re-processed using Seurat and Signac, and the relevant cell types were subsetted for downstream analyses.

5. Cell-type annotation

5.1. *BMMC dataset:* We downloaded the processed h5ad file from Luecken *et al.*[31] (available at NCBI GEO https://www.ncbi.nlm.nih.gov/geo/query/acc.cgi?acc=GSE194122) and extracted the cell type information from it.

5.2. *Pancreatic islet dataset:* We conducted scRNA-seq analysis using Seurat (v5.0.3)[35] to perform cell clustering:

(i) Normalized the RNA UMI count matrix using `Seurat::NormalizeData`.
(ii) Identified variable genes with `Seurat::FindVariableFeatures`.
(iii) Scaled the normalized RNA matrix for variable genes using `Seurat::ScaleData`.
(iv) Performed PCA using `Seurat::RunPCA`.
(v) Defined neighboring cells using `Seurat::FindNeighbors`.
(vi) Identify cell clusters using `Seurat::FindClusters`.

Subsequently, we defined the corresponding cell types based on marker gene expression: beta cell (*INS*+), alpha cell (*GCG*+), delta cell (*SST*+), PP cell (*PPY*+), ductal cell (*CFTR*+),

acinar cell (*REG1A+*), endothelial cell (*PECAM1+*), mesenchymal cell (*COL3A1+*) and immune cell (*PTPRC+*). Cell clusters expressing multiple markers were considered as doublets. Additionally, beta cells from one donor (A0027) exhibited notable batch effects. Both doublets and beta cell clusters with batch effects were excluded from downstream analyses.

5.3. *PBMC dataset*: To facilitate the annotation of cell types in PBMC 10x Multiome data, we downloaded the Healthy Hematopoiesis scRNA-seq dataset from https://github.com/GreenleafLab/MPAL-Single-Cell-2019. We then employed Harmony[38] (v1.2.0) to integrate the PBMC and Healthy Hematopoiesis scRNA-seq data. Based on the results from Harmony, we performed cell clustering using Seurat. Utilizing the cell type annotation information from the Healthy Hematopoiesis scRNA-seq dataset, we identified the most prevalent cell type in our new clustering results as the designated cell type.

5.4. *WTC11 dataset:* The iPSC cell state was identified based on expression of the iPSC marker genes *POU5F1* and *NANOG*.

6. Preparing input files for scE2G

The scE2G pipeline requires input data including RNA count matrices, ATAC fragment files, and their corresponding *.tbi index files for each cell cluster. Example input data files are available here:
https://github.com/EngreitzLab/scE2G/tree/v1.2/resources/example_chr22_multiome_cluster

6.1. *RNA count matrix:* We exported the RNA count matrix from the Seurat object in CSV format. Rows of the matrix correspond to genes, while columns correspond to cells. The first row contains the names of the cells, and the first column contains the names of the genes.

6.2. *ATAC fragment file:* We utilized the R function `Signac::SplitFragments` to split raw fragment files into separate fragment files for each sample and each cell type. We then merged fragment files of the same cell type into a single fragment file. If the cell names in the fourth column of the fragment file do not match the cell names in the RNA count matrix (*e.g.*, cell names in the RNA count matrix include a sample name prefix), it is necessary to modify the cell names in the fragment file to match those in the RNA count matrix. Subsequently, we sorted each cell type's fragment file using `sortBed` from bedtools (v2.31.0), and then compressed and indexed each fragment file using bgzip and tabix from htslib[39] (v1.18), respectively.

7. Kendall correlation

We applied a non-parametric measurement, the Kendall correlation (also known as Kendall rank correlation coefficient and Kendall's τ coefficient)[40], to quantify the correlation between element accessibility (binarized values) and gene expression (continuous values). We extracted MACS2 (v.2.2.9)[41] peaks that overlap with candidate elements defined in the ABC pipeline (**Supplementary Methods 9.1.1**) and generated the ATAC count matrix using the `Signac::FeatureMatrix` function. We binarized the ATAC fragment count matrix using R `Signac::BinarizeCounts` function, and normalized the RNA UMI count matrix using R

`Seurat::NormalizeData` function. We then computed the Kendall correlation τ_b between binarized element accessibility and normalized gene expression for each candidate element-gene pairs.

7.1. Definition of Kendall correlation: The Kendall correlation is based on the concordance and discordance between pairs of observations. A pair is considered concordant if the ranks for both elements agree (i.e., if the element accessibility in one pair is higher than in another, the same relationship holds for the gene expression). Conversely, a pair is discordant if the ranks disagree. When all values for both variables are unique, τ is calculated as $\tau = \frac{n_c - n_d}{n_0}$, where n_c is the number of concordant pairs, n_d is the number of discordant pairs, and n_0 is the total number of pairs. Ranking of element accessibility (binary) causes many ties. To account for these, we used the τ_b formula: $\tau_b = \frac{n_c - n_d}{\sqrt{(n_0 - n_1) \cdot (n_0 - n_2)}}$, where $n_0 = \frac{n(n-1)}{2}$, $n_1 = \sum_i \frac{t_i(t_i - 1)}{2}$, $n_2 = \sum_j \frac{u_j(u_j - 1)}{2}$; n is the number of cells, t_i and u_j represent the number of tied values in the ith and j^{th} group of ties for the gene expression and element accessibility, respectively. During Kendall correlation calculation, we keep all cells without any specific adjustments to remove or impute gene expression dropouts.

7.2. Fast algorithm to compute the Kendall correlation: To accelerate the calculation of Kendall correlation, we developed a fast algorithm specifically for binarized element accessibility and continuous gene expression (written in R and Rcpp). The algorithm calculates the Kendall correlation for one gene and multiple elements at a time. First, we sort the cells based on gene expression in descending order. Using this sorted order, we then reorder the corresponding element accessibility values. This sorted matrix is then processed using an Rcpp function, `count_diff`. A cumulative sum is computed over the reordered element accessibility: (1) If a cell has element accessibility = 0, it forms concordant pairs with all previously ranked cells where element accessibility = 1 (the cumulative sum up to that cell). This is because those previous cells have higher gene expression (due to the sorting) and higher accessibility (1 > 0), satisfying the concordance condition. (2) If a cell has element accessibility = 1, it forms discordant pairs with all previously ranked cells where element accessibility = 0. The number of such discordant pairs is calculated as (rank - cumulative sum) at that point. Again, because the previous cells have higher gene expression but lower accessibility (0 < 1), this indicates discordance. The `count_diff` function avoids explicit pairwise comparisons by leveraging the sorted order and cumulative sum, making the algorithm more efficient to compute the difference between concordant and discordant pairs for each element (**Extended Data Figure 1**). Below is pseudocode for the `count_diff` function:

Function count_diff(element_accessibility_matrix_sorted)
Input: element_accessibility_matrix_sorted, a logical matrix with rows sorted by gene expression

Initialize n as number of rows in element_accessibility_matrix_sorted
Initialize m as number of columns in element_accessibility_matrix_sorted
Initialize result as a numeric vector of length m

```
    For j from 0 to m-1
        Initialize concordant to 0
        Initialize disconcordant to 0
        Initialize cumsum to 0

        For i from 0 to n-1
            Set isAccessible to the value at element_accessibility_matrix_sorted[i, j] (true for accessible, false for inaccessible)
            Add isAccessible to cumsum

            If isAccessible is true
                Increment disconcordant by (i + 1 - cumsum)
            Else
                Increment concordant by cumsum

        Set result[j] to concordant - disconcordant

    Return result
End Function
```

Subsequently, our R function adjusts for ties in gene expression values to ensure accurate correlation calculations (we have already accounted for ties in binarized ATAC data in `count_diff` function). Finally, we calculate Kendall correlation τ_b using these adjusted values. We retain the Kendall correlation for all candidate element-gene pairs regardless of the statistical significance.
See https://github.com/EngreitzLab/scE2G/blob/v1.3/workflow/scripts/feature_computation/compute_kendall.R for the full implementation.

8. ARC-E2G score

According to our CRISPR benchmarking results, the ABC model and the Kendall correlation each performed best as single features dependent and independent of 3D contact or distance, respectively (**Figure 2b,c**). However, the performance of the Kendall correlation, and therefore its weight in the scE2G logistic regression model, was dependent on sequencing and cell depth. Accordingly, to improve robustness across sequencing depths, we integrated the ABC model and the Kendall correlation into a single feature called Activity, Responsiveness, and Contact (ARC)-E2G score.

8.1. *Motivation for the ARC-E2G score:* We observed that both the log-transformed ABC score (ln(ABC score)) and the Kendall correlation approximately follow a normal distribution (**Extended Data Figure 2a,b**). To ensure comparability in magnitude, we standardized both features by subtracting the mean (μ) and dividing by the standard deviation (σ). We found that the standardized Kendall correlation and scaled ln(ABC score) are linearly positively correlated, and that CRISPR-positive element-gene pairs are preferentially enriched in the area where both values are high compared to CRISPR-negative pairs

(**Extended Data Figure 2c,d**). Based on these observations, we designed the ARC-E2G score to capture this joint enrichment signal and to ensure that the ordering of the ARC-E2G score is equivalent to the ordering of the CRISPR-positive enrichment component.

8.2. *Computing the ARC-E2G score:* To quantify the relationship between ABC score and Kendall correlation, we fitted a linear regression: $ln(ABC\ score)_{scaled} = \beta * \tau_{scaled} + \alpha$, where $ln(ABC\ score)_{scaled}$ is scaled $ln(ABC\ score)$, τ_{scaled} is scaled Kendall correlation, β and α are the slope and intercept in the linear regression, respectively.

We considered that points on the orthogonal line to this regression line, described by $ln(ABC\ score)_{scaled} = -1 / \beta * \tau_{scaled} + \alpha'$, should be independent to CRISPR[+] enrichment and have the same ARC-E2G score, where α' is the intercept of the orthogonal line on the $ln(ABC\ score)_{scaled}$ axis. Higher values of α' indicate a stronger likelihood that the line corresponds to CRISPR-positive pairs. The intercept α' is calculated as follows:

$$\alpha' = ln(ABC\ score)_{scaled} + (1 / \beta) * \tau_{scaled}$$
$$= (ln(ABC\ score) - \mu_{ln(ABC\ score)}) / \sigma_{ln(ABC\ score)} + (1 / \beta) * (\tau - \mu_\tau) / \sigma_\tau$$

We define the ARC-E2G score as follows to ensure it aligns with the magnitude of the ABC score and increases monotonically with the intercept α' of the orthogonal line:

$$\alpha' = (ln(ARC\text{-}E2G\ score) - \mu_{ln(ABC\ score)}) / \sigma_{ln(ABC\ score)}$$

Thus,

$$(ln(ARC\text{-}E2G\ score) - \mu_{ln(ABC\ score)}) / \sigma_{ln(ABC\ score)}$$
$$= (ln(ABC\ score) - \mu_{ln(ABC\ score)}) / \sigma_{ln(ABC\ score)} + (1 / \beta) * (\tau - \mu_\tau) / \sigma_\tau$$

So

$$ln(ARC\text{-}E2G\ score) = ln(ABC\ score) + (1 / \beta) * (\sigma_{ln(ABC\ score)} / \sigma_\tau) * \tau - (1 / \beta) * (\sigma_{ln(ABC\ score)} / \sigma_\tau) * \mu_\tau$$

where $\mu_{ln(ABC\ score)}$ and μ_τ are means of $ln(ABC\ score)$ and τ; and $\sigma_{ln(ABC\ score)}$ and σ_τ are standard deviations of $ln(ABC\ score)$ and τ, respectively. In practice, since $(1 / \beta) * (\sigma_{ln(ABC\ score)} / \sigma_\tau) * \mu_\tau$ is a constant close to 0 (as μ_τ is close to 0), we ignore this term in computation, so

$$ln(ARC\text{-}E2G\ score) = ln(ABC\ score) + (1 / \beta) * (\sigma_{ln(ABC\ score)} / \sigma_\tau) * \tau$$

Interestingly, we observed that the ranking of ARC-E2G score corresponds well with the density of CRISPR-positive relative to CRISPR-negative pairs (**Extended Data Figure 2d,e**).

See https://github.com/EngreitzLab/scE2G/blob/v1.3/workflow/scripts/feature_computation/compute_arc_e2g.R for the full implementation.

9. Training and applying scE2G

We implemented a training and application pipeline for scE2G that applies the Activity-by-Contact (ABC)[42] codebase to define all candidate element-gene pairs, quantify activity at each element, and compute the ABC score for each element-gene pair, using a pseudobulk scATAC-seq fragment file corresponding to a given cell type. The pipeline then uses the ENCODE-rE2G[3] codebase to annotate element-gene pairs with additional activity-based features. In the scE2G pipeline, we compute the Kendall correlation between element accessibility and gene expression, calculate the ARC-E2G score that integrates the Kendall correlation and ABC score (**Methods 7, 8**), and implement additional score normalization and filtering to account for sequencing depth variability and gene expression data. The scE2G pipeline, including ENCODE-rE2G submodule and ABC sub-submodule, is implemented here: https://github.com/EngreitzLab/scE2G/tree/v1.3

9.1. Processing pseudobulk ATAC-seq data (implemented in ABC pipeline)

9.1.1. *Define candidate element-gene pairs:* We call peaks on a tagAlign file generated from the pseudobulk ATAC fragment file for each cell cluster using MACS2 with parameters from the ENCODE ATAC-seq Processing Pipeline[43]. We count reads in each peak with P<0.1 and identify the 150,000 peaks with the highest read counts. We take 500 base-pair elements centered on the summits of these peaks, merge overlapping elements, remove elements overlapping ENCODE blacklisted regions (regions of the genome that have been observed to accumulate anomalous number of reads in epigenetic sequencing experiments)[44], add 500 base-pair promoter elements centered on the transcription start site of each gene (from **Methods 2**), and again merge overlapping regions. We define the resulting list of regions as 'candidate elements,' and annotate each as a promoter (elements overlapping regions from the promoter reference file), genic (elements within an annotated gene body), or intergenic (all other elements). We define all genic and intergenic elements as 'distal' elements. We generate the list of candidate element-gene pairs by computing all combinations of candidate elements with any promoter within 5 Mb.

9.1.2. *Annotating candidate elements with normalized ATAC signal*: We count reads in each candidate element from the tagAlign file, and double the count for elements on the sex chromosomes to simulate two copies. We then rank-normalize the ATAC reads per million for distal elements and promoters to K562 reference values (derived from the original ABC paper)[42] for each rank in their respective category. Briefly, we rank candidate elements from each category by ATAC reads per million, and replace the read count with the interpolated read count from the reference list. This normalization is necessary for comparison of values across biosamples and minimizes technical variability from the experimental set up. The ATAC read count after normalization is used in all downstream calculations.

9.1.3. *Determining active genes*: We remove element-gene pairs from consideration for any gene that has an 'inactive promoter' based on the promoter ATAC signal. Here, we exclude genes with a promoter activity quantile less than 0.3.

9.1.4. *Estimated 3D contact between each element-gene pair*: Because it is rare to have cell-type specific Hi-C data corresponding to cell clusters from single-cell experiments, we estimate 3D contact using a power law relationship with genomic distance. We have previously shown that a relationship in the form of contact = exp(scale + -1 * gamma * ln(distance)) fits Hi-C contact frequencies well[42]. The *gamma* and *scale* parameters were previously computed by fitting a linear regression between log-transformed distance and K562 Hi-C contact, binned at 5-kb resolution[3]. For genomic distances below 5 kb, we set estimated contact as the power law contact at a distance of 5 kb.

9.1.5. *Calculate ABC score for each element-gene pair*: We calculate the ABC score between each element-gene pair on a per-gene basis. The numerator of the ABC score for a given element-gene pair is the normalized ATAC signal at the element multiplied by the power law contact between the element and gene. The denominator of the ABC score for a given gene is equal to the sum across all candidate element-gene pairs of the normalized ATAC signal at the element multiplied by the power law contact between the element and gene. Finally, the ABC scores for 'self-promoter' element-gene pairs (promoters linked to their target gene) are set to 1.

9.2. Annotating genome-wide element-gene pairs with additional features: In addition to the ABC score, the Kendall correlation, and ARC-E2G score (**Methods 7, 8**), we annotated each element-gene pair with nine other values related to chromatin accessibility, genomic distance, and gene density to use as input features for scE2G. We included the following four features that were output directly from the ABC pipeline: distance to transcription start site, ATAC signal at the candidate element, ATAC signal at the promoter, and power law contact. We further computed the number of TSSs and number of other elements between the element and promoter, as well as the number and sum of activity of peaks within 5 kb of the element (implemented in ENCODE-rE2G pipeline). Finally, each promoter was classified based on 'ubiquitous expression.'

9.2.1. *Classifying promoters as ubiquitously-expressed:* We annotate promoters as ubiquitously-expressed using a classification obtained from previous work[20]. Briefly, CAGE data from the FANTOM5 consortium across 1,829 biosamples (downloaded from https://fantom.gsc.riken.jp/5/datafiles/latest/extra/gene_level_expression/hg19.gene_phase1and2combined_tpm.osc.txt.gz on 19 February 2022) was analyzed to obtain expression (in TPM) for each gene. Ubiquitously-expressed genes were defined as those expressed at ≧1 TPM in ≧95% of biosamples.

9.3. Training scE2G on CRISPR perturbation dataset (implemented in ENCODE-rE2G pipeline): We trained logistic regression classifiers on the K562 CRISPR perturbation dataset, where positive element-gene pairs were defined as cases where perturbing the element had a significant and negative effect size on expression of the target gene. Using this framework, we selected final sets of features for two models: scE2G^{ATAC} which uses just scATAC-seq data and scE2G^{Multiome} which uses multiomic data.

9.3.1. *Training logistic regression classifiers*: We annotated the element-gene pairs in the CRISPR dataset by overlapping them with the genome-wide element-gene pairs (at least one base pair overlap) computed on a deeply-sequenced K562 10x Multiome dataset[1]. We applied a variance-stabilizing transformation $log(|x| + \epsilon)$, with $\epsilon = 0.01$, to all the features before feeding them into a logistic regression model implemented with sklearn's `LogisticRegression` function. To maintain calibrated probability scores as an output of the logistic regression, we did not use any penalty. To evaluate the performance of the logistic regression models on the CRISPR dataset, we used chromosome-wise cross validation by holding out one chromosome, training a model on the rest, and using this model to score each element-gene pair on the held-out chromosome. All performance measures for scE2G on the CRISPR training dataset report this cross-validation result (*e.g.*, **Figure 2–4**, **Supplementary Figs. 1**, **3–6**, **Extended Data Figs. 4, 7, 8**). For scE2G^{Multiome}, we then implement a gene expression filter (specified by a minimum TPM) by setting the score of all element-gene pairs where the gene is expressed below the TPM threshold to 0. We also train a model on all chromosomes, which is used to apply scE2G to new cell types.

9.3.2. *Selecting features for final models*: To select the final set of features for each scE2G model, we used several approaches. We applied a best-subsets approach to calculate the area under the precision-recall curve (AUPRC) on the CRISPR benchmark for models comprising every possible subset of the input features, and selected the set of features for each model that minimized number of features while maintaining near-optimal performance. We performed additional feature analyses including forward sequential feature selection (iteratively choosing the feature that contributes to the largest increase in AUPRC) and ablation of single features or categories of features to validate the importance of our chosen features in the final models. When choosing features for scE2G^{Multiome}, we used initial feature sets with ABC score and the Kendall correlation as separate features to determine the contribution of each feature independently, as well as with the ARC-E2G score in place of the two features. We saw similar results with both feature sets.

The final feature set for scE2G^{Multiome} consists of six features: ARC-E2G score, ubiquitous expression of promoter, number of candidate elements between element and gene, number of transcription start sites between element and gene, number of candidate elements within 5 kb of the element, and normalized ATAC signal at the promoter. The final feature set for scE2G^{ATAC} is the same, except with the ABC score in place of the ARC-E2G score.

9.3.3. *Applying scE2G to new cell types*: To apply the trained scE2G models to new cell types, we first compute a feature table for all candidate element-gene pairs as described above. We then apply the logistic regression model trained on all chromosomes to assign a score to each element-gene pair. We quantile-normalize the raw scores to a reference set of scores to maintain robustness across sequencing depths. The reference scores are scE2G scores from the model trained on all chromosomes computed on the full-depth K562 dataset before applying the gene expression threshold. For scE2G^{Multiome}, we then apply the gene

expression filter by setting the normalized score for any element-gene pair in which the gene is expressed below the designated TPM threshold to 0. For applications requiring binarized enhancer predictions, we used the score threshold determined as the score yielding 70% recall when evaluating the cross-validated scores on the CRISPR benchmark. The thresholds for scE2G^{Multiome} and scE2G^{ATAC} were determined to be 0.177 and 0.174, respectively.

10. Additional versions of scE2G

10.1. *scE2G downsample analysis:* We performed downsampling on Xu *et al.* K562[1] dataset to reduce the number of cells, the mean RNA UMI count, and the mean ATAC fragment count to the levels listed in **Extended Data Figure 7a–c**. These numbers, in addition to the ratio between ATAC fragment count and RNA UMI count, were chosen to cover the range of values represented in the other multiome datasets analyzed in this study. We used the `base::sample` function in R to select a specified number of cell barcodes. We then sampled the RNA count matrix based on a binomial distribution using the `stats::rbinom` function in R. For ATAC fragment downsampling, we used the `base::sample` function to extract a specific number of fragments. See https://github.com/anderssonlab/scE2G_analysis/blob/v1.2/4.Model_Interpretation/scripts/downsample.R for the full implementation.

We ran the scE2G pipeline on each downsampled dataset and assessed all scE2G prediction outcomes on the CRISPR benchmark. To determine the minimal sequencing depth requirement, we calculated the total ATAC fragments based on the mean ATAC fragment count multiplied by the cell count. Then, we explored the relationship between total ATAC fragments and AUPRC, precision, and recall (**Figure 4, Extended Data Figure 7**). We also trained scE2G models on each of the downsamples to examine the stability of optimal model weights across cell number and sequencing depth.

10.2. *scE2G meta-cell analysis:* We followed the SEACells[45] tutorials https://github.com/dpeerlab/SEACells/blob/main/notebooks/SEACell_computation.ipynb to construct 90 meta-cells based on Xu *et al.*[1] K562 scRNA-seq data. According to the single-cell to meta-cell assignments determined in SEACells (v0.3.3) (**Figure 3f**, **left**), we aggregated single-cell RNA UMI counts and ATAC fragment counts into meta-cell RNA UMI count matrix and ATAC fragment count matrix, respectively. We normalized the meta-cell RNA UMI count matrix using `Seurat::LogNormalize` and normalized the meta-cell ATAC fragment count matrix by dividing by the total counts of the corresponding meta-cell and then multiplying by 1,000,000. Based on the normalized RNA and ATAC matrices, we computed the Kendall correlation (meta-cell) using the `fastkendall::fastkendall` function for each candidate element-gene pair defined in the scE2G pipeline. Subsequently, we retrained a scE2G model using the ABC score and Kendall correlation (meta-cell) as independent features, along with five other features used in the original scE2G model. Finally, using the retrained model, we performed scE2G (meta-cell) predictions using a hold-one-chromosome-out cross-validation approach.

11. Published predictors

We run the published predictors on each 10x Multiome dataset using the same input RNA UMI count matrix and ATAC fragment count matrix, unless otherwise specified. We obtain the RNA UMI count matrix as described in **Supplementary Methods 6.1**. For datasets containing only one cell type (K562, GM12878, HCT116, Jurkat, and WTC11), we obtain the ATAC fragment count matrix as described in **Methods 7**. For datasets containing multiple cell types (PBMC and BMMC), we extracted MACS2 peaks that overlap with candidate enhancers defined in the ABC pipeline (**Supplementary Methods 9.1.1**) for each cell type, then merged peaks from all cell types, with peaks being merged if they overlap by at least 1 bp. Subsequently, we generated the ATAC count matrix using the `Signac::FeatureMatrix` function. For fair benchmarking, we uniformly make predictions for peak-gene pairs with a distance to TSS less than 1 Mb. We also conducted a version of the CRISPR benchmark using the default settings of each method on the Xu *et al.* K562 dataset (**Supplementary Figure 4**).

11.1. *Signac*[7]

Signac[7]: We ran Signac (v1.13.0) across all cell types within a dataset. For the uniform prediction within a 1Mb TSS distance, we normalized the RNA UMI count matrix using `Seurat::NormalizeData` function. We then calculated the Pearson correlation between normalized gene expression and enhancer accessibility for each candidate element-gene pair. To select background peaks, we employed `Signac::RegionStats` to compute GC content and region lengths for all peaks, and identified 200 background peaks with similar GC content, sequence length, and total ATAC fragment count using the `Signac::MatchRegionStats` function. Subsequently, we calculated the Pearson correlation between target gene expression and background peak accessibility. A z-score is computed for each enhancer-gene pair using the formula $z = (r-\mu)/\delta$, where μ is the mean background Pearson correlation and δ is the standard deviation of the background Pearson correlations for the peak. We then computed a p-value for each enhancer-gene pair using a one-sided z-test. See https://github.com/anderssonlab/scE2G_analysis/blob/v1.2/2.Prediction/K562_Xu/Signac/Signac.250210.ipynb for more details.

For the default setting prediction, we normalize the RNA UMI count matrix using the `Seurat::SCTransform` function, then conduct predictions using `Seurat::LinkPeaks` function. See https://github.com/anderssonlab/scE2G_analysis/blob/v1.2/2.Prediction/K562_Xu/Signac.default/Signac.250210.ipynb for more details.

11.2. *FigR*[8]

FigR[8]: We ran FigR (v0.1.0) across all cell types within a dataset. For uniform prediction within a 1Mb TSS distance, we normalized the RNA UMI count matrix using the `Seurat::NormalizeData` function, and the ATAC fragment count matrix using the `FigR::centerCounts` function. We then calculated the Spearman correlation between normalized gene expression and enhancer accessibility for each candidate enhancer-gene pair. To select background peaks, we used `chromVAR::addGCBias` to compute GC content for all peaks and identified 100 background peaks with similar GC content and total ATAC fragment count using the `chromVAR::getBackgroundPeaks` function. Subsequently, we calculated the Spearman correlation between target gene expression and background peak accessibility. A z-score for each enhancer-gene pair was computed using the formula $z = (r-\mu)/\delta$, where μ is the mean background Spearman correlation and

δ is the standard deviation of the background Spearman correlations for the peak. We then computed a p-value for each enhancer-gene pair using a one-sided z-test. See https://github.com/anderssonlab/scE2G_analysis/blob/v1.2/2.Prediction/K562_Xu/FigR/FigR.250211.ipynb for more details.

For the default setting prediction, we normalize the RNA UMI count matrix using the `Seurat::SCTransform` function and then conduct predictions using the `FigR::runGenePeakcorr` function. See https://github.com/anderssonlab/scE2G_analysis/blob/v1.2/2.Prediction/K562_Xu/FigR.default/FigR.250211.ipynb for more details.

11.3. *SnapATAC*[9]: We ran SnapATAC (v1.0.0) across all cell types within a dataset. For uniform prediction within a 1Mb TSS distance, we normalized the RNA UMI count matrix using the `Seurat::NormalizeData` function and binarized the ATAC fragment count matrix using the `Signac::BinarizeCounts` function. We then fitted a logistic regression model for each element-gene pair using `stats::glm(ATAC_accessibility ~ Gene_Expression, family = binomial(link = "logit"))`. See https://github.com/anderssonlab/scE2G_analysis/blob/v1.2/2.Prediction/K562_Xu/SnapATAC/SnapATAC.250212.ipynb for more details.

For the default setting prediction, we normalize the RNA UMI count matrix using the `Seurat::SCTransform` function and binarize the ATAC fragment count matrix using the `Signac::BinarizeCounts` function. Then, we conduct predictions using the `SnapATAC::predictGenePeakPair` function. See https://github.com/anderssonlab/scE2G_analysis/blob/v1.2/2.Prediction/K562_Xu/SnapATAC.default/SnapATAC.250212.ipynb for more details.

11.4. *SCENT*[10]: We ran SCENT (v1.0.0) for each cell type. For uniform prediction within a 1Mb TSS distance, we normalized the RNA UMI count matrix using the `Seurat::NormalizeData` function and binarized the ATAC fragment count matrix using the `Signac::BinarizeCounts` function. We then fitted a Poisson regression model for each element-gene pair using `stats::glm(exprs ~ atac + log.nUMI + percent.mito, family = "poisson")`, where exprs represents normalized gene expression, atac denotes binarized ATAC accessibility, log.nUMI is the log-transformed total RNA UMI count, and percent.mito is the percentage of mitochondrial UMIs. Subsequently, we performed bootstrapping using the `boot::boot` function and calculated the false discovery rate (FDR) using `SCENT::basic_p`. Due to the time-consuming nature of bootstrapping, we kept the bootstrapping process to a maximum of 50,000 iterations for K562, GM12878, HCT116, hWAT and Jurkat datasets, but limited the bootstrapping process to a maximum of 25,000 iterations for BMMC and PBMC datasets. To distinguish between activation and repression effects, we log-transformed the FDR and assigned it a negative value if the beta coefficient of ATAC accessibility in the Poisson regression model is less than zero. See https://github.com/anderssonlab/scE2G_analysis/blob/v1.2/2.Prediction/K562_Xu/SCENT/SCENT_500.250204.ipynb for more details.

11.5. *SCENIC+*[11]:We ran SCENIC+ (v1.0a1) across all cell types within a dataset. Following the SCENIC+ tutorials (https://scenicplus.readthedocs.io/en/latest/tutorials.html), we preprocessed scATAC-seq data using pycisTopic (see https://github.com/anderssonlab/scE2G_analysis/blob/v1.2/2.Prediction/K562_Xu/ScenicPlus/preprocessing.ATAC.ipynb) and scRNA-seq data using Scanpy[46] (see https://github.com/anderssonlab/scE2G_analysis/blob/v1.2/2.Prediction/K562_Xu/ScenicPlus/preprocessing.RNA.250317.ipynb). Subsequently, we used the commands `scenicplus prepare_data prepare_GEX_ACC` to prepare multiome input data, and `scenicplus prepare_data search_spance` to generate candidate element-gene pairs. Finally, we employed the `scenicplus grn_inference region_to_gene` command to make predictions. For uniform predictions, we configured `scenicplus prepare_data search_spance` with `--upstream 1000 1000000` and `--downstream 1000 1000000` to specify a maximum TSS distance as 1Mb. For default settings predictions, we adhered to the default parameters: `--upstream 1000 150000` and `--downstream 1000 150000`. See https://github.com/anderssonlab/scE2G_analysis/blob/v1.2/2.Prediction/K562_Xu/ScenicPlus/region_to_gene.250317.ipynb for more details.

11.6. *Cicero*[13]: We ran Cicero (v1.3.9) across all cell types within a dataset. We executed the Cicero pipeline according to the tutorial at https://cole-trapnell-lab.github.io/cicero-release/docs_m3/. For the uniform prediction, we configured the `cicero::estimate_distance_parameter` function with `distance_constraint = 1000000` and `window = 2000000` to specify the maximum TSS distance as 1Mb. For the default setting prediction, we adhered to the default parameters: `distance_constraint = 250000` and `window = 500000`. See https://github.com/anderssonlab/scE2G_analysis/blob/v1.2/2.Prediction/K562_Xu/Cicero/Cicero.240525.ipynb (Uniform Prediction) and https://github.com/anderssonlab/scE2G_analysis/blob/v1.2/2.Prediction/K562_Xu/Cicero.default/Cicero.240525.ipynb (Default Setting) for more details.

11.7. *DIRECT-NET*[15]: We ran DIRECT-NET (v0.0.1) across all cell types within a dataset. We ran the DIRECT-NET pipeline following the demo https://htmlpreview.github.io/?https://github.com/zhanglhbioinfor/DIRECT-NET/blob/main/tutorial/demo_DIRECTNET_PBMC.html. We adapted the `DIRECTNET::Aggregate_data` function for compatibility with the new version of Seurat, and modified the `DIRECTNET::Run_DIRECT_NET` function to enable setting the maximum TSS distance. We configured the maximum TSS distance to be 1,000,000 for uniform prediction and 250,000 for default setting prediction, respectively. See https://github.com/anderssonlab/scE2G_analysis/blob/v1.2/2.Prediction/K562_Xu/DIRECTNET/DIRECTNET.250213.ipynb (Uniform Prediction) and https://github.com/anderssonlab/scE2G_analysis/blob/v1.2/2.Prediction/K562_Xu/DIRECTNET.default/DIRECTNET.250213.ipynb (Default Setting) for more details.

11.8. *ArchR*[16]: We ran ArchR (v1.0.2) across all cell types within a dataset. We created an ArchR project following the tutorial available at https://www.archrproject.com/articles/Articles/tutorial.html. For the element-gene

predictions, we used the `ArchR::addPeak2GeneLinks` function as described in https://www.archrproject.com/bookdown/peak2genelinkage-with-archr.html. In the uniform prediction, we set `maxDist = 1000000` within the ArchR::addPeak2GeneLinks function to specify the maximum TSS distance as 1Mb, while for the default setting prediction, we adhered to the default `maxDist = 250000`. See https://github.com/anderssonlab/scE2G_analysis/blob/v1.2/2.Prediction/K562_Xu/ArchR/ArchR.250226.ipynb (Uniform Prediction) and https://github.com/anderssonlab/scE2G_analysis/blob/v1.2/2.Prediction/K562_Xu/ArchR.default/ArchR.250226.ipynb (Default Setting) for more details.

11.9. *ABC model*: We used the ABC scores of each cell type generated within our scE2G pipeline as described in **Methods 9.1**, which is implemented in the ABC pipeline.

11.10. *STARE (gABC model)*[17]: We ran STARE (v1.0.4) using the command "`STARE_ABCpp -b Enhancers.ATAC.tagAlign.sort.gz.CountReads.bedgraph -n 4 -a genes.gtf -w 10000000 -f false -t 0 -o [output_dir]`" for each cell type. The file `Enhancers.ATAC.tagAlign.sort.gz.CountReads.bedgraph` is generated in the scE2G pipeline, located at `[results_dir]/[cell_type]/[Neighborhoods]/Enhancers.ATAC.tagAlign.sort.gz.CountReads.bedgraph`. The first three columns of this file represent the coordinates of the enhancer (chr, start, end), and the fourth column contains the read count for the corresponding enhancer.

11.11. *Making cell-type specific predictions:* For benchmarks of published predictors that required predictions for each cell type, we obtained candidate enhancer-gene pairs for a specific cell type by filtering enhancer-gene predictions made on the full cell population to a specific cluster based on which peaks were accessible in that cell type and which genes were expressed in that cell type. For example, to make predictions for the BMMC erythroblast cell type, we filtered predictions made on the combined BMMC dataset to those which contained peaks accessible in erythroblasts and genes expressed in erythroblasts. For Cicero, we only filtered predictions based on peak accessibility since the model does not use gene expression data. We defined genes detected (UMI count > 0) in more than 1% cells for each cell type as "expressed genes," and defined MACS2 peaks that overlap with candidate enhancers identified in the ABC pipeline (**Supplementary Methods 9.1.1**) for each cell type as "accessible peaks."

12. CRISPR benchmark on training dataset

We used the CRISPR benchmarking pipeline developed in previous work[3] to compare predictive models to perturbation data from previous studies in which CRISPR was used to perturb candidate enhancers and effects on gene expression.

12.1. *CRISPR training dataset:* The CRISPR dataset is comprised of enhancer screens from 3 previously published CRISPR perturbation screen datasets conducted in K562 cells: 1) Nasser *et al.*, 2021[4] (itself comprised most of CRISPRi screens from Fulco et al., 2019[42]), 2) Gasperini *et al.*, 2019[5] (Perturb-seq) and 3) Schraivogel *et al.*, 2020[6] (TAP-seq). The CRISPR benchmarking pipeline is implemented here:

https://github.com/EngreitzLab/CRISPR_comparison/tree/AUPRC_trapezoid_add_left_rectangle. Some predictors might produce identical predicted scores, causing the left endpoint of the precision-recall curve to be far from the origin on the x-axis (e.g., SCENT). To ensure a fair comparison of AUPRC values, we calculated the area under the precision-recall curve using the trapezoidal method and included the area of the rectangle formed by the left endpoint of the curve and the origin (0,0).

To match the gene annotation used by cellranger-arc, we set the `gene_universe` and `tss_universe` parameters in the CRISPR comparison pipeline's config/config.yml file to "resources/genome_annotations/CollapsedGeneBounds.hg38.intGENCODEv43.bed" and "resources/genome_annotations/CollapsedGeneBounds.hg38.intGENCODEv43.TSS500bp.bed", respectively. Additionally, for CRISPR benchmarking with a restricted TSS distance of <1Mb, we set the `expt` parameter to "resources/crispr_data/EPCrisprBenchmark_ensemble_data_GRCh38.intGENCODEv43.1Mb.tsv.gz". For CRISPR benchmarking that includes all available enhancer-gene pairs, we set the `expt` parameter to "resources/crispr_data/EPCrisprBenchmark_ensemble_data_GRCh38.intGENCODEv43.tsv.gz".

13. Creating held-out CRISPR dataset

We collected and uniformly reprocessed data from 8 CRISPR perturbation screens covering 5 cell types to create a held-out dataset on which to benchmark our models. These datasets included: three Perturb-seq experiments in K562 (Xie *et al.*, 2019[47], Klann *et al.*, 2021[48], Morris *et al.*, 2023[49]); two enhancer screens performed in K562 and WTC11 using direct-capture targeted Perturb-seq (Ray *et al.*, 2025[50]); and element-gene pairs tested with CRISPRi-FlowFISH in K562 (Reilly *et al.*, 2021[51]), HCT116 (Guckelberger *et al.*, 2024[52]), GM12878 (Nasser *et al.*, 2021[4]), and Jurkat cells (Nasser *et al.*, 2021). Below, we describe the processing steps applied to each dataset.

13.1. Processing of Perturb-seq datasets

We reprocessed data for all Perturb-seq datasets (Xie *et al.*, 2019, Klann *et al.*, 2021, Morris *et al.*, 2023) using the methodologies described in the original publications. This included standard preprocessing steps: cell quality filtering based on UMI counts, mitochondrial transcript fraction, and gene detection thresholds specific to each dataset. For each dataset, we generated three core data matrices: (1) an mRNA gene expression counts matrix (genes × cells), (2) a guide RNA counts matrix (guides × cells), and (3) an annotation file describing each guide and its target element. To ensure consistent evaluation across all datasets, we defined a unified set of candidate elements using the ABC pipeline (**Supplementary Methods 9.1**) from K562 DNase-seq data (ENCODE experiment accession ENCSR000EOT). Only guides that overlapped with these candidate elements were retained for downstream analysis. The preprocessing details of the raw data for each Perturb-seq dataset is described below.

13.1.1. Processing of CRISPRi-Perturb-seq data from STING-seq v1 screen from Morris et al., 2023. We retrieved the data for the STING-seq v1 screen (the smaller pilot

screen from the study) from GSE171452, including count matrices for cDNA, cell-hashing (HTO), and guide-barcode (GDO). A Seurat object was initialised with the cDNA matrix; HTO and GDO matrices were added as separate assays. Cells were retained if their unique-gene count, total cDNA UMI count and mitochondrial transcript fraction lay between the 15th–99th, 20th–99th and 5th–90th percentiles, respectively. HTO counts were centre-log-ratio normalized and demultiplexed with HTODemux (positive.quantile = 0.99); only singlets were retained. The guide sequences were validated using UCSC's BLAT API, as the modest scale of the guide library permitted API-based validation without server limitations, with requirements for 100% sequence similarity to unique genomic locations. The guides were then overlapped with the K562 candidate elements described above.

13.1.2. Processing of CRISPRi-Perturb-seq data from STING-seq v2 screen from Morris et al., 2023. We retrieved the raw data for the STING-seq v2 screen (the larger, full-scale screen from the study) from GSE171452. We performed quality control separately for each lane: cells were retained if their unique-gene count lay between the 1st-99th percentiles, total cDNA UMI count between the 10th–99th percentiles, and mitochondrial transcript fraction between the 1st–90th percentiles. HTO counts were centre-log-ratio transformed and demultiplexed with HTODemux (positive.quantile = 0.99), retaining singlets. Because this experiment included 188 antibody-tagged oligonucleotides (ADT), the ADT profiles were further filtered to keep cells whose total ADT UMIs were within the 1st–99th percentile of the lane distribution. We found that batch B in the dataset had a dramatically lower distribution of UMIs than any of the other batches, so we removed those cells from further processing, then merged the three lane-specific Seurat objects. To validate guide sequences, we established a local BLAT server using the hg38 reference genome (hg38.2bit) with parameters -stepSize=5 for the server and -minScore=20 -minIdentity=0 for client queries, requiring 100% sequence similarity to unique genomic locations. The guides were then overlapped with the K562 candidate elements.

13.1.3. Processing of CRISPRi-Perturb-seq data from Xie et al., 2019. We obtained counts files from GEO accession GSE129837. For each batch we constructed a Seurat object and removed low-quality cells (unique-gene and total-UMI counts below the 10th percentile or above the 99th, and mitochondrial-read fraction >10%). Guides were filtered by alignment to hg38 using BLAT and overlap with the DNase-seq elements in K562 defined above. We validated guide sequences using the approach described above for STING-seq v2 from Morris *et al.*, 2023, then overlapped with the K562 candidate elements. The batch-level objects were merged to yield an mRNA expression matrix, a gRNA count matrix, and a metadata file.

13.1.4. Processing of CRISPRi-Perturb-seq data from Klann et al., 2021. We obtained the mRNA count matrix and the corresponding gRNA UMI matrix from ENCODE experiment ENCFF904ZDX. In line with the original analysis, the gene matrix was loaded into Seurat. Cells with >20 % mitochondrial UMIs or <10,000 total transcript UMIs were discarded, after which a single Seurat object was created containing

the mRNA counts assay and the gRNA counts assay. Genomic coordinates in hg19 for the guides provided by the authors were lifted to GRCh38 with easylift, retaining only guides with unambiguous positions. The lifted-over guides were then overlapped with the K562 candidate elements.

13.1.5. *Analyzing differential expression:* We performed differential expression analysis using SCEPTRE v0.10.0, a tool designed for single-cell CRISPR screen analysis. For each dataset, we tested every gene against all targeted elements within 1 Mb of the gene's transcription start site (TSS), with distances calculated using GENCODE v29 gene annotations. SCEPTRE was configured using the high MOI setting to account for cells containing multiple guides, and a union integration strategy to aggregate effects across multiple guides targeting the same element. We employed two-sided testing to detect both activating and repressing effects, and included dataset-specific batch information as covariates when available. The analysis workflow consisted of guide assignment using SCEPTRE's mixture model approach, followed by quality control filtering using SCEPTRE's default quality control parameters and discovery analysis using SCEPTRE's statistical framework.

13.1.6. *Calculating statistical power for each element-gene pair in Perturb-seq datasets:* To ensure robust negative controls and address potential false negatives due to insufficient statistical power, we implemented a comprehensive power simulation framework. This approach, originally developed using MAST and adapted for SCEPTRE compatibility, simulates perturbation effects at multiple effect sizes to estimate the statistical power of detecting true regulatory relationships. We tested effect sizes of 2%, 3%, 5%, 10%, 15%, 20%, 25%, and 50% decreases in gene expression across 100 simulation replicates per effect size to ensure robust power estimates. The simulations incorporated realistic guide-to-guide variability in knockdown efficiency , a parameter derived from previous experimental observations.

The power calculation process used mean expression and dispersion parameters ($1/\theta$ where θ is SCEPTRE's overdispersion parameter) for each gene, extracted from the original SCEPTRE analysis. For each cell-gene combination, the simulation used the guide assignments from the original SCEPTRE analysis, then assigned guide-specific effect sizes drawn from normal distributions ($\sigma = 0.13$ based on analysis of previous experiments). For guides targeting the perturbation of interest, effect sizes were sampled from distributions centered on the desired effect size, while control guides received effect sizes centered on no effect. The effect size matrix was then centered so that the average effect across all guides targeting a given perturbation equaled the specified effect size. Using these cell- and guide-specific effect sizes along with gene-specific parameters, synthetic UMI count matrices were generated via negative binomial sampling, followed by application of SCEPTRE differential expression analysis to the simulated data within each replication. For significance testing in the simulations, we used the largest nominal p-value that achieved significance after FDR correction in the original differential expression analysis as the threshold for calling effects

significant in the simulated data, in order to align with the significance thresholding on the real experiment. Power was computed as the proportion of simulations where both the p-value fell below this threshold and the effect size was negative, as all simulated effects were negative and any significant positive effects would be incorrect.

13.1.7. *Filtering pairs based on genomic location*. To identify element-gene pairs specifically testing distal enhancer-gene relationships rather than direct gene targeting effects, we annotated pairs based on the genomic features overlapping targeted elements. We annotated pairs with genomics features using GENCODE v29 annotation as follows:

Each perturbation element was overlapped with three genomic feature categories. First, we defined promoter regions as 1 kb upstream of the TSS for all annotated transcripts using the GenomicRanges promoters function. Second, we extracted all annotated exons to define gene body regions. Third, we calculated gene loci coordinates by taking the range of all exons per gene to capture complete gene bodies including intronic regions. We then performed overlap analysis between perturbation coordinates and these genomic features using findOverlapPairs, checking specifically whether each element overlapped the promoter or gene body of its target gene.

Element-gene pairs were removed if the targeted element overlapped a promoter region, regardless of the gene for that promoter. Similarly, pairs were removed if the targeted element overlapped any portion of the target gene's gene body, including both exonic and intronic regions, as this might have confounding effects due to RNA polymerase II interference. Only element-gene pairs passing all genomic feature filters were retained in the held-out dataset.

13.2. *Processing of direct-capture targeted Perturb-seq (DC-TAP-seq) datasets from Ray* et al.*, 2025*

We obtained the DC-TAP-seq datasets conducted in K562 and WTC11 from Supplementary Table 3 in Ray *et al.*, 2025. The element-gene pairs had already undergone SCEPTRE-based differential expression analysis and power simulation in the original study. We used the ‘significant’ column, which included positive control perturbations in the analysis, to define positive and negative pairs, in combination with the ‘power_at_effect_size_15’ to filter negatives for a minimum of 80% power to detect a 15% effect size. Consistent with the filtering based on genomic location for the other Perturb-seq datasets, we retained pairs classified as "DistalElement_Gene," which the authors defined by removing cases where the tested element overlapped the promoter or gene body of the target gene. We also filtered the results to retain only pairs with distances under 1 Mb to the TSS to align with the distance criteria used for other Perturb-seq datasets.

13.3. *Processing other datasets*

We obtained additional CRISPR enhancer perturbation results from the following published datasets. For these datasets, we used enhancer-gene links and applied statistical power filters, where available, as provided by the analyses of the respective studies.

13.3.1. *Nasser et al., 2021*. We extracted analyzed and statistical power filtered results on enhancer perturbations in GM12878 and Jurkat cells from: https://raw.githubusercontent.com/EngreitzLab/ABC-GWAS-Paper/main/comparePredictorsToCRISPRData/comparisonRuns/AllCellTypes-ABC_comparison/experimentalData/experimentalData.AllCellTypes.txt and reformatted them for our benchmarking framework.

13.3.2. *Guckelberger et al., 2024.* We obtained analyzed results including statistical power analysis on enhancer perturbations in control HCT116 from **Supplementary Table 2** and reformatted them for our benchmarking framework.

13.3.3. *Reilly et al., 2021.* We downloaded element-level quantifications for positives from CRISPRi tiling experiments in K562 from the ENCODE portal. We filtered the data based on genomic location to be consistent with the other datasets by removing element-gene pairs in which the element overlaps an annotated promoter or the target gene body (**Supplementary Methods 13.1.7**), then reformatted them for our benchmarking framework. Because this experiment did not allow for computing % effects on gene expression and because no power analyses were performed by the authors, we only retained significant element-gene pairs from this dataset and excluded pairs that did not have a significant effect.

14. Annotating elements with chromatin categories

We annotated the tested elements in training and held-out CRISPR datasets using H3K27ac, H3K27me3, and CTCF epigenetic datasets to characterize the chromatin states of the tested and positive element-gene pairs.

14.1. Processing input data

For K562, GM12878, HCT116, and WTC11, we downloaded BAM files and peak calls from the ENCODE portal. We also obtained DNase-seq data for Jurkat from the ENCODE portal. We filtered the BAM files according to ENCODE data processing pipelines depending on the assay and run type (https://www.encodeproject.org/pipelines/). For Jurkat, we downloaded FASTQ files from CTCF, H3K27ac, and H3K27me3 ChIP-seq experiments from SRA and prepared filtered BAM files peak calls according to ENCODE data processing pipelines. All epigenetic datasets used are listed here: https://github.com/mayasheth/chrom-annotate/blob/main/resources/metadata/epigenetic_datasets.tsv

14.2. Calculating chromatin/epigenetic features of tested elements.

To analyze the chromatin/epigenetic features of the tested elements, we first determined whether elements overlapped H3K27ac, H3K27me3, or CTCF ChIP-seq peaks in their respective cell types. H3K27ac and H3K27me3 peaks were extended by 175 bp on each

side due to the broad distribution of histone ChIP-seq signals. We also computed the H3K27ac RPM in each element expanded by 150 bp on each side. A pipeline implementing these annotations is available here: https://github.com/mayasheth/chrom-annotate

14.3. Categorizing elements into chromatin categories

We categorized elements according to the following ordered rules:

- If element overlaps H3K27ac peak:
  - H3K27ac RPM >90% for cell type → High H3K27ac element
  - Else → H3K27ac element
- Else if element does not overlap H3K27ac peak:
  - H3K27ac RPM >90% for cell type → H3K27ac high element
  - H3K27ac RPM >50% for cell type → H3K27ac element
  - Element overlaps CTCF peak → CTCF element
  - Element overlaps H3K27me3 peak → H3K27me3 element
  - Else → No H3K27ac element

15. Calculating direct and indirect effect rates

15.1. Calculating indirect and direct effects rates in CRISPR perturbation screens

The observed DE-gene pairs in CRISPR perturbation experiments contain both direct, cis-acting effects of element perturbations and indirect, trans-acting effects. To calculate the direct effects rate in the used CRISPR datasets, we first empirically estimated the indirect effects rates. We estimated the distance independent indirect effects rates for the K562, WTC11 and the uniformly reprocessed datasets (Xie *et al.* 2019; Klann *et al.* 2021; Morris *et al.* 2023; Gasperini *et al.* 2019) by systematically testing distal element perturbations for inter-chromosomal trans-acting effects. For each dataset we applied the same differential expression testing approach used to identify DE-gene pairs, but tested each distal element perturbation against 100 randomly selected genes on other chromosomes. Only genes in the *cis* discovery pairs (**Supplementary Methods 13.1.7**) were considered to ensure similar expression distributions of tested genes between cis- and trans-acting analyses. The dataset specific indirect effects rate was calculated as the proportion of positive trans-acting differential expression tests using the uncorrected p-value cutoff corresponding to the chosen FDR cutoff in each dataset (**Supplementary Methods 13.1.5**). This approach was used to calculate indirect effect rates for both positive and negative effects on expression.

To calculate the direct effects rate for each dataset, first we calculated the observed positive hit rate in the cis discovery analysis as a function of distance to TSS. The *cis* discovery pairs within 1Mb of the TSS were grouped into 50kb bins, and for each bin the positive hit rate was calculated as the proportion of positive tests for both positive and negative effects on expression. To calculate the rate of direct effects, for each distance bin the distance independent indirect effects rate was subtracted from the observed positive hit rate.

We calculated the average direct rate per distance bin across all used datasets and fitted a powerlaw function to model the direct effects rate as function of distance to TSS using

the `lm` function in R (4.2.0). Only distance bins with a direct rate greater than zero were included in this analysis. This model was then applied to predict the expected direct effects rate for all cis discovery pairs in all CRISPR datasets based on their distance to TSS.

15.2. Imputing indirect effect rates

For CRISPR datasets in both the training and held-out data where we could not empirically estimate an indirect effects rate, we used the average empirical indirect effects rate calculated as described above.

15.3. Calculating the probability of direct versus indirect CRISPR effects

The probability of each *cis* discovery pair being the result of a direct versus an indirect effect was calculated from the expected direct effects rate based on its distance to TSS and the datasets indirect effects rate:

$$p_{direct} = Probability\ direct\ vs.\ indirect\ effect$$
$$r_{direct} = Direct\ effects\ hit\ rate$$
$$r_{indirect} = Indirect\ effects\ hit\ rate$$
$$d = Distance\ to\ TSS$$

$$p_{direct}(d) = \frac{r_{direct}(d)}{r_{direct}(d) + r_{indirect}}$$

This approach was used to calculate probabilities of direct effects for both positive and negative effects on gene expression.

16. CRISPR benchmark on held-out dataset

16.1. Curating combined held-out dataset for benchmarking

To create the final held-out dataset on which to benchmark models, we combined all of the processed element-gene pairs, resolved any duplicated element-gene pairs tested in the same cell type, and removed pairs that were present in the training dataset. In order to obtain a clean set of negatives, we retained non-significant element-gene pairs that were tested with sufficient power to detect a change in gene expression. To obtain a clean set of positives likely to represent real enhancer interactions, we applied filters based on effect size and chromatin state at the tested element.

16.1.1. Combining all held-out datasets: We combined the individual processed and filtered sets of element-gene pairs into a single dataset. We first resized the tested elements from Ray *et al.*, 2025 (generally 300 bp) to 500 bp (+/- 250 bp from center) to be consistent with the rest of the dataset. When duplicate element-gene pairs existed within the same cell type, pairs were resolved using the following prioritization: (1) if only one pair contained a significant result for the pair, the significant result was retained; (2) if both datasets had the same significance status (both significant or both non-significant), the pair with higher statistical power at 15% effect size was retained.

16.1.2. *Removing pairs present in the training dataset.* To fairly evaluate the performance of the model on an unseen dataset, we removed any element-gene pairs that were already present in the training dataset. To do so, we removed any element-gene pairs from the held-out dataset tested in K562 that overlapped with element-gene pairs in the training dataset.

16.1.3. *Filtering element-gene pairs based on power analysis:* For the Perturb-seq and DC-TAP-seq datasets that had undergone power analysis with SCEPTRE, we retained negative element-gene pairs that 1) were non-significant in the original SCEPTRE analysis and 2) had at least 80% power detect a 15% effect size. We retained positives with an effect size greater than 5%. For the FlowFISH experiments from Nasser *et al.*, 2021 and Guckelberger *et al.*, 2024, we used power analysis results provided by the authors to retain negatives that had at least 80% power to detect a 25% effect size. We retained significant positives with any effect sizes.

16.1.4. *Filtering positives to likely enhancer interactions:* To ensure the positives in the CRISPR datasets represented enhancer-like regulatory mechanisms that the models are intended to predict, we removed positives with elements in the CTCF element, H3K27me3 element, or no H3K27ac categories. H3K27ac is a canonical marker of active enhancers, deposited as a result of transcription factor and cofactor binding that drives enhancer function. Positives lacking H3K27ac signal likely regulate gene expression through alternative mechanisms (such as CTCF-mediated chromatin looping or repressive histone modifications) that fall outside the scope of enhancer-gene linking models. By filtering out these cases, we focused the benchmark on regulatory elements that operate through the mechanisms that enhancer-gene linking models are designed to predict.

16.2. *Summarizing held-out CRISPR dataset*

The final held-out dataset used in the CRISPR benchmark is available https://github.com/EngreitzLab/CRISPR_comparison/blob/dev/resources/crispr_data/EPCrisprBenchmark_combined_data.heldout_5_cell_types.GRCh38.tsv.gz and is summarized as follows:

**Reference**	**Cell type**	**Negatives**	**Positives**	**Weighted positives**
Xie *et al.*, 2019	K562	399	42	37.1
Klann *et al.*, 2021	K562	12	21	18.9
Morris *et al.*, 2023	K562	149	35	29.1
Reilly et al., 2021	K562	0	8	7.4
Ray *et al.*, 2025	K562	1240	12	8.2

Ray *et al.,* 2025	WTC11	1906	15	13.4
Guckelberger *et al.*, 2024	HCT116	362	34	22.6
Nasser *et al.*, 2021	GM12878	52	16	14.3
Nasser *et al.*, 2021	Jurkat	68	7	6.3

16.3. Computing weighted performance metrics

To take into account the uncertainty of indirect CRISPR perturbation effects when benchmarking enhancer-gene prediction models, we computed precision-recall curves where each CRISPR element-gene pair was weighted by its probability to be a direct effect. Since enhancer-gene linking models are designed to predict direct regulatory relationships, weighting by the probability of direct effects ensures that our benchmark prioritizes true enhancer-gene connections while down-weighting confounding indirect effects that may artificially inflate or deflate model performance. Weighted precision-recall curves were computed using the pr_curve function from the yardstick (v=1.2.0) R package with case weights for each CRISPR element-gene pair set to the probability of a direct effect.

17. eQTL benchmark

We adapted our previously-developed eQTL benchmarking pipeline to assess whether predictive models of enhancer-gene regulatory interactions could (i) identify enhancers enriched for fine-mapped eQTL variants and (ii) link those enhancers to their corresponding eQTL target genes. We analyzed variants from the eQTL Catalogue V7 fine-mapped using the Sum of Single Effects (SuSiE) model[53]. The pipeline is implemented here: https://github.com/EngreitzLab/eQTLEnrichment/blob/integrated. Full results from the benchmarking analyses presented can be found here: https://github.com/anderssonlab/scE2G_analysis/tree/v1.2/3.Benchmarking/eQTL

17.1. *Fine-mapped eQTL variants:* We obtained the set of eQTL variants from the eQTL Catalogue V7 fine-mapped with SuSiE from untreated biosamples with RNA sequencing quantified by “gene expression” that were filtered to variants to those with eGenes expressed in their respective biosamples[53]. We retained variants to those with a target gene included in the gene reference file described in **Methods 2**. For all analyses, we considered variants with a posterior inclusion probability greater than 50%. We grouped variants based on their biosample of origin at “fine-grained” and “coarse-grained” resolutions and retained biosamples with at least 35 variants. For example, the “fine-grained” grouping includes biosamples “CD4+ T cells”, “CD4+ naive T cells”, and “CD8+ T cells,” while the “coarse-grained” grouping combines all of these into “T cells”. The code and table describing the collation of eQTL variants is available here: https://github.com/anderssonlab/scE2G_analysis/tree/v1.2/3.Benchmarking/eQTL/eQTL_Catalogue_v7

17.2. *Filtering to distal noncoding variants*: For eQTL benchmarking analyses, we focused on distal noncoding variants by filtering out any variants overlapping (i) coding sequences,

(ii) 5′ and 3′ untranslated regions of protein-coding genes, (iii) splice sites (within 10 bp of an intron–exon junction of a protein-coding gene) of protein-coding genes, and (iv) promoters (±250 bp from the gene TSS) of protein-coding genes. The disjoint genome partition used for this categorization is available here: https://github.com/EngreitzLab/eQTLEnrichment/blob/integrated/resources/genome_annotation/PartitionCombined.bed

17.3. *Defining prediction–eQTL cell type matches for eQTL benchmarking:* We assigned each cell type with predicted regulatory enhancer-gene interactions to one or more closely-related eQTL biosamples (**Supplementary Table 4**). In the "fine-grained" eQTL analysis (**Figure 2e**,**f**, **Extended Data Figure 5a,b,g**) the following number of matching cell types was used for each method: 67 pairs for scE2G^{Multiome}, scE2G^{ATAC}, ABC, in element (ABC) & distance to TSS; 64 pairs for SnapATAC, Signac, Cicero, FigR, SCENIC+, DIRECT-NET, and ArchR; 26 pairs for STARE and SCENT. For the "coarse-grained" eQTL analysis (**Extended Data Figure 5c–f,h**), 3 pairs of matching cell types were used for ENCODE-rE2G, and 13 pairs were used for all other models. To calculate aggregate metrics across all pairs, we summed the variant counts for each matched set of cell types. For plots displaying aggregate metrics, only predictors which were applied to all relevant cell types are shown.

17.4. *Calculating enrichment of eQTLs in predicted enhancers*: We defined enrichment as (fraction of distal noncoding eQTL variants with PIP > 50% that overlap predicted enhancers) / (fraction of distal noncoding 1000 Genomes Project SNPs that overlap predicted enhancers) (see **Figure 2e**). To calculate this enrichment, we obtained a list of approximately 10 million SNPs from the 1000 Genomes Project from the Price Group [54] then filtered them to the distal noncoding regions of the genome as described in **Supplementary Methods 17.2**. We calculated the number of variants from each eQTL biosample. Next, we calculated the number of eQTL variants from each biosample overlapping enhancer predictions defined by the suggested threshold in each biosample and the number of distal noncoding 1000 Genomes Project variants intersecting thresholded predictions in each biosample. The enrichment for each prediction cell type, P and eQTL cell type, Q intersection was then calculated as [number of distal noncoding variants in Q with PIP > 50% overlapping predicted enhancers in P / total number of distal noncoding variants with PIP > 50% in Q] / [number of distal noncoding 1000 Genomes Project SNPs overlapping P / total number of distal noncoding 1000 Genomes Project SNPs]. We calculated confidence intervals for enrichment using the analytical formula for confidence interval of the risk ratio[55].

17.5. *Calculating recall of eQTLs in predicted enhancers:* To calculate the recall of a predictive model in linking an eQTL variant to its eGene, we considered each pair of matching prediction and eQTL cell types (**Supplementary Table 4**). For each pair, we calculated the 1) total recall, defined as the fraction of eQTL variants overlapping predicted enhancers; 2) recall accounting for gene-linking, defined as the fraction of eVariant-eGene pairs in which the eVariant overlaps a predicted enhancer linked to the correct eGene; and 3) for the set of variants that overlap any predicted enhancer, the fraction of eVariant-eGene pairs in which the overlapping enhancer is linked to the variant's eGene. We

calculated the 95% confidence interval of recall using the Wilson score interval implemented with the “plus four rule”[56].

17.6. *Calculating enrichment-recall curves:* We calculated a range of predictor score values to construct enrichment-recall curves (**Figure 2e)** by defining 50 to 100 quantiles across the combined recall (considering gene-linking) of matching cell types for each predictive model with a continuous score. For each matching pair of prediction and eQTL cell types, we found the list of eVariants intersecting any predicted enhancer linked to the correct eGene, without applying a threshold to predictions. We compiled the variant-prediction intersections across all pairs of cell types. Then, we found the model score at the designated number of quantiles for predictions in this list, which estimate model scores required to achieve evenly-spaced recalls between 0 and the maximum recall for that model. These threshold values were used to binarize predictions to evaluate enrichment and recall as described above to generate enrichment-recall curves.

17.7. *Calculating enrichment at a specific recall:* To compare the enrichment of eQTLs in predicted enhancers at a target recall (as in **Figure 2f**), we first identified which threshold value out of all that were evaluated yielded a recall closest to the target value. If the recall at this threshold was within 10% of the target recall, the model is included in the comparison. The significance in the difference in enrichment between each pair of models was computed as using a two-side test on the difference in log relative risk[57] and p-values were adjusted with the Bonferroni correction to account for multiple comparisons.

18. GWAS benchmark

Using an approach taken in previous work,[3,4] we developed a GWAS benchmarking pipeline to evaluate predictive models based on (i) enrichment of fine-mapped GWAS variants and (ii) ability to link GWAS signals to causal genes (**Figure 2g–i**). For this benchmark, we used GWAS data for 94 traits from the UK Biobank. The pipeline is implemented here: https://github.com/EngreitzLab/GWAS_E2G_benchmarking/tree/main

18.1. *Fine-mapped GWAS variants*: We obtained GWAS summary statistics and fine-mapping results for 94 traits from the UK Biobank[25]. Detailed methods for the genetic association analysis, fine-mapping with SuSiE, and post-processing as well as the fine-mapping results are available at https://www.finucanelab.org/data. Definitions of each trait used in this study are summarized here: https://github.com/anderssonlab/scE2G_analysis/blob/v1.2/3.Benchmarking/GWAS/UKBB_94_traits.tsv. For all analyses, we retained variants with a fine-mapping posterior inclusion probability greater than 10% and that were located in distal noncoding regions of the genome as in **Supplementary Methods 17.2**.

18.2. *Evaluation set of causal genes for GWAS signals:* To compare how well predictive models link GWAS signals to causal genes, we used a “silver-standard” evaluation set developed in previous work using the fine-mapping results described above[Citation error]. Briefly, probable causal genes for each trait were defined as those containing a protein-coding, fine-mapped variant with a posterior inclusion probability greater than 50%. Then, each causal gene was linked to all independent noncoding credible sets for the same trait located within 500 kb. We removed duplicate credible sets in which the same noncoding

variants were linked to the same gene for multiple traits, to avoid duplicates in the benchmark. This evaluation set of locus-gene links is larger, available for more traits, and less biased towards well-studied genes than other alternatives. However, it is limited by the assumptions that genes with protein-coding variants are causal for the trait and that multiple GWAS signals in the same locus have the same causal gene.

18.3. *Defining biosample-trait pairs for GWAS benchmarking:* For the benchmarking evaluation, we assigned sets of biosamples to which the predictive models were applied with sets of GWAS traits expected to be relevant to those biosamples (**Supplementary Table 5**). For example, predictions in the five BMMC and PBMC clusters for CD14+ monocytes and CD16+ monocytes were evaluated against the monocyte count trait. For the variant overlap analysis, the variants for a set of traits paired with a set of prediction biosamples were deduplicated before evaluation. In both analyses, a variant was considered to overlap an enhancer if it intersected a predicted enhancer in at least one biosample at a given score threshold. In addition, a model was only evaluated for a given biosample-set, trait-set pair if it had been applied to all biosamples in the set.

18.4. *Calculating enrichment and recall of variants in predicted enhancers:* We calculated the recall of variants from trait (or set of traits) T in enhancer predictions from biosample (or set of biosamples) P as: [number of distal noncoding GWAS variants in T with PIP > 10% overlapping predicted enhancers in P / total number of distal noncoding variants with PIP > 10% in T]. We calculated the enrichment of variants from trait (or set of traits) T in enhancer predictions from biosample (or set of biosamples) P as: [number of distal noncoding GWAS variants in T with PIP > 10% overlapping predicted enhancers in P / total number of distal noncoding variants with PIP > 10% in T] / [number of distal noncoding 1000 Genomes Project SNPs overlapping P / total number of distal noncoding 1000 Genomes Project SNPs]. The 1000 Genomes Project SNPs were curated as described in **Supplementary Methods 17.4**. We calculated 95% confidence intervals for recall using the Wilson score interval implemented with the "plus four rule"[56] and for enrichment using the analytical formula for confidence interval of the risk ratio[55].

18.5. *Calculating enrichment-recall curves:* For each continuous predictive model, we calculated a range of model scores at which to threshold predictions. To do so, we first defined 25 thresholds evenly-spaced across the range of possible scores. Next, we found the predicted enhancer with the highest score in any biosample overlapping variants from all 94 traits, and calculated the scores at 25 quantiles for the predictions in this list to estimate thresholds required to achieve evenly-spaced recalls between 0 and the maximum recall for that model. These threshold values were used to binarize predictions to evaluate enrichment and recall as described above to generate enrichment-recall curves.

18.6. *Calculating precision and recall of identifying causal genes for GWAS signals:* We calculated the precision and recall of model predictions in each biosample and biosample set at identifying the causal genes associated with noncoding credible sets from each trait described in **Supplementary Methods 18.2**. After intersecting variants with enhancer-gene predictions binarized at the suggested thresholds, we defined credible set-gene predictions as each credible set linked to the two genes linked to enhancers overlapping

variants in the credible set with the highest scores (or, for binary predictors, all genes linked to enhancers overlapping variants in the credible set). Recall was calculated as (number of correct credible set-gene predictions / total number of credible sets). Precision was calculated as (number of correct credible set-gene predictions / total number of credible sets-gene predictions). 95% confidence intervals for both metrics were calculated using the Wilson score interval implemented with the "plus four rule"[56].

18.7. *Combining model predictions with PoPS*: To calculate precision and recall at identifying causal genes for each model in combination with the polygenic priority score (PoPS), we used the PoPS scores given to genes for each trait[58] to find the top two genes ranked by PoPS within 1 Mb of each noncoding credible set in the evaluation set. We then defined credible set-gene predictions for a model ∩ PoPS as each credible set linked to any gene that ranked in the top two based on both model score as in **Supplementary Methods 18.6** and PoPS score. Recall and precision were calculated as described above with this new definition of credible set-gene predictions.

19. Analyzing cell-type specificity of scE2G predictions

The code for all analyses of the cell-type specificity of scE2G predictions is available here: https://github.com/anderssonlab/scE2G_analysis/tree/v1.2/6.Cell_Type_Specificity/eQTL_enrichment_recall

19.1. *Computing Jaccard similarity between pairs of scE2G predictions:* We quantified overlap of predicted enhancer-gene links using the Jaccard similarity. We considered thresholded predictions (scE2G score ≥0.177 for scE2G^{Multiome}, no promoter elements) for each cell type. We computed the Jaccard similarity between cell type A and cell type B as: (number of shared predictions) / (number of total predictions for cell type A + number of total predictions for cell type B - number of shared predictions). We defined a "shared prediction" as enhancer-gene links with the same gene and at least a 50% overlap of each enhancer width.

19.2. *Computing Pearson correlation between pairs of scE2G predictions:* To compare the correlation of scE2G scores between predictions, we calculated the Pearson correlation between prediction scores in each pair of cell types. We considered only thresholded predictions and defined "shared predictions" as described above. For enhancer-gene predictions that were not shared between the two cell types, we set the score in the cell type without that prediction to 0. We then computed the Pearson correlation between the matched scores for each pair of cell types.

19.3. *Analyzing cell-type specificity of eQTL variant enrichment and recall*: we categorized each eQTL and prediction cell type pair as "same cell type" (*e.g.*, eQTLs from CD4+ T cells with predictions in any T cell type), "same cell lineage" (*e.g.*, eQTLs from CD4+ T cells with predictions in any lymphoid cell type), or "unmatched" (all other pairs). The categorizations used in **Figure 5b** and **c** can be found here: https://github.com/anderssonlab/scE2G_analysis/blob/v1.2/6.Cell_Type_Specificity/eQTL_enrichment_recall/biosample_matching_hd_pred_fine_eQTL.tsv.

19.4. *Computing number of predicted enhancer-gene links at different cell type resolutions:* To determine whether scE2G makes more enhancer-gene links for more fine-grained cell type while controlling for the number and size of the input cell clusters, we considered 15 main cell types from BMMCs which comprise five cell type "supergroups" (B, dendritic, erythroid, myeloid, T). We applied scE2G^{Multiome} to 15 samples of cells, size-proportional to the true cell type clusters, selected randomly from all BMMCs, the respective cell type supergroup, or the exact cell type. We then calculated the total number of unique enhancers identified across all BMMCs for each type of sample, using the same definition of "shared predictions" described in **Supplementary Methods 19.1**. We repeated this process across 20 iterations. The code used to adapt the scE2G pipeline for this analysis can be found here: https://github.com/anderssonlab/scE2G_analysis/tree/v1.2/6.Cell_Type_Specificity/permute_cell_types

20. Applying scE2G to prioritize causal genes and cell types for GWAS loci

20.1. *Computing enrichment of heritability of GWAS traits with stratified linkage disequilibrium score regression:* To estimate the enrichment of trait heritability in predicted enhancers of each cell type, we used stratified linkage disequilibrium score regression (S-LDSC, v1.0.1[23,59]). We considered all distal enhancers (removing any enhancers overlapping a promoter) predicted by scE2G^{Multiome} above the model threshold for each cell type. We prepared summary statistics for 104 traits[25] (GWAS summary statistics from the UK Biobank obtained from https://www.finucanelab.org/data), using the `munge_sumstats.py` script provided by LDSC and filtered to retain SNPs curated by HapMap 3 as recommended[60]. We performed S-LDSC using the scE2G^{Multiome} enhancers along with the baseline model (v2.2). We used the 1000 Genome EUR Phase 3 genotype data as the LD reference panel. Full results from S-LDSC can be found in **Supplementary Table 8**.

20.2. *Prioritizing causal genes and cell types for GWAS signals:* To systematically apply scE2G to prioritize causal genes and cell types for GWAS signals, we first determined which traits had variants that were 1) significantly enriched and 2) enriched at least 5-fold in predicted enhancer from which cell types. We considered blood-related traits for the 31 cell types from BMMCs and PBMCs, and metabolic-related traits for the 9 cell types from pancreatic islets. We found all credible set, cell type, gene combinations for which the credible set has noncoding variants located in a predicted scE2G^{Multiome} enhancer linked to that gene in that cell type. We recorded the maximum scE2G score supporting that prediction, as well as all other cell types in which the credible set was linked to that gene. We also annotated each credible set with the nearest genes based on distance. We then filtered the predictions to the two genes linked to each credible set with the highest maximum scE2G scores ("scE2G rank" of 1 and 2). The full gene prioritization results (not filtered based on scE2G rank) can be found in **Supplementary Table 9**.

20.3. *Prioritizing causal genes for GWAS traits with the polygenic priority score*: We used Polygenic Priority Scores (PoPS) as an orthogonal gene prioritization method to scE2G[58]. We used PoPS results obtained in our previous work[4].

20.4. *Quantifying variant effect on transcription factor motifs:* We quantified the effect of a variant on a given transcription factor motif using the match score computed with FIMO (v5.5.9)[61]. We obtained position-weight matrices for the transcription factor motif of interest from JASPAR[62,63], and input 101 base pair sequences centered on the reference and alternate alleles. The match scores reported correspond to the same window from each allele.

21. Data visualization

21.1. *Box plots*: Box plots are defined as follows: the middle line or point corresponds to the median; lower and upper hinges correspond to first and third quartiles; the upper whisker extends from the hinge to the largest value no further than 1.5 × IQR from the hinge (where IQR is the interquartile range, or distance between the first and third quartiles); and the lower whisker extends from the hinge to the smallest value, at most 1.5 × IQR of the hinge. Data beyond the end of the whiskers are outlying points and are plotted individually, unless box plots are shown on top of all data points or data distribution.

21.2. *Point intervals:* Histograms and violin plots containing point interval plots were made with ggdist (v3.3.3)[64]. The middle point corresponds to the median; the thick line corresponds to the quantile interval of the center 66% of data; the thin line corresponds to the quantile interval of the center 95% of data.

21.3. *Locus plots*: All locus plots were made with the GViz (v1.46.1)[65] and GenomicInteractions (v1.36.0)[66] packages. The arcs and heatmaps show predictions that are above the model threshold. The arc widths are scaled based on log10-transformed scE2G scores. The color of arcs and elements in the heatmap indicate the magnitude of the scE2G score. The rows of the heatmap are based on the similarity of genome-wide predictions across cell types, (**Methods 19.1**, **Methods 19.2**). Predictions for the combined datasets are added as the first three rows. The pseudobulk ATAC-seq signal tracks are plotted based on the normalized bigWig file generated in the scE2G pipeline, in which the ATAC signal is scaled by the number of total ATAC fragments (see `frag_to_norm_bigWig` rule here: https://github.com/EngreitzLab/scE2G/blob/v1.3/workflow/rules/frag_to_tagAlign.smk#L113).

Supplementary References

1. Xu, W. *et al.* ISSAAC-seq enables sensitive and flexible multimodal profiling of chromatin accessibility and gene expression in single cells. *Nat. Methods* **19**, 1243–1249 (2022).
2. Wang, K. *et al.* Simple oligonucleotide-based multiplexing of single-cell chromatin accessibility. *Mol. Cell* **81**, 4319–4332.e10 (2021).
3. Gschwind, A. R. *et al.* An encyclopedia of enhancer-gene regulatory interactions in the human genome. *bioRxiv* (2023) doi:10.1101/2023.11.09.563812.
4. Nasser, J. *et al.* Genome-wide enhancer maps link risk variants to disease genes. *Nature* **593**, 238–243 (2021).
5. Gasperini, M. *et al.* A genome-wide framework for mapping gene regulation via cellular genetic screens. *Cell* **176**, 1516 (2019).
6. Schraivogel, D. *et al.* Targeted Perturb-seq enables genome-scale genetic screens in single cells. *Nat. Methods* **17**, 629–635 (2020).
7. Stuart, T., Srivastava, A., Madad, S., Lareau, C. A. & Satija, R. Single-cell chromatin state analysis with Signac. *Nat. Methods* **18**, 1333–1341 (2021).
8. Kartha, V. K. *et al.* Functional inference of gene regulation using single-cell multi-omics. *Cell Genom* **2**, (2022).
9. Fang, R. *et al.* Comprehensive analysis of single cell ATAC-seq data with SnapATAC. *Nat. Commun.* **12**, 1337 (2021).
10. Sakaue, S. *et al.* Tissue-specific enhancer-gene maps from multimodal single-cell data identify causal disease alleles. *Nat. Genet.* **56**, 615–626 (2024).
11. Bravo González-Blas, C. *et al.* SCENIC+: single-cell multiomic inference of enhancers and gene regulatory networks. *Nat. Methods* **20**, 1355–1367 (2023).
12. Moerman, T. *et al.* GRNBoost2 and Arboreto: efficient and scalable inference of gene regulatory networks. *Bioinformatics* **35**, 2159–2161 (2019).
13. Pliner, H. A. *et al.* Cicero Predicts cis-Regulatory DNA Interactions from Single-Cell Chromatin Accessibility Data. *Mol. Cell* **71**, 858–871.e8 (2018).
14. Friedman, J., Hastie, T. & Tibshirani, R. Sparse inverse covariance estimation with the graphical lasso. *Biostatistics* **9**, 432–441 (2008).
15. Zhang, L., Zhang, J. & Nie, Q. DIRECT-NET: An efficient method to discover cis-regulatory elements and construct regulatory networks from single-cell multiomics data. *Sci Adv* **8**, eabl7393 (2022).
16. Granja, J. M. *et al.* ArchR is a scalable software package for integrative single-cell chromatin accessibility analysis. *Nat. Genet.* **53**, 403–411 (2021).
17. Hecker, D., Behjati Ardakani, F., Karollus, A., Gagneur, J. & Schulz, M. H. The adapted Activity-By-Contact model for enhancer-gene assignment and its application to single-cell data. *Bioinformatics* **39**, (2023).
18. Tropberger, P. *et al.* Regulation of transcription through acetylation of H3K122 on the lateral surface of the histone octamer. *Cell* **152**, 859–872 (2013).
19. Pradeepa, M. M. *et al.* Histone H3 globular domain acetylation identifies a new class of enhancers. *Nat Genet* **48**, 681–686 (2016).
20. Bergman, D. T. *et al.* Compatibility rules of human enhancer and promoter sequences. *Nature* **607**, 176–184 (2022).
21. Martinez-Ara, M., Comoglio, F., van Arensbergen, J. & van Steensel, B. Systematic analysis of intrinsic enhancer-promoter compatibility in the mouse genome. *Mol. Cell* **82**, 2519–2531.e6 (2022).
22. Martinez-Ara, M., Comoglio, F. & van Steensel, B. Large-scale analysis of the integration of enhancer-enhancer signals by promoters. (2024) doi:10.7554/elife.91994.2.

23. Finucane, H. K. *et al.* Partitioning heritability by functional annotation using genome-wide association summary statistics. *Nature Genetics* **47**, 1228–1235 (2015).
24. GitHub - bulik/ldsc: LD Score Regression (LDSC). *GitHub* https://github.com/bulik/ldsc.
25. Kanai, M. *et al.* Insights from complex trait fine-mapping across diverse populations. *medRxiv* 2021.09.03.21262975 (2021) doi:10.1101/2021.09.03.21262975.
26. O'Leary, N. A. *et al.* Reference sequence (RefSeq) database at NCBI: current status, taxonomic expansion, and functional annotation. *Nucleic Acids Res* **44**, D733–45 (2016).
27. Seal, R. L. *et al.* Genenames.org: the HGNC resources in 2023. *Nucleic Acids Res* **51**, D1003–D1009 (2023).
28. GENCODE - Human Release 29. https://www.gencodegenes.org/human/release_29.html.
29. GENCODE - Human Release 43. https://www.gencodegenes.org/human/release_43.html.
30. Chen, A. F. *et al.* NEAT-seq: simultaneous profiling of intra-nuclear proteins, chromatin accessibility and gene expression in single cells. *Nat. Methods* **19**, 547–553 (2022).
31. Luecken, M. D. *et al.* A sandbox for prediction and integration of DNA, RNA, and proteins in single cells. (2021).
32. Wang, G. *et al.* Integrating genetics with single-cell multiomic measurements across disease states identifies mechanisms of beta cell dysfunction in type 2 diabetes. *Nat. Genet.* **55**, 984–994 (2023).
33. Metzl-Raz, E. *et al.* Generation of a dual edited human induced pluripotent stem cell Myl7-GFP reporter line with inducible CRISPRi/dCas9. *Stem Cell Res.* **61**, 102754 (2022).
34. Siller, R., Greenhough, S., Naumovska, E. & Sullivan, G. J. Small-molecule-driven hepatocyte differentiation of human pluripotent stem cells. *Stem Cell Reports* **4**, 939–952 (2015).
35. Hao, Y. *et al.* Dictionary learning for integrative, multimodal and scalable single-cell analysis. *Nat. Biotechnol.* **42**, 293–304 (2024).
36. Hao, Y. *et al.* Integrated analysis of multimodal single-cell data. *Cell* **184**, 3573–3587.e29 (2021).
37. Zhu, Q., Conrad, D. N. & Gartner, Z. J. deMULTIplex2: robust sample demultiplexing for scRNA-seq. *Genome Biol.* **25**, 37 (2024).
38. Korsunsky, I. *et al.* Fast, sensitive and accurate integration of single-cell data with Harmony. *Nat. Methods* **16**, 1289–1296 (2019).
39. GitHub - samtools/htslib: C library for high-throughput sequencing data formats. *GitHub* https://github.com/samtools/htslib.
40. Kendall, M. G. A new measure of rank correlation. *Biometrika* **30**, 81–93 (1938).
41. Zhang, Y. *et al.* Model-based analysis of ChIP-Seq (MACS). *Genome Biol.* **9**, R137 (2008).
42. Fulco, C. P. *et al.* Activity-by-contact model of enhancer-promoter regulation from thousands of CRISPR perturbations. *Nat. Genet.* **51**, 1664–1669 (2019).
43. ATAC-seq Data Standards and Processing Pipeline. https://www.encodeproject.org/atac-seq/.
44. Amemiya, H. M., Kundaje, A. & Boyle, A. P. The ENCODE Blacklist: Identification of Problematic Regions of the Genome. *Scientific Reports* **9**, 1–5 (2019).
45. Persad, S. *et al.* SEACells infers transcriptional and epigenomic cellular states from single-cell genomics data. *Nat. Biotechnol.* **41**, 1746–1757 (2023).
46. Wolf, F. A., Angerer, P. & Theis, F. J. SCANPY: large-scale single-cell gene expression data analysis. *Genome Biol.* **19**, (2018).
47. Global Analysis of Enhancer Targets Reveals Convergent Enhancer-Driven Regulatory Modules. *Cell Reports* **29**, 2570–2578.e5 (2019).
48. Klann, T. S. *et al.* Genome-wide annotation of gene regulatory elements linked to cell fitness. *bioRxiv* (2021) doi:10.1101/2021.03.08.434470.
49. Morris, J. A. *et al.* Discovery of target genes and pathways at GWAS loci by pooled single-cell CRISPR screens. *Science* **380**, eadh7699 (2023).

50. Ray, J. *et al.* An unbiased survey of distal element-gene regulatory interactions with direct-capture targeted Perturb-seq. *bioRxiv* (2025) doi:10.1101/2025.09.16.676677.
51. Reilly, S. K. *et al.* Direct characterization of cis-regulatory elements and functional dissection of complex genetic associations using HCR-FlowFISH. *Nature genetics* **53**, (2021).
52. Guckelberger, P. *et al.* Cohesin-mediated 3D contacts tune enhancer-promoter regulation. *bioRxiv* (2024) doi:10.1101/2024.07.12.603288.
53. Kerimov, N. *et al.* A compendium of uniformly processed human gene expression and splicing quantitative trait loci. *Nature Genetics* **53**, 1290–1299 (2021).
54. Website. https://alkesgroup.broadinstitute.org/LDSCORE/baseline_v1.1_hg38_annots/.
55. Website. (https://sphweb.bumc.bu.edu/otlt/mph-modules/bs/bs704_confidence_intervals/bs704_confidence_intervals8.html.
56. Wilson, E. B. Probable Inference, the Law of Succession, and Statistical Inference. *Journal of the American Statistical Association* (1927).
57. Altman, D. G. & Martin Bland, J. Interaction revisited: the difference between two estimates. *BMJ : British Medical Journal* **326**, 219 (2003).
58. Weeks, E. M. *et al.* Leveraging polygenic enrichments of gene features to predict genes underlying complex traits and diseases. *Nat. Genet.* **55**, 1267–1276 (2023).
59. Gazal, S. *et al.* Linkage disequilibrium–dependent architecture of human complex traits shows action of negative selection. *Nature Genetics* **49**, 1421–1427 (2017).
60. GitHub - bulik/ldsc: LD Score Regression (LDSC). *GitHub* https://github.com/bulik/ldsc.
61. FIMO - MEME Suite. https://meme-suite.org/meme/doc/fimo.html.
62. JASPAR: An open-access database of transcription factor binding profiles. https://jaspar.elixir.no/.
63. Rauluseviciute, I. *et al.* JASPAR 2024: 20th anniversary of the open-access database of transcription factor binding profiles. *Nucleic Acids Res.* **52**, D174–D182 (2024).
64. Visualizations of Distributions and Uncertainty. https://mjskay.github.io/ggdist/index.html.
65. Hahne, F. & Ivanek, R. Visualizing Genomic Data Using Gviz and Bioconductor. *Methods Mol. Biol.* **1418**, 335–351 (2016).
66. Harmston, N., Ing-Simmons, E., Perry, M., Barešić, A. & Lenhard, B. GenomicInteractions: An R/Bioconductor package for manipulating and investigating chromatin interaction data. *BMC Genomics* **16**, 963 (2015).
