# Supplementary material for: Mapping enhancer–gene regulatory interactions from single-cell data
Source: Nat Genet. 2026 Aug 3;58(8):1941–52. doi: 10.1038/s41588-026-02695-8 (PMC13447104; doi:10.1038/s41588-026-02695-8)
Supplement: Supplementary file 2 [file 41588_2026_2695_MOESM2_ESM.pdf]

nature portfolio

Corresponding author(s): Jesse M. Engreitz and Robin Andersson

Last updated by author(s): 7/7/2026

Reporting Summary

Nature Portfolio wishes to improve the reproducibility of the work that we publish. This form provides structure for consistency and transparency in reporting. For further information on Nature Portfolio policies, see our Editorial Policies and the Editorial Policy Checklist.

Statistics

For all statistical analyses, confirm that the following items are present in the figure legend, table legend, main text, or Methods section.

n/a	Confirmed	
☐	☒	The exact sample size (*n*) for each experimental group/condition, given as a discrete number and unit of measurement
☐	☒	A statement on whether measurements were taken from distinct samples or whether the same sample was measured repeatedly
☐	☒	The statistical test(s) used AND whether they are one- or two-sided<br>*Only common tests should be described solely by name; describe more complex techniques in the Methods section.*
☐	☒	A description of all covariates tested
☐	☒	A description of any assumptions or corrections, such as tests of normality and adjustment for multiple comparisons
☐	☒	A full description of the statistical parameters including central tendency (e.g. means) or other basic estimates (e.g. regression coefficient) AND variation (e.g. standard deviation) or associated estimates of uncertainty (e.g. confidence intervals)
☐	☒	For null hypothesis testing, the test statistic (e.g. *F*, *t*, *r*) with confidence intervals, effect sizes, degrees of freedom and *P* value noted<br>*Give P values as exact values whenever suitable.*
☒	☐	For Bayesian analysis, information on the choice of priors and Markov chain Monte Carlo settings
☒	☐	For hierarchical and complex designs, identification of the appropriate level for tests and full reporting of outcomes
☐	☒	Estimates of effect sizes (e.g. Cohen's *d*, Pearson's *r*), indicating how they were calculated

Our web collection on statistics for biologists contains articles on many of the points above.

Software and code

Policy information about availability of computer code

Data collection	Raw data from the GEO repository were downloaded using sratoolkit (v3.0.0). Other raw data were downloaded using wget (version 1.19.5).
Data analysis	10x Multiome data were preprocessed using cellranger-arc (v2.0.2) or cellranger (v6.0.0) and cellranger-atac (v2.0.0). Quality control and cell-type annotation were conducted using the deMULTIplex2 R package (v1.0.1), Harmony (v1.2.0), and/or the Seurat (v5.0.3) and Signac (v1.13.0) pipelines as detailed in the Methods. We ran following published enhancer-gene linking methods: ArchR (v1.0.2), Cicero (v1.3.9), DIRECT-NET (v0.0.1), FigR (v0.1.0), SCENT (v1.0.0), Scenic+ (v1.0a1), Signac (v1.13.0), SnapATAC (v1.0.0), and STARE (v1.0.4). The following software packages were used for additional analyses and visualization: S-LDSC (v1.0.1); FIMO (v5.5.9); R packages ggdist (v3.3.3), GViz (v1.46.1), GenomicInteractions (v1.36.0).<br><br>The scE2G pipeline is available at: https://github.com/EngreitzLab/scE2G/tree/v1.3 (DOI: 10.5281/zenodo.18320555)<br>The CRISPR benchmarking pipeline is available at: https://github.com/EngreitzLab/CRISPR_comparison/tree/AUPRC_trapezoid_add_left_rectangle (DOI: 10.5281/zenodo.18320613)<br>The eQTL benchmarking pipeline is available at: https://github.com/EngreitzLab/eQTLEnrichment/tree/integrated (DOI: 10.5281/zenodo.18320671)<br>The GWAS benchmarking pipeline is available at: https://github.com/EngreitzLab/GWAS_E2G_benchmarking (DOI: 10.5281/zenodo.18320659)<br>Data analysis code for this study are available at: https://github.com/anderssonlab/scE2G_analysis/releases/tag/v1.2 (DOI: 10.5281/zenodo.18320688)

For manuscripts utilizing custom algorithms or software that are central to the research but not yet described in published literature, software must be made available to editors and reviewers. We strongly encourage code deposition in a community repository (e.g. GitHub). See the Nature Portfolio guidelines for submitting code & software for further information.

Data

Policy information about availability of data

All manuscripts must include a data availability statement. This statement should provide the following information, where applicable:

- Accession codes, unique identifiers, or web links for publicly available datasets
- A description of any restrictions on data availability
- For clinical datasets or third party data, please ensure that the statement adheres to our policy

The locations of the scE2G_Multiome prediction files are listed in Supplementary Table 6. The data sources and quality control thresholds used for each 10x Multiome sample are listed in Supplementary Tables 10 and 11, respectively. The K562 CRISPR perturbation data used for model training and benchmarking is derived from data published in refs. 12, 37, and 38, and is available at https://github.com/EngreitzLab/CRISPR_comparison/blob/main/resources/crispr_data/EPCrisprBenchmark_combined_data.training_K562.GRCh38.tsv.gz. The held-out CRISPR perturbation data is derived from data published in refs. 41–47 and is available at https://github.com/EngreitzLab/CRISPR_comparison/blob/dev/resources/crispr_data/EPCrisprBenchmark_combined_data.heldout_5_cell_types.GRCh38.tsv.gz. The fine-mapped eQTL benchmarking set was obtained from the eQTL Catalogue, v7 (https://www.ebi.ac.uk/eqtl/) as described in Methods. The UK Biobank fine-mapped GWAS variant benchmarking set was obtained from https://www.finucanelab.org/data. The K562 nuclei SLIC-CAGE data were obtained from https://www.ncbi.nlm.nih.gov/geo/query/acc.cgi?acc=GSE306952.

Research involving human participants, their data, or biological material

Policy information about studies with human participants or human data. See also policy information about sex, gender (identity/presentation), and sexual orientation and race, ethnicity and racism.

Reporting on sex and gender	In the design and execution of this study, sex (as a biological attribute) and gender (shaped by social and cultural circumstances) were not explicitly considered variables. Consequently, data related to sex and gender were not collected, and therefore, no sex- and gender-based analyses were performed.
Reporting on race, ethnicity, or other socially relevant groupings	In the design and execution of this study, we did not utilize any socially constructed or socially relevant categorization variables, such as race, ethnicity, gender, or other socially relevant groupings.
Population characteristics	In the design and execution of this study, we did not collect or analyze data on covariate-relevant population characteristics such as age, genotypic information, past and current diagnoses, and treatment categories.
Recruitment	Our study did not involve the recruitment of participants.
Ethics oversight	Our research focuses on developing enhancer-gene linking models and primarily utilizes computational methods and publicly available datasets. Given the nature of our work, our study does not involve direct interaction with human or animal subjects nor the use of personalized genetic information that could potentially identify individuals.

Note that full information on the approval of the study protocol must also be provided in the manuscript.

Field-specific reporting

Please select the one below that is the best fit for your research. If you are not sure, read the appropriate sections before making your selection.

☒ Life sciences ☐ Behavioural & social sciences ☐ Ecological, evolutionary & environmental sciences

For a reference copy of the document with all sections, see nature.com/documents/nr-reporting-summary-flat.pdf

Life sciences study design

All studies must disclose on these points even when the disclosure is negative.

Sample size	We trained the scE2G model using all available K562 CRISPRi datasets, which included 466 unique "positive" element-gene pairs and 9,876 "negative" element-gene pairs.<br>We performed a downsampling analysis to determine that the scE2G model requires input data consisting of more than 2 million total ATAC fragments, more than 1 million RNA UMIs, and more than 100 single cells.<br>We applied scE2G model to build maps of enhancer-gene regulatory interactions across 45 different cell types: the cell lines K562, GM12878, Jurkat, HCT116, and WTC11, and cell types from BMMC, PBMC, pancreatic islets (see Table S6 for cell number and sequencing depth information)
Data exclusions	In the pancreatic islets dataset, cell clusters expressing multiple markers were considered as doublets. Additionally, beta cells from one donor (A0027) exhibited notable batch effects. Both doublets and beta cell clusters with batch effects were excluded from downstream analyses. When benchmarking models on the held-out CRISPR dataset, we excluded positive element-gene pairs with less than 5% effect size and/or elements without H3K27ac signal to ensure the positives represented a clean set of enhancer-like interactions (described in Methods).
Replication	We verified the reproducibility of our models by running the scE2G models on three independent K562 datasets (Xu et al., Wang et al., and

Replication	this study), and benchmarking them against CRISPRi data. We consistently observed that the scE2G models outperformed previously published models. This consistent performance across multiple datasets confirms the reproducibility of our results.
Randomization	In the downsampling analysis, we used the base::sample function in R to select a specified number of cell barcodes. We then sampled the RNA count matrix based on a binomial distribution using the stats::rbinom function in R. For ATAC fragment downsampling, we used the base::sample function to extract a specific number of fragments.<br>When generating samples with permuted cell type labels, we randomized cell assignments using the random.sample function in python.
Blinding	Blinding was not necessary for these studies because it does not affect the training and prediction processes of the model.

Reporting for specific materials, systems and methods

We require information from authors about some types of materials, experimental systems and methods used in many studies. Here, indicate whether each material, system or method listed is relevant to your study. If you are not sure if a list item applies to your research, read the appropriate section before selecting a response.

Materials & experimental systems

n/a	Involved in the study	
☒	☐	Antibodies
☐	☒	Eukaryotic cell lines
☒	☐	Palaeontology and archaeology
☒	☐	Animals and other organisms
☒	☐	Clinical data
☒	☐	Dual use research of concern
☒	☐	Plants

Methods

n/a	Involved in the study	
☒	☐	ChIP-seq
☒	☐	Flow cytometry
☒	☐	MRI-based neuroimaging

Eukaryotic cell lines

Policy information about cell lines and Sex and Gender in Research

Cell line source(s)	K562 cells were obtained from authors of Fulco et al. Nature Genetics, 2019). Jurkat (accesson TIB-152) and HCT116 (accession CCL-247) cells were obtained from ATCC. WTC11 cells were obtained as a gift from Ioannis Karakikes.
Authentication	K562, Jurkat, HCT116, and WTC11 cells were authenticated by scRNA-seq
Mycoplasma contamination	K562, Jurkat, HCT116, and WTC11 cells were confirmed negative for mycoplasma contamination
Commonly misidentified lines (See ICLAC register)	N/A

Plants

Seed stocks	N/A
Novel plant genotypes	N/A
Authentication	N/A
